# The CsrA-FliW network controls polar localization of the dual-function flagellin mRNA in *Campylobacter jejuni*

**DOI:** 10.1038/ncomms11667

**Published:** 2016-05-27

**Authors:** Gaurav Dugar, Sarah L. Svensson, Thorsten Bischler, Sina Wäldchen, Richard Reinhardt, Markus Sauer, Cynthia M. Sharma

**Affiliations:** 1 Research Centre for Infectious Diseases (ZINF), University of Würzburg, Josef-Schneider-Str. 2/D15, Würzburg D-97080, Germany; 2 Department of Biotechnology and Biophysics, University of Würzburg, Am Hubland, Würzburg D-97074, Germany; 3 Max Planck Genome Centre Cologne, Max Planck Institute for Plant Breeding Research, Carl-von-Linné-Weg 10, Cologne D-50829, Germany

## Abstract

The widespread CsrA/RsmA protein regulators repress translation by binding GGA motifs in bacterial mRNAs. CsrA activity is primarily controlled through sequestration by multiple small regulatory RNAs. Here we investigate CsrA activity control in the absence of antagonizing small RNAs by examining the CsrA regulon in the human pathogen *Campylobacter jejuni.* We use genome-wide co-immunoprecipitation combined with RNA sequencing to show that CsrA primarily binds flagellar mRNAs and identify the major flagellin mRNA (*flaA*) as the main CsrA target. The *flaA* mRNA is translationally repressed by CsrA, but it can also titrate CsrA activity. Together with the main *C. jejuni* CsrA antagonist, the FliW protein, *flaA* mRNA controls CsrA-mediated post-transcriptional regulation of other flagellar genes. RNA-FISH reveals that *flaA* mRNA is expressed and localized at the poles of elongating cells. Polar *flaA* mRNA localization is translation dependent and is post-transcriptionally regulated by the CsrA-FliW network. Overall, our results suggest a role for CsrA-FliW in spatiotemporal control of flagella assembly and localization of a dual-function mRNA.

## Abstract (web summary)

The CsrA protein binds to and represses translation of certain bacterial mRNAs. Here, Dugar *et al*. show for the human pathogen *Campylobacter jejuni* that the major flagellin mRNA acts as both a target and a regulatory 'sponge' for CsrA, and is localized at the cell poles in a translation-dependent manner.

## Graphical abstract

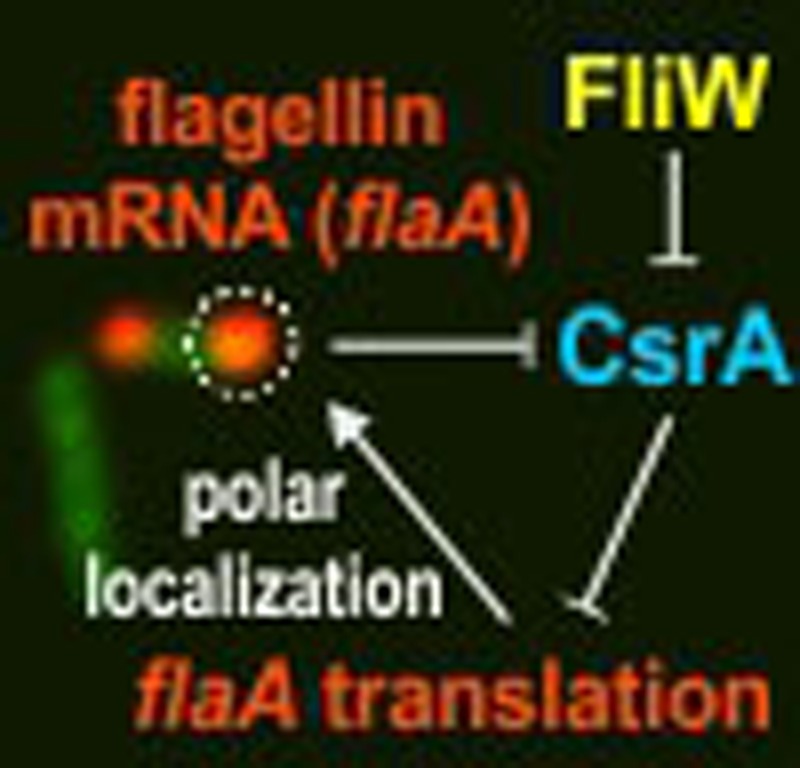

Post-transcriptional control involves a complex interplay between mRNAs, small regulatory RNAs (sRNAs) and protein regulators. Although regulatory functions have typically been attributed to proteins or sRNAs, mRNAs have canonically been considered as targets of this regulation. However, regulatory functions have recently also been described for mRNAs that either encode sRNAs in their untranslated regions (UTRs) or act as sponges that sequester other regulatory factors^1^,^2^,^3^,^4^.

The widespread bacterial Csr/Rsm (Carbon storage regulator/Regulator of secondary metabolism) regulatory network^5^ is an ideal model system to study the complex post-transcriptional cross-talk between mRNAs, sRNAs and protein regulators. About 75% of all sequenced bacterial genomes encode a homologue of the central RNA-binding protein (RBP) of this system, CsrA (RsmA/E). CsrA is a pleiotropic regulator of global physiological phenomena in Gammaproteobacteria^5^ and considered the most conserved post-transcriptional virulence regulator^6^. CsrA mainly acts by repression of translation initiation via binding to 5′ regions of mRNAs^7^. The homodimeric CsrA binds GGA-rich motifs that are often located in hairpin loops and/or overlap the Shine-Dalgarno (SD) sequence^5^. In Gammaproteobacteria, CsrA activity is regulated through the CsrB/C and RsmX/Y/Z families of sRNAs^5^,^7^. These antagonizing sRNAs are often induced by environmental signals^6^ and harbour multiple stem-loops with high-affinity GGA motifs that sequester CsrA/RsmA^8^. Despite the presence of CsrA, many bacteria lack homologues of these antagonizing sRNAs. Also, the global CsrA regulon and its general biological function outside the Gammaproteobacteria are unclear. In the Gram-positive *Bacillus subtilis*, the flagellar assembly protein FliW antagonizes CsrA via direct binding^9^. Although FliW homologues are relatively widespread^9^, protein-mediated regulation of CsrA has not yet been shown outside *B. subtilis*. Whether FliW can cooperate with RNA-mediated regulation of CsrA is also unknown.

In the Gram-negative Epsilonproteobacterium *Campylobacter jejuni*, currently the leading cause of bacterial gastroenteritis in humans, CsrA affects motility, biofilm formation, oxidative stress response and infection^10^. Despite several phenotypic analyses of *csrA* deletion strains^10^,^11^,^12^, direct CsrA targets in Epsilonproteobacteria are largely unknown. Global transcriptome studies indicated that both *C. jejuni* and the related pathogen *Helicobacter pylori*^13^,^14^,^15^,^16^, which both carry potential FliW homologues, lack the CsrA-antagonizing sRNAs.

Here we use co-immunoprecipitation (coIP) combined with RNA sequencing^17^,^18^ (RIP-seq) to globally determine the direct RNA-binding partners of *C. jejuni* CsrA and investigate whether RNA-based regulation of CsrA occurs in the absence of canonical antagonizing sRNAs. Our genome-wide approach reveals many mRNAs of flagellar genes as potential CsrA targets and we demonstrate that *flaA* mRNA, encoding the major flagellin, has dual (coding and regulatory) function. As the most abundantly co-purified transcript, *flaA* mRNA is the main target of CsrA translational repression. In addition, the *flaA* leader can act as an mRNA-derived RNA antagonist of CsrA. Together with the main CsrA antagonist, the FliW protein, *flaA* mRNA titrates CsrA to regulate expression of other flagellar genes.

In addition, using confocal and super-resolution microscopy imaging, we show that *flaA* mRNA is expressed in elongating cells and localizes to the cell poles of the amphitrichous *C. jejuni*. In contrast to eukaryotes^19^, RNA localization is so far only poorly understood in prokaryotes. Bacterial mRNAs can remain localized close to their genomic site of transcription^20^ or can migrate to places in the cell where their encoded products are required in a translation-independent manner involving *cis*-acting signals in the RNA itself^21^. Besides the mechanisms of bacterial RNA localization, even less is known about how this process may be regulated and which, if any, RBPs are involved. Here we show, based on a variety of *C. jejuni* mutants that disrupt or maintain *flaA* translation, that polar *flaA* mRNA localization requires its translation. Furthermore, we demonstrate that FliW facilitates polar flagellin mRNA localization by antagonizing CsrA-mediated translational repression of *flaA*. The unexpected role of the CsrA-FliW system in spatial control of flagellin mRNA expression provides new insight into the role of RBPs in bacterial mRNA localization, a process only recently described in prokaryotes.

## Results

### Global RIP-seq reveals direct CsrA targets in *C. jejuni*

**Figure 1:**
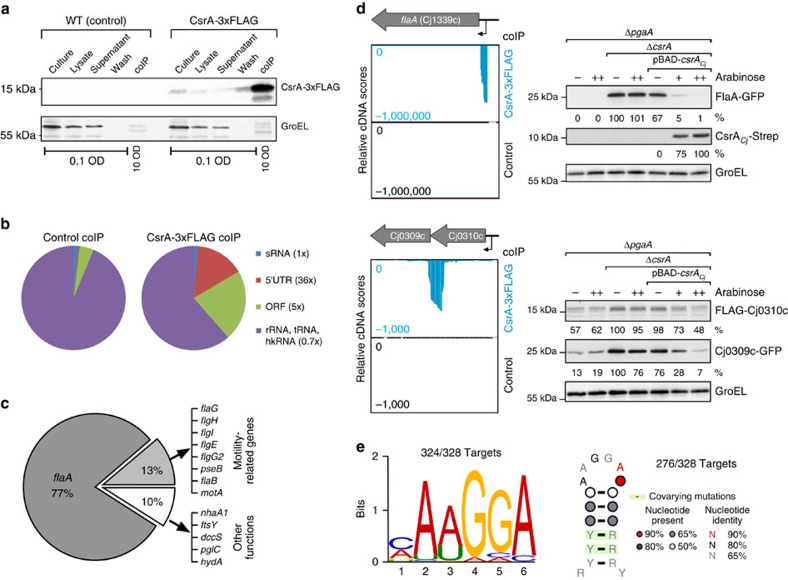
RIP-seq analysis of *C. jejuni* CsrA. (**a**) Western blot analysis of coIP samples of *C. jejuni* NCTC11168 WT and *csrA-*3xFLAG strains using anti-FLAG antibody confirms a successful CsrA-3xFLAG pulldown in the tagged strain. The amount of samples loaded (OD_600_ of bacteria) is indicated. GroEL served as loading control. (**b**) Pie charts showing relative proportions of mapped cDNA reads of different RNA classes in the coIP libraries (hkRNA: housekeeping RNAs). Numbers in brackets indicate the relative enrichment of the respective RNA class in the CsrA-3xFLAG versus control coIPs. (**c**) Pie chart showing the percentages and enriched genes of mapped reads for all >5-fold enriched CsrA target genes. (**d**) (Left) Mapped RNA-seq reads for the control (black) and CsrA-3xFLAG coIP (blue) in strain NCTC11168. Grey arrows: ORFs; black arrows: transcriptional start sites (TSS). Examples of enrichment patterns in 5′ UTRs (*flaA*) and between genes in a polycistron (Cj0310c-Cj0309c operon; encoding two paralogous efflux proteins). (Right) Western blot analysis using anti-FLAG and anti-GFP antibodies of reporter fusions to potential *C. jejuni* CsrA target genes in *E. coli* Δ*pgaA*, Δ*pgaA*/Δ*csrA* and Δ*pgaA*/Δ*csrA*+pBAD-*csrA*_*Cj*_ (complementation with *C. jejuni* CsrA-Strep under control of an arabinose-inducible pBAD promoter) strains. Putative CsrA targets from *C. jejuni* were fused in-frame (for example, 33 aa for *flaA*) to GFP or a FLAG-*lacZ'* tag (Supplementary Fig. 4b). As deletion of *csrA* dramatically enhanced biofilm formation and led to poor growth in liquid culture in our *E. coli* strain, reporter experiments were performed in a Δ*pgaA* background. GroEL served as loading control. Protein samples corresponding to 0.1 OD_600_ were loaded. Quantifications of reporter expression are given below the blots. (**e**) (Left) CsrA-binding motif predicted by MEME^24^ (*E*-value=2.1E-11). (Right) Consensus secondary structure motif of *C. jejuni* CsrA-binding sites predicted by CMfinder^62^.

To globally identify *C. jejuni* CsrA targets and any RNA regulators of CsrA activity, we applied a RIP-seq approach^17^,^18^. The *csrA* (Cj1103) gene was chromosomally 3xFLAG-tagged at its C-terminus in strains NCTC11168 and 81-176. CsrA-3xFLAG is constitutively expressed during growth in rich medium, and neither introduction of the FLAG-tag nor deletion of *csrA* affects *C. jejuni* growth under the examined conditions (Supplementary Fig. 1). We performed coIPs on mid-exponential-phase lysates of *csrA*-3xFLAG strains and, as control, their respective untagged wild-type (WT) strains (Fig. 1a and Supplementary Fig. 2a). After conversion of co-purified RNAs into cDNA and deep sequencing, 93.2–95.8% of the 4.6–6.2 million sequenced reads for the individual libraries were mapped to the respective genomes (Supplementary Table 1). Most of the NCTC11168 control-coIP library reads mapped to presumably non-specifically pulled-down abundant classes of RNA (rRNA, tRNA and housekeeping RNAs; Fig. 1b and Supplementary Table 2). In contrast, a ∼36-fold and ∼5-fold enrichment for reads mapped to 5′UTRs or open reading frames (ORFs) of mRNAs, respectively, was observed in the CsrA-3xFLAG coIP library (Fig. 1b). No specific sRNA enrichment was detected. As the coIP of strain 81-176 showed similar enrichment patterns (Supplementary Fig. 2b), we focused on strain NCTC11168.

### *C. jejuni* CsrA primarily binds flagellar mRNAs

Functional enrichment analysis of the 154 top CsrA targets with >5-fold enrichment in the CsrA-3xFLAG- versus control-coIP (Supplementary Data 1) revealed an overrepresentation of mRNAs from the class ‘Surface Structures', including flagellar genes (Supplementary Fig. 3a,b). In fact, 90% of the reads mapping to the >5-fold-enriched CsrA targets belonged to flagella- or motility-related genes (Fig. 1c). The alternative sigma factors RpoN (σ^54^) and FliA (σ^28^) hierarchically control flagellar expression in *Campylobacter*^22^. Early genes are expressed from RpoD/σ^70^-dependent promoters, whereas class 2 (middle) and class 3 (late) genes are RpoN- and FliA-dependent, respectively^22^. Most of the enriched transcripts belonged to either class 2 or class 3 (Table 1 and Supplementary Fig. 3c). The most abundantly co-purified transcript, with more than 300-fold enrichment, was *flaA* mRNA, encoding the major flagellin (Fig. 1c).

### cDNA peaks reveal CsrA binds in diverse mRNA regions

Visual inspection of the cDNA read-patterns showed that numerous flagellar mRNAs, including *flaA*, *flaG* and *flgI* (encoding the major flagellin, a gene involved in flagellum formation, and a P-ring component, respectively) showed strong enrichment in their 5′UTRs (Fig. 1d and Supplementary Fig. 4a). CsrA binding was also observed between two genes in polycistronic mRNAs, such as the Cj0310c-Cj0309c and Cj0805-*dapA* operons. Analysis of the potential CsrA-binding sites in an *Escherichia coli* green fluorescent protein (GFP) reporter-system, originally developed to study sRNA-mediated regulation^23^, revealed all of the tested 5′UTR targets (*flaA*, *flaG*, *flgI*, *flaB*, *pseB* and Cj1249) were highly upregulated (>10-fold) in the absence of *E. coli csrA* as measured by western blot and FACS analyses (Fig. 1d and Supplementary Figs 4 and 5). Reduced reporter fusion expression was restored by complementation of Δ*csrA* with *C. jejuni* CsrA. Using an operon reporter, where the C-terminal part of the upstream gene is fused to FLAG*-lacZ'* and the N-terminal part of the downstream gene to GFP, we observed that both *E. coli* and *C. jejuni* CsrA can repress the downstream genes in polycistrons (Cj0310c-Cj0309c and Cj0805-*dapA*). Expression of the upstream genes was only slightly affected and they do not contain any strong internal transcriptional start sites that could lead to uncoupled transcription of the downstream genes^14^. As we observed that potential SD sequences right at the 3′ end of the upstream genes are covered by CsrA target sites, CsrA probably interferes with ribosome binding and translation of the downstream genes and thereby might mediate discoordinate operon regulation.

### Automated peak-detection reveals a CsrA-binding motif

To automatically identify CsrA-binding regions and a binding motif from coIP cDNA enrichment patterns, we developed a peak-detection algorithm based on a sliding window approach (see the Methods for details). This approach predicted 328 potential CsrA-binding sites with >5-fold enrichment in the NCTC11168 coIP (Supplementary Data 2). As a control, peak detection was performed in reverse manner by scanning for enriched regions in the control- versus CsrA-3xFLAG-coIP. This analysis revealed only five peaks, without a common motif, indicating a high specificity of the peaks detected in the CsrA-3xFLAG-coIP. MEME^24^ analysis of the 328 enriched sequences revealed a (C/A)A(A/T)GGA motif in 324/328 input sequences (Fig. 1e). Analysis of the 81-176 coIP led to a similar motif (Supplementary Fig. 2c). To check if a similar motif can be found in non-enriched regions, we conducted the peak-detection in reverse manner using a cutoff of only >1-fold enrichment in the control- versus CsrA-3xFLAG-coIP. This revealed 448 ‘enriched' sites in the control library. Subsequent motif prediction did not yield any significant motifs, further supporting high specificity of the coIP approach. Consensus-structure motif screening of the enriched CsrA-coIP sequences revealed an AAGGA motif in a hairpin-structure loop in 276/328 input sequences (Fig. 1e). These *C. jejuni* sequence/structural motifs agree with binding sites of other CsrA homologues^25^.

### *flaA* mRNA is translationally repressed by CsrA

**Figure 2:**
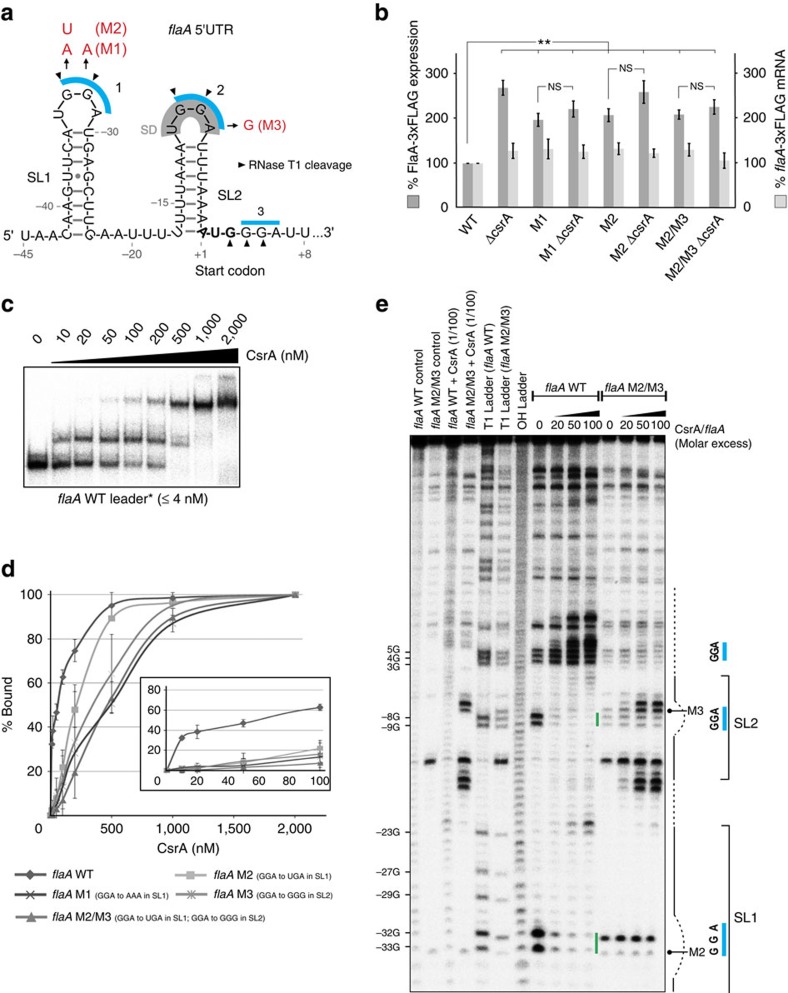
CsrA represses *flaA* translation by binding to its 5′UTR. (**a**) Predicted secondary structure of the *flaA* leader using Mfold^74^. Blue bars indicate GGA motifs; grey: SD sequence. Black triangles indicate RNase T1 cleavages from the structure probing in **c**. (**b**) Western blot quantification (*n*=5 biological replicates) of FlaA with a C-terminal 3xFLAG epitope tag integrated at its native locus (FlaA-3xFLAG) and northern blot analysis of *flaA* mRNA (*n*=3 biological replicates) in Δ*csrA* and various *flaA* 5′UTR mutant strains. Shown is the mean±s.e.m (**P<0.01 using Student's *t*-test, NS: not significant). Mutations are depicted in red in **a**. (**c**) Gel-shift assays using ∼0.04 pmol *in vitro*-transcribed and 5′ end-labelled *flaA* leader (−45 to +99 relative to the start codon) with increasing concentrations of CsrA. (**d**) Affinity binding curves determined by gel-shift assays for ^32^P-labelled *flaA* WT and mutant leaders (≤4 nM) based on three replicates. The inset represents an enlargement of the binding curves for low CsrA concentrations. Shown is the mean±s.d. (**e**) Footprinting assays of ∼0.2 pmol ^32^P-labelled *flaA* WT and *flaA* M2/M3 mutant leaders in the absence or presence of increasing CsrA concentrations (molar excess of 0, 20, 50 and 100 CsrA) using RNase T1. Untreated *flaA* leader alone or incubated with 100-fold excess of CsrA served as controls and RNase T1- or alkali (OH)-digested *flaA* leader as ladders, respectively. Blue lines: GGA motifs; green lines: protection from RNA cleavage upon addition of CsrA. The secondary structure of the *flaA* leader according to **a** is depicted on the right.

The flagellar filament, consisting mainly of the FlaA flagellin, is among the last components produced during flagellum assembly. In our coIP, 77% of the reads from >5-fold enriched genes mapped to *flaA,* indicating it as the main CsrA target (Fig. 1c). Secondary-structure predictions revealed that the 45-nt-long *flaA* 5′UTR can fold into two stem-loops (SL1 and SL2), both of which harbour an ANGGA motif in their loops (Fig. 2a). The second ANGGA motif covers the ribosome-binding site and a third GGA is present as the second codon. The *flaA* 5′UTR secondary structure is conserved and supported by compensatory base-pair changes in other *Campylobacter* species (Supplementary Figs 6 and 7, and Supplementary Methods). A chromosomally 3xFLAG-tagged FlaA was ∼3-fold upregulated in a Δ*csrA* strain compared with WT on western blots (Fig. 2b and Supplementary Fig. 8a, lanes 1 and 2). To show that CsrA affected translation by binding to the *flaA* leader, we introduced chromosomal point-mutations into the two putative GGA CsrA-binding motifs (M1: SL1_GGA→AAA_, M2: SL1_GGA→UGA_, and M3: SL2_GGA→GGG_; Fig. 2a,b and Supplementary Fig. 8a, lanes 3–8). Like deletion of *csrA*, mutation of the GGA motifs resulted in two- to threefold elevated FlaA-3xFLAG protein expression. FlaA-3xFLAG levels were not affected by deletion of *csrA* in the *flaA* leader mutants, indicating CsrA binding was abolished in these strains. Northern blot analysis showed *flaA*-3xFLAG mRNA levels are only mildly affected in the different mutant strains, further indicating post-transcriptional regulation of *flaA* by CsrA (Fig. 2b and Supplementary Fig. 8a).

*In vitro* gel-shift assays using recombinant *C. jejuni* CsrA-Strep and T7-transcribed, 5′-end radiolabelled *flaA* WT leader showed strong CsrA binding (*K*_d_=∼50 nM) with two defined shifts, indicating at least two CsrA-binding sites (Fig. 2c). In contrast, *flaA* leaders with GGA point-mutations in either SL1 (M1 and M2), SL2 (M3) or both SL1 and SL2 (M2/M3) showed four- to tenfold higher *K*_d_ values (200–500 nM), confirming that the mutations reduced CsrA binding (Fig. 2d and Supplementary Fig. 9a). To map CsrA-binding sites on the *flaA* leader, we performed *in-vitro* footprinting assays with labelled *flaA* leader in the absence or presence of CsrA using enzymatic and chemical cleavage (RNase T1; single stranded G-residues and lead(II) acetate; single-stranded RNA). Cleavage patterns without CsrA confirmed the predicted *flaA* leader structure (Fig. 2e and Supplementary Fig. 8b). A clear protection was observed at the SL1 and SL2 GGA motifs of the WT leader upon addition of increasing CsrA amounts, but not for a *flaA* M2/M3 mutant with disrupted binding motifs. The third GGA downstream of the start codon was not protected. Overall, our data suggest *C. jejuni* CsrA represses *flaA* translation by high-affinity binding to the two GGA-containing stem-loops SL1 and SL2 in the *flaA* leader.

### The flagellar assembly factor FliW binds CsrA in *C. jejuni*

**Figure 3:**
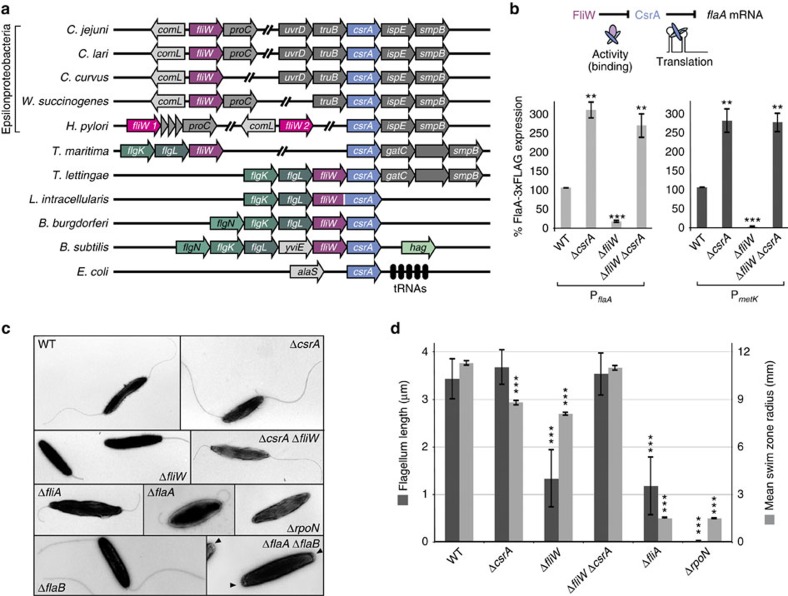
The flagellar assembly factor FliW binds and antagonizes CsrA. (**a**) Genomic context of *csrA* and *fliW* homologues in diverse bacterial species (*Campylobacter* spp: *C. jejuni, C. lari, C. curvus*; *Wolinella succinogenes*; *Helicobacter pylori*; Thermotogales: *T. maritima, T. lettingae*; *Lawsonia intracellularis*; *Borrellia burgdorferi*; *Bacillus subtilis*; *Escherichia coli*). Blue: *csrA* homologs; dark or light red: *fliW* homologues; shades of green: flagellar genes. (**b**) (Top) Scheme of the antagonizing effect of FliW on CsrA-mediated translational repression of *flaA* mRNA by direct binding of FliW to CsrA. (Bottom, left) Quantification of FlaA-3xFLAG using western blot in *C. jejuni* WT, Δ*csrA*, Δ*fliW* and Δ*csrA*/Δ*fliW* strains in mid-log phase (*n*=3 biological replicates). Plotted is the mean±s.e.m (**P<0.01, ***P<0.001 using Student's *t*-test). (Bottom, right) Quantification of FlaA-3xFLAG using western blot in WT, Δ*csrA*, Δ*fliW* and Δ*csrA*/Δ*fliW* strain backgrounds where the *flaA* promoter has been exchanged with the constitutive *metK* promoter. Please note that FlaA-3xFLAG levels expressed from the P_*metK*_ *promoter* represent ∼70% compared with the expression from its native P_*flaA*_ promoter. (**c**) Transmission electron micrographs of indicated strains harvested from MH agar. Black triangles indicate hook structures. (**d**) Average flagella length (dark grey bars) of indicated strains from transmission electron micrographs using ImageJ (*n*>25 measurements). Plotted is the mean±s.d. (***P<0.001 versus WT using Student's *t*-test). Motility was measured as average swimming distance (light grey bars) in soft agar. Bars show the mean±s.e.m (***P<0.001 versus WT using Student's *t*-test).

The constitutive expression of CsrA during routine culture (Supplementary Fig. 1) suggested modulation of its activity rather than its expression. Because homologues of the CsrB/C sRNAs are absent in *C. jejuni*, we hypothesized that other RNAs, or even proteins, might control CsrA activity in *Campylobacter*. One candidate (Cj1075, 129 aa) is a potential homologue of the flagellar assembly factor, FliW, which has a role in motility^26^,^27^ but is otherwise uncharacterized. In *B. subtilis*, FliW binds CsrA and antagonizes CsrA-mediated translational repression of *hag* mRNA, encoding the major flagellin^9^. FliW can also bind Hag, which accumulates in the cytoplasm before flagellar hook completion. Hag thus sequesters FliW from CsrA, allowing CsrA to repress Hag synthesis. Upon completion of the hook, Hag is secreted, FliW is released and CsrA repression of *flaA* translation is relieved. Thus, this Hag-FliW-CsrA partner-switch mechanism ensures appropriate temporal flagellin synthesis. In Epsilonproteobacteria, *fliW* homologues are present, but, unlike *Bacillus,* are not encoded adjacent to *csrA* (Fig. 3a). To investigate whether FliW can interact with CsrA and FlaA in *C. jejuni*, we performed protein–protein coIP experiments using chromosomal C-terminal 3xFLAG-tag fusions as bait. The anticipated interaction partners were tagged with mCherry at their C-terminus to allow detection by western blotting. In a FliW-3xFLAG-coIP, CsrA-mCherry was successfully co-purified, indicating the two proteins can interact (Supplementary Fig. 10). Similarly, FliW-mCherry was co-purified in a FlaA-3xFLAG-coIP, indicating conserved interactions between all three proteins. As control, none of the proteins was co-purified in coIPs with strains that carry the mCherry-fusion proteins but not the FLAG-tagged proteins.

### FliW antagonizes CsrA-mediated translational repression

To determine whether the FliW–CsrA interaction could antagonize CsrA function in Epsilonproteobacteria, we used FlaA protein levels as a read-out for CsrA activity (Fig. 3b). Whereas FlaA-3xFLAG was ∼3-fold upregulated in Δ*csrA*, deletion of *fliW* led to ∼6-fold downregulation, consistent with further repression of *flaA* translation by additional CsrA released upon deletion of its protein antagonist (Fig. 3b). A Δ*csrA*/Δ*fliW* double deletion confirmed that the observed downregulation was indeed mediated through CsrA, as FlaA-3xFLAG levels increased back to those in the Δ*csrA* mutant. Despite strong reduction of FlaA-3xFLAG protein levels, a ∼2-fold higher *flaA* mRNA level was observed upon deletion of *fliW*, indicating additional effects of FliW on *flaA* expression (Supplementary Fig. 11a). Thus, we constructed a transcriptional reporter composed of the unrelated Cj1321 5′UTR and its early coding region (Cj1321_mini) under the control of the *flaA* promoter. This reporter was, like the endogenous *flaA* mRNA, ∼2-fold upregulated in the Δ*fliW* mutant (Supplementary Fig. 11b). As Cj1321 is independent of CsrA-mediated control, FliW seems to have a negative effect (direct or indirect) on *flaA* transcription.

To uncouple transcriptional control of *flaA* from its translational regulation, we replaced the σ^28^-dependent *flaA* promoter in the FlaA-3xFLAG strain with a constitutive σ^70^-dependent *metK* promoter. Upon deletion of *csrA* in this strain, a ∼3-fold increase in FlaA-3xFLAG level was observed, further confirming post-transcriptional regulation of FlaA-3xFLAG protein expression by CsrA (Fig. 3b). Like for the strain expressing FlaA-3xFLAG from its native promoter, FlaA-3xFLAG expressed from the *metK* promoter was strongly downregulated upon deletion of *fliW* and was restored to Δ*csrA* levels in the Δ*csrA*/Δ*fliW* double mutant. This further indicates FliW antagonizes CsrA-mediated translational repression of *flaA* in a promoter-independent manner. In addition, decreased *flaA* mRNA stability was observed upon *fliW* deletion in rifampicin stability assays. This is consistent with increased translational repression of *flaA* in the absence of *fliW*, despite overall higher steady-state *flaA* mRNA levels because of FliW-dependent increased transcription (Supplementary Fig. 11c).

In line with strong downregulation of the FlaA protein upon *fliW* deletion, transmission electron microscopy revealed shorter flagella on Δ*fliW* bacteria compared with those of the WT strain (Fig. 3c,d). In fact, the flagella of Δ*fliW* appeared similar to those of a Δ*flaA* mutant strain and of bacteria lacking σ^28^ (Δ*fliA*), required for *flaA* transcription. In contrast, the Δ*csrA* and Δ*csrA*/Δ*fliW* strains expressed normal flagellar filaments. The short flagella of the Δ*fliW* strain are probably composed mainly of the minor flagellin FlaB, which is transcribed from an RpoN (σ^54^)-dependent promoter. Upon deletion of both flagellin genes (Δ*flaA/*Δ*flaB*), the bacteria no longer had filaments but the hook structure was visible at the poles (black arrowheads, Fig. 3c). Furthermore, a Δ*rpoN* mutant strain had neither flagella nor hooks. Motility assays revealed that the Δ*csrA* or Δ*fliW* strains showed a halo-radius reduction to 78% and 72% of WT, respectively (Fig. 3d). Likely due to its shorter flagella, Δ*fliW* also showed slower autoagglutination than WT, but greater than the non-motile Δ*fliA* and Δ*rpoN* mutants (Supplementary Fig. 12). Overall, these data suggest that, besides a mild effect on *flaA* transcription, FliW affects post-transcriptional control of FlaA, and therefore filament assembly and motility, in a CsrA-dependent manner.

### Expression of flagellar mRNAs is not affected in Δ*csrA*

**Figure 4:**
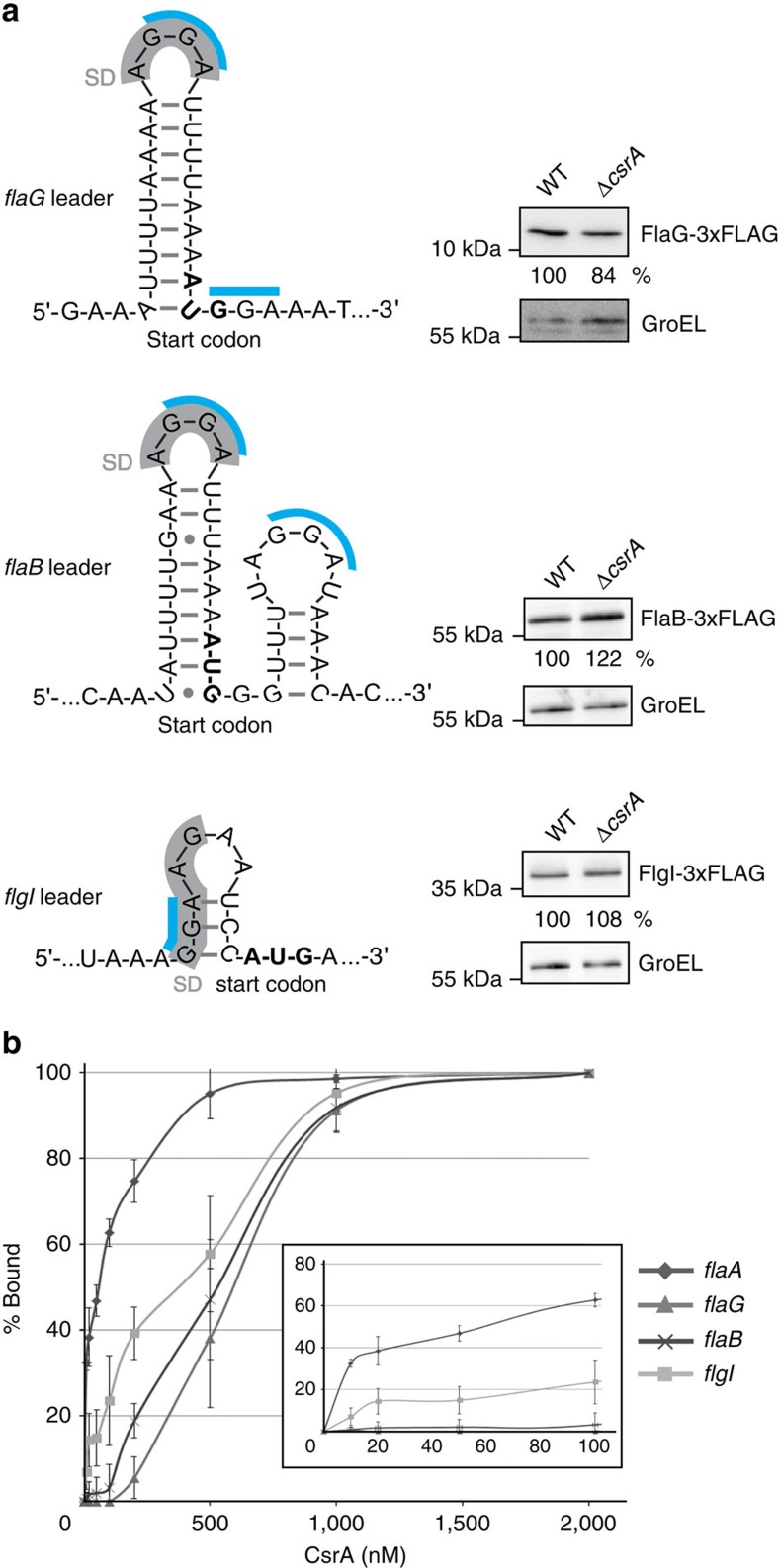
CsrA binds to other flagellar target mRNAs but *csrA* deletion does not affect their translation. (**a**) (Left) Predicted secondary structures of *flaG, flaB* and *flgI* leaders using Mfold^74^ with putative GGA binding-sites of CsrA (blue) and SD sequences (grey). (Right) Western blot analyses of FlaG-3xFLAG, FlaB-3xFLAG and FlgI-3xFLAG in *C. jejuni* WT or Δ*csrA* strains. (**b**) CsrA-binding affinities of flagella mRNA leaders (≤4 nM) determined by *in vitro* gel-shift assays. The inset represents an enlargement of the binding curves for low CsrA concentrations. Shown is the mean±s.d.

Besides *flaA* mRNA, many other flagellar targets, such as the 5′UTRs of *flaG*, *flaB* and *flgI*, were strongly enriched in the CsrA-3xFLAG-coIP (>346-, >58- and >170-fold, respectively; Table 1). The *flaG, flaB* and *flgI* leaders also have one or more GGA-containing motifs near their SD (Fig. 4a). *In vitro* gel-shift assays of *in vitro* transcribed *flaG, flaB* and *flgI* leaders, and several other co-purified flagellar mRNAs (Cj0040, *flgA* and *flgM*), confirmed CsrA binding (Fig. 4b and Supplementary Fig. 9b). The non-enriched Cj1324 mRNA, encoding a gene involved in flagellin modification, or an unrelated mRNA fragment from *H. pylori* did not shift with CsrA, confirming specific binding of CsrA to coIP-enriched transcripts (Supplementary Fig. 9c). However, CsrA affinity for *flaG, flaB* and *flgI* leaders was lower (*K*_d_=>350 nM) than for the *flaA* WT leader (*K*_d_=∼50 nM, Fig. 4b). Although FlaA-3xFLAG was upregulated upon *csrA* deletion (Fig. 2b), chromosomally tagged FlaG-3xFLAG, FlaB-3xFLAG and FlgI-3xFLAG levels did not change substantially (Fig. 4a).

### FliW and *flaA* mRNA titrate CsrA-mediated repression

**Figure 5:**
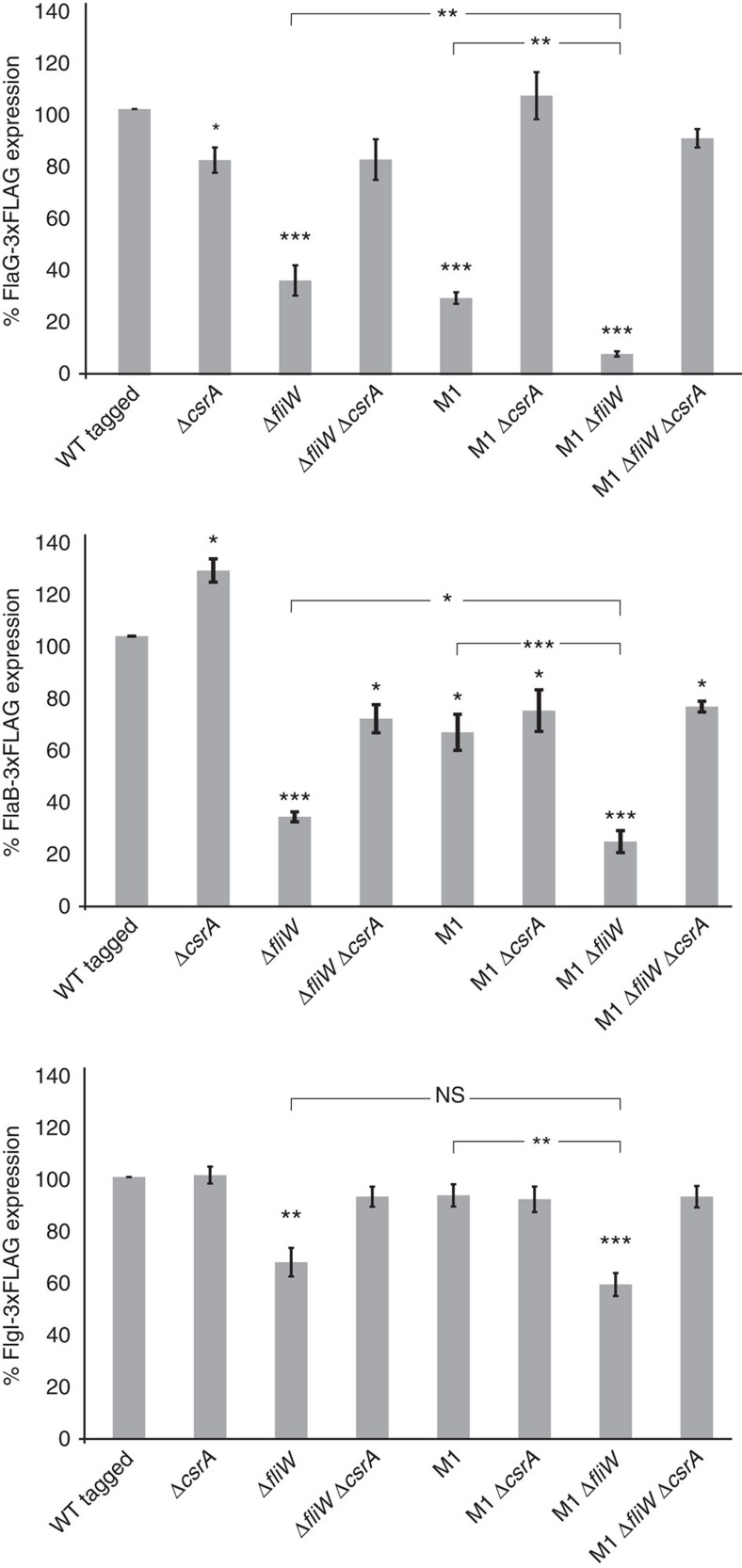
The *flaA* 5′UTR and FliW inhibit CsrA-mediated regulation of flagella genes. Quantification of FlaG-3xFLAG, FlaB-3xFLAG and FlgI-3xFLAG levels using western blot of the indicated *C. jejuni* NCTC11168 strains grown to mid-log phase (M1: GGA→AAA in SL1 of *flaA* 5′UTR). Values were calculated based on at least three biological replicates. Shown is the mean±s.e.m (*P<0.05, **P<0.01, ***P<0.001, using Student's *t*-test). NS, not significant.

The observed strong CsrA-mediated regulation of *flaG, flaB* and *flgI* in the *E. coli* reporter system (Supplementary Figs 4 and 5) indicates that CsrA can, in principle, regulate these targets. Thus, we hypothesized that FliW, or even abundant mRNAs, might sequester CsrA under the examined routine growth conditions, obscuring any regulatory effect on these low-affinity targets. Because *flaA* mRNA is highly abundant^14^ and expressed at the end of the flagellar cascade, we reasoned *flaA* mRNA might itself titrate CsrA activity. To investigate the role of FliW and the *flaA* mRNA as CsrA antagonists, we analysed FlaG-3xFLAG, FlaB-3xFLAG and FlgI-3xFLAG protein expression in loss-of-function strains of both antagonists. In line with FliW acting as a general CsrA antagonist that limits CsrA activity, deletion of *fliW* led to a ∼3-fold decrease in FlaG-3xFLAG level, which was restored to WT level in a Δ*csrA*/Δ*fliW* double mutant (Fig. 5 and Supplementary Fig. 13a).

Because *flaG* and *flaA* are primarily transcribed from σ^28^-dependent promoters^28^ and are thus expressed at the same time, monitoring FlaG-3xFLAG might reveal the potential role of *flaA* 5′UTR as a CsrA antagonist. The chromosomal M1 *flaA* leader mutation (GGA → AAA in SL1, Fig. 2a), which leaves the coding region intact but abolishes CsrA binding (Fig. 2d), decreased FlaG-3xFLAG levels ∼3-fold (Fig. 5 and Supplementary Fig. 13a). Upon introduction of Δ*csrA,* FlaG-3xFLAG expression was restored to WT levels, indicating decreased FlaG expression in the *flaA*-M1 mutant is dependent on CsrA, and suggesting that the *flaA* leader can also titrate CsrA. Combining both Δ*fliW* and *flaA-*M1 led to a tenfold reduction in FlaG-3xFLAG levels, showing their cumulative effect in antagonizing CsrA. In line with this, the M1/Δ*fliW*/Δ*csrA* triple mutant restored FlaG-3xFLAG levels back to WT levels (Fig. 5 and Supplementary Fig. 13a). Growth curves showed that there was no major impact on growth of the individual mutations under the examined conditions (Supplementary Fig. 13b). Although the Δ*fliW* and M1/Δ*fliW* mutants showed a slightly increased growth rate compared with WT, this increase was less than a non-motile Δ*fliA* strain.

To further confirm the role of the *flaA* 5′UTR as a CsrA antagonist, a ∼250-nt long *flaA_mini* transcript comprising the *flaA* leader and first 17 codons followed by a stable ribosomal *rrnB* terminator was ectopically expressed from the native *flaA* promoter (Supplementary Fig. 14a). Expression of the *flaA_mini* transcript in a Δ*fliW* mutant, which has strong CsrA-mediated *flaA* translational repression, increased FlaA-3xFLAG levels around 2.6-fold (Supplementary Fig. 14b). This indicates *flaA_mini* can bind and antagonize CsrA and partially relieve CsrA-mediated repression of *flaA* translation. A smaller, yet significant, complementation of the effect of a *fliW* deletion was also observed for FlaG-3xFLAG levels.

Next, the effect of the two antagonists on CsrA-mediated regulation of the RpoN-dependent genes *flaB* and *flgI* was evaluated. A similar, yet less pronounced effect compared with FlaG-3xFLAG, was observed for FlaB-3xFLAG upon single or double mutations of *fliW* and M1. In contrast, FlgI-3xFLAG levels were only significantly reduced upon *fliW* deletion (Fig. 5 and Supplementary Fig. 13a). Overall, this reveals FliW as the major CsrA antagonist under the examined growth conditions that titrates, along with the *flaA* mRNA antagonist, CsrA from lower affinity flagellar targets such as *flaG*.

### *flaA* mRNA localizes to the poles of elongating cells

**Figure 6:**
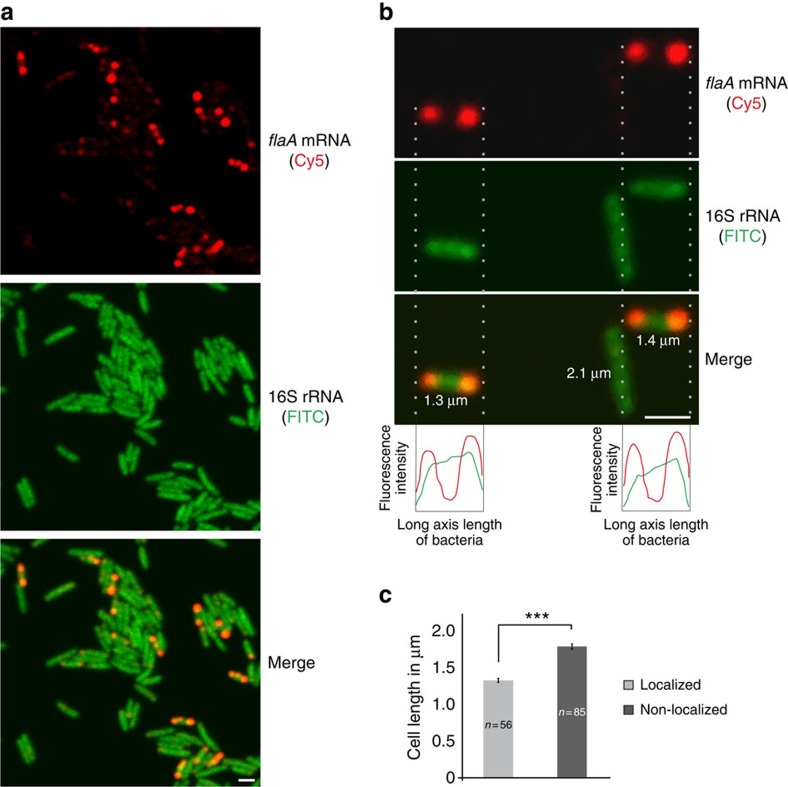
*flaA* mRNA localizes to the poles of shorter cells. (**a**) RNA-FISH analysis of 16S rRNA (FITC-labelled DNA oligonucleotide probe, green) and *flaA* mRNA (14 Cy5-labelled single-stranded DNA oligonucleotide probes, red) in *C. jejuni* WT cells in mid-log phase using confocal microscopy (scale bar, 1 μm). (**b**) A magnified RNA-FISH image showing the distribution of fluorescence signals. *flaA* mRNA (Cy5) and 16S rRNA (FITC) signals were quantified along the long axis length of bacteria using ImageJ software and were subsequently merged as shown at the bottom of the panel (scale bar, 1 μm). The length of individual cells was also quantified using ImageJ. Statistical analysis for average *flaA* mRNA and 16S rRNA signals over the cell length is provided in Supplementary Fig. 15. (**c**) Average *C. jejuni* WT cell lengths in bacteria where *flaA* mRNA is localized (56 cells) or non-localized (85 cells), ***P<10^−15^ using Student's *t*-test.

**Figure 7:**
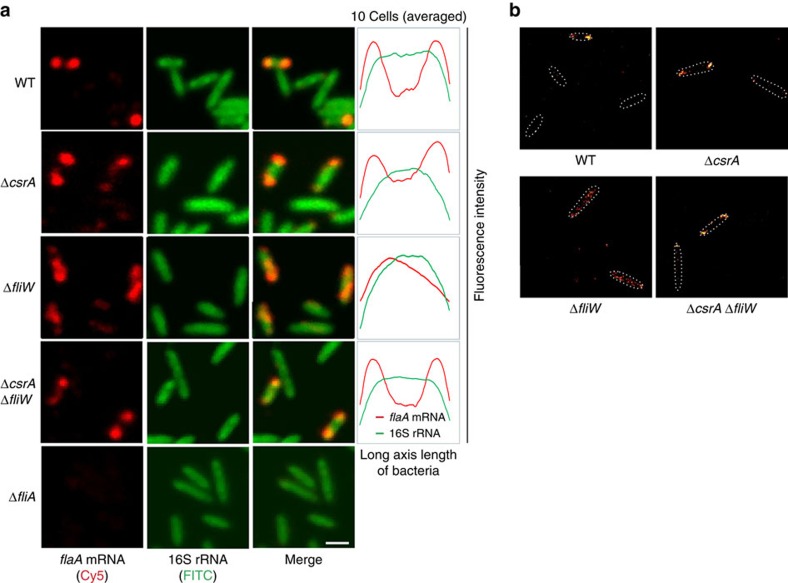
CsrA and FliW influence *flaA* mRNA localization to the poles. (**a**) RNA-FISH analysis (Left: confocal microscopy images; Right: averaged fluorescence intensity along the long axis based on 10 cells) of 16S rRNA (green) and *flaA* mRNA (red) in *C. jejuni* NCTC11168 WT, Δ*csrA*, Δ*fliW*, Δ*csrA*/Δ*fliW* and Δ*fliA* strains in mid-log phase. FITC and Cy5 channels were merged in the microcopy images in the third lanes (scale bar, 1 μm). (**b**) Super-resolution microscopy imaging of *flaA* mRNA RNA-FISH (14 Cy5-labelled oligos) in the indicated *C. jejuni* strains using *d*STORM imaging. Cell boundaries from bright-field images are depicted by white dotted lines.

As *flaA* mRNA can titrate CsrA activity, we wondered when *flaA* mRNA levels change to modulate CsrA activity. Expression of *flaA* mRNA appeared constitutive during growth (Supplementary Fig. 13c). However, in the amphitrichously flagellated *C. jejuni*, after every cell division, a new flagellum has to be synthesized at the new pole of each daughter cell. As bacteria in batch culture are not synchronized in cell cycle, differences in *flaA* mRNA expression might be obscured because of the population-based northern analysis. To monitor *flaA* mRNA expression in single bacteria, we performed RNA-FISH (fluorescence *in situ* hybridization) in fixed *C. jejuni* cells from exponential phase. Although the control RNA, 16S rRNA (Fig. 6a, green), was visible in all cells, *flaA* mRNA (Fig. 6a, red) was detected in only some of the cells. As a negative control, we also performed *flaA* mRNA FISH on a Δ*fliA* mutant strain (Fig. 7a), which showed no expression of *flaA* (Supplementary Fig. 11a). Whereas 16S rRNA was equally distributed throughout the cell, *flaA* mRNA was specifically detected at the cell poles in ∼20% of WT cells (Fig. 6a,b). Quantification of cell length across the population showed that cells with localized *flaA* mRNA were significantly shorter than cells without *flaA* expression (Fig. 6b,c). Live-cell imaging of a non-motile *C. jejuni* strain (Δ*fliA*) over two or three division cycles showed regular patterns of an increase in cell length until cells divide at mid-cell, resulting in short daughter cells (Supplementary Fig. 16). This indicates shorter cells likely correspond to cells that have divided and are elongating. Together, these data suggest differential expression of *flaA* mRNA during the cell cycle and accumulation in elongating cells at the required site of its encoded protein.

### FliW impacts *flaA* mRNA localization via CsrA

To investigate whether CsrA-FliW impacts *flaA* mRNA localization, we next performed RNA-FISH in Δ*fliW*, Δ*csrA* and Δ*fliW*/Δ*csrA* mutant strains. Although *csrA* deletion had no effect on *flaA* localization, it was completely abolished in a Δ*fliW* mutant (Fig. 7a). Instead of a polar localization, *flaA* mRNA was now dispersed throughout the cell. The loss of *flaA* mRNA localization upon *fliW* deletion was not due to lower transcript abundance as its mRNA level is increased despite strong repression at the protein level (Supplementary Fig. 11a). Strikingly, *flaA* mRNA localization was restored to the cell poles in the Δ*fliW*/Δ*csrA* double mutant, showing CsrA affects localization of *flaA* mRNA. As a further confirmation of *flaA* mRNA localization, we performed super-resolution imaging of *flaA* mRNA FISH in WT and mutant strains using *direct* stochastic optical reconstruction microscopy (*d*STORM)^29^, which has only recently been applied for bacterial RNA localization^30^. *d*STORM analysis fully supported and complemented the observations from confocal microscopy analysis (Fig. 7b and Supplementary Fig. 17). Overall, this suggests a model where *flaA* translation is required for polar localization: upon deletion of *fliW,* CsrA is released and in turn strongly represses *flaA* mRNA translation to impede its localization to the poles.

### Polar *flaA* mRNA localization requires its translation

**Figure 8:**
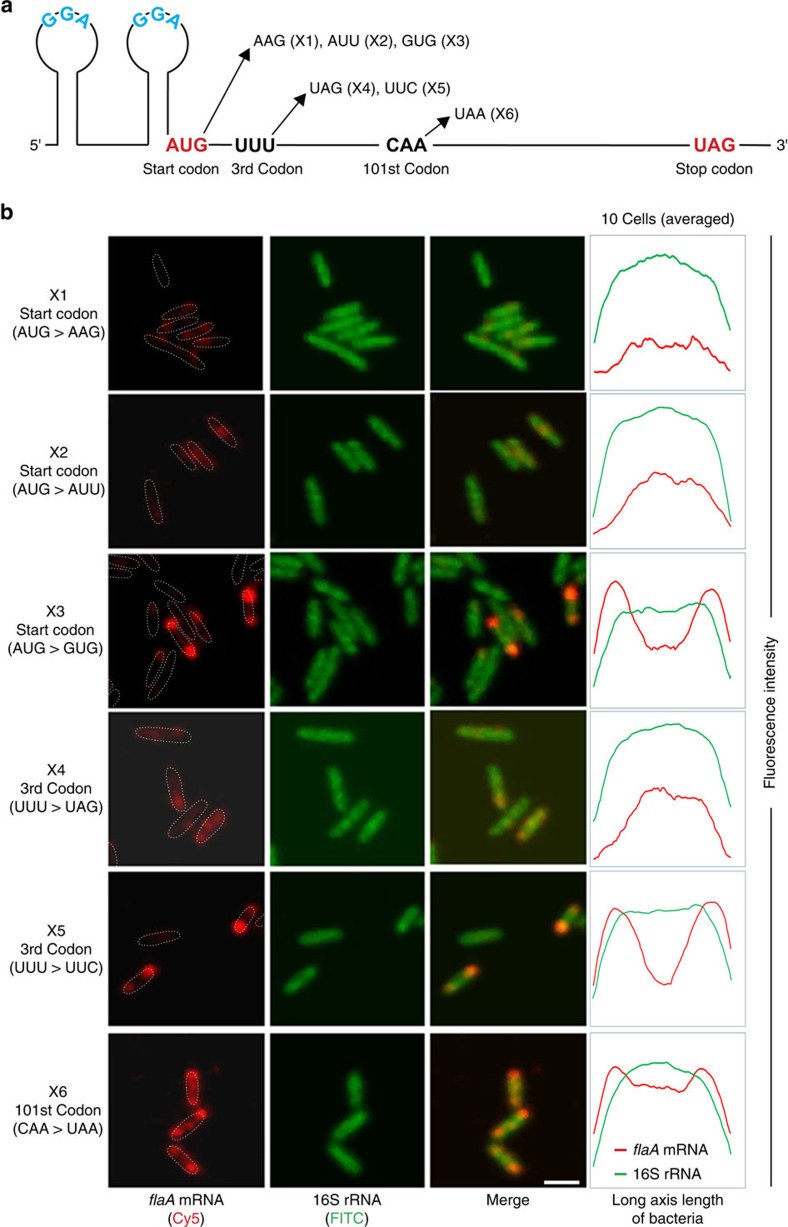
Translation is required for *flaA* mRNA localization to the cell poles. (**a**) Point mutations in *flaA* mRNA that were introduced at the native *flaA* locus. Mutations X1, X2, X4 and X6 abolish or prematurely stop *flaA* translation, whereas X3 and X5 represent silent mutations. (**b**) RNA-FISH analysis (Left: confocal microscopy images; Right: averaged fluorescence intensity along the long axis based on 10 cells) of *C. jejuni* point mutant strains depicted in **a**. FITC and Cy5 channels were merged in the third rows of the microscopy images (scale bar, 1 μm).

To support the translation-dependent model of *flaA* localization, we constructed several point mutants in the native *flaA* gene that either maintain or disrupt *flaA* translation (Fig. 8a). Mutation of the start codon of *flaA* (AUG → AAG (X1) or AUU (X2)) to abolish translation initiation resulted in dispersed *flaA* mRNA (Fig. 8b). In contrast, when the start codon was changed to an alternative start codon (AUG → GUG (X3)), *flaA* mRNA still localized to the cell poles, indicating translation of *flaA* mRNA is indeed required for polar localization. Mutation of the third *flaA* codon to a stop codon (UUU → UAG (X4)) also resulted in a completely dispersed *flaA* mRNA signal (Fig. 8b and Supplementary Fig. 17). In contrast, *flaA* mRNA with a synonymous silent mutation (UUU → UUC (X5); both encoding Phe) at the third codon localized similarly to the WT mRNA. Some of the mutations that abolish translation (X1, X2) lead to reduced (50–80% of WT) *flaA* mRNA levels (Supplementary Fig. 18). Nonetheless, as the strain expressing the *flaA* mRNA with a stop mutation at the third codon (X4), which also showed abolished polar mRNA localization, had even higher (∼170%) *flaA* expression levels than WT, it is unlikely that reduced (or increased) *flaA* mRNA levels lead to loss of localization. To determine the effect of terminating translation at a downstream position, we introduced a stop codon at the 101^st^ codon of *flaA* (CAA → UAA (X6)). This mutant showed partial polar *flaA* mRNA localization, suggesting the N-terminal peptide might be required for recruiting *flaA* mRNA to the cell poles. Overall, these data support a role of the FliW/CsrA post-transcriptional network in controlling translation-dependent polar *flaA* mRNA localization in *C. jejuni*.

## Discussion

**Figure 9:**
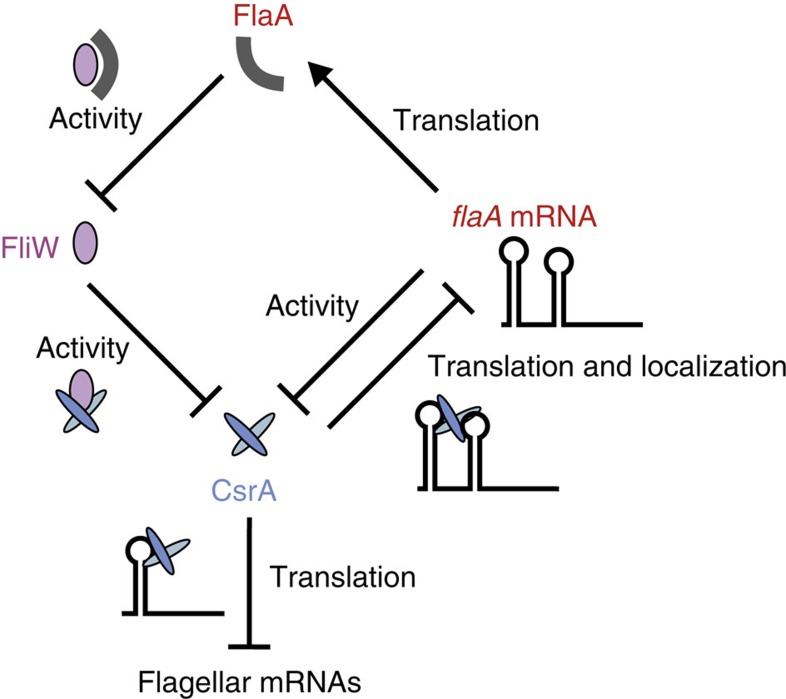
Model depicting the *C. jejuni* CsrA-FliW regulatory network. Schematic representation of the regulatory circuit and the putative roles of CsrA, FliW and FlaA proteins along with *flaA* mRNA in the CsrA-FliW regulon of *C. jejuni*. The post-transcriptional regulatory protein CsrA represses translation of multiple flagellar mRNAs including *flaA* mRNA, encoding the major flagellin, by direct binding to the mRNAs. The FliW protein can directly bind and titrate CsrA activity and in-turn affects CsrA-mediated post-transcriptional regulation of flagellar genes. FliW can also bind to the FlaA protein, which releases FliW-mediated sequestration of CsrA. The abundant *flaA* mRNA is the main target of CsrA translational repression but can also act as a regulatory sponge and titrate CsrA activity together with the main CsrA antagonist FliW. Furthermore, *flaA* mRNA localizes to the cell poles of elongating cells. Polar localization of *flaA* mRNA itself is dependent on its translation, which is controlled by the CsrA-FliW regulatory network.

Using genome-wide RIP-seq, we have identified direct RNA targets of the translational regulator CsrA in a bacterium that lacks the canonical antagonizing sRNAs. Our study revealed the major flagellin mRNA is both the main CsrA target and a dual-function mRNA, which can titrate CsrA activity together with the FliW protein, the main CsrA antagonist (Fig. 9). Compared with microarray-based transcriptome analyses of *csrA* loss-of-function strains^31^,^32^, which might reveal indirect effects or miss targets because of a lack of changes in target mRNA levels despite translational repression, a coIP approach facilitates the identification of direct targets and binding sites. Sanger sequencing of cDNAs from an RsmA-coIP identified six target mRNAs in *P. aeruginosa*^32^. RNA-seq of a CsrA-coIP in *E. coli* revealed 721 co-purified transcripts^33^, and *in vivo* ultraviolet crosslinking combined with RNA-seq (CLIP-seq) revealed 467 potential CsrA-binding sites in *Salmonella typhimurium*, including binding sites in many virulence mRNAs^34^. In our RIP-seq approach, we used untagged WT strains as a negative control to allow for elimination of non-specifically bound transcripts. Our peak-detection tool confirmed the high specificity of this approach, as it detected an ‘ANGGA' sequence in 324/328 targets, which resembles the CsrA consensus-motif determined by *in-vitro* selection^25^. Besides canonical binding to 5′UTRs or early codons^5^,^35^, our coIP also revealed CsrA binding within coding regions or between genes in polycistrons to mediate discoordinate operon regulation.

Our coIP approach revealed many mRNAs of flagellar genes as direct CsrA targets. The motility defect of Δ*csrA* suggests that tight regulation of flagellar genes by CsrA, and especially of the major flagellin FlaA, is required for proper motility. Balancing CsrA activity through the antagonizing protein FliW also appears crucial for flagellar assembly, as we observed that a *C. jejuni* NCTC11168 Δ*fliW* mutant expresses short flagella, as also reported in other strains^27^,^36^, and is defective for autoagglutination and motility in both *B. subtilis* and *C. jejuni*^9^,^26^. Although CsrA impacts motility by directly controlling flagellin expression in *C. jejuni*, *B. subtilis* and *Borrelia*, the strong motility defect of an *E. coli csrA* mutant^37^ is due to a requirement of CsrA for stabilization of the mRNA encoding the master regulator FlhDC^38^. The flagellum also plays an essential, multi-factorial role in *C. jejuni* colonization and pathogenesis, including secretion of Cia/Fed effectors^28^,^39^, and is required for proper cell division^40^. Future studies might reveal CsrA-affected phenotypes beyond motility.

Instead of CsrA-activity control by antagonizing sRNAs^5^, we demonstrated that the *flaA* mRNA itself can titrate CsrA. This represents a new mode of CsrA activity control by a target mRNA-derived antagonist. The *flaA* leader has higher affinity for CsrA compared to other flagellar targets. It has two GGA motifs in adjacent hexaloops, resembling high-affinity CANGGANG-containing apical hexaloop structures targeted by CsrA/RsmE^25^,^41^. The 21-nt spacing between the *flaA* GGA motifs is close to the 18-nt optimal intersite distance for binding of a CsrA dimer^42^. Whereas *flaA* mRNA probably only binds one CsrA dimer, multiple RsmE dimers are cooperatively assembled on RsmZ sRNA^8^,^41^. CsrA titration by a 5′UTR has recently been shown to mediate hierarchical control of fimbriae expression in *Salmonella typhimurium*^43^. The *fimAICDHF* mRNA leader, which in contrast to *flaA* mRNA is not itself a CsrA target, cooperates with the CsrB/C sRNAs to antagonize CsrA-mediated activation of plasmid-encoded fimbriae. Small RNAs other than CsrB/C can also sequester CsrA in addition to functioning as antisense RNAs^44^. Global approaches such as RIP-seq are ideally suited to identify additional antagonizing sRNAs or members of the emerging class of dual-function, cross-regulating mRNAs^2^,^3^.

Analysis of *flaA* mRNA expression in single bacteria using RNA-FISH showed that this transcript localizes to the poles of shorter, and presumably elongating, cells. As a new flagellum is synthesized after each cell division at the new pole of the amphitrichous *C. jejuni*, polar *flaA* mRNA localization might facilitate this process. This temporal and spatial modulation of *flaA* mRNA expression might also affect CsrA-mediated regulation of other flagellar genes through mediating varying levels of this CsrA RNA antagonist. Mutations that either abolish or maintain translation showed *flaA* translation is required for its polar localization. Bacterial mRNA localization has only recently been described and unlike eukaryotes the underlying mechanisms and regulation of this process are poorly understood^45^,^46^. Besides co-translational targeting of mRNAs to the required sites of their encoded products, translation-independent mechanisms of RNA localization have also been described^20^,^21^, including spatial expression according to chromosome organization. We observed that a *flaA* mRNA variant with a premature stop-codon mutation at the 101^st^ codon partially localizes, suggesting a role of the N-terminus in directing the nascent peptide along with the mRNA to the secretion apparatus. Little is known how flagellar substrates are selected for secretion, as they do not share a secretion-signal sequence or cleavable signal peptide. N-terminal domains are required for secretion of flagellar proteins in diverse bacteria, including *C. jejuni*^36^, and both 5′UTR and N-terminal peptide secretion signals have been shown to contribute to secretion efficiency^47^. In addition, flagellar chaperones play a role in regulating the coupling of translation to secretion of flagellar substrates^48^. In *Yersinia*, *cis*-encoded RNA-localization elements in the early coding region are required for secretion of effector proteins by type III secretion systems^49^. Future studies will identify and clarify the role of elements, either in the protein N-terminus or the mRNA 5′UTR, as well as potential interaction partners that are crucial for directing the peptide and/or mRNA to the cell poles and secretion apparatus. Besides the requirement of *flaA* translation for localization, other factors such as the *flaA* genomic location or the transcriptional complex might also contribute to polar *flaA* mRNA localization.

Our study revealed an unexpected function for the CsrA-FliW network in spatial and temporal gene-expression control, and specifically FliW affects translation-dependent polar localization of the flagellin mRNA by antagonizing CsrA-mediated translational repression. The limited CsrA activity in WT cells under standard growth conditions, because of sequestration by the FliW protein antagonist, probably allows sufficient translation of *flaA* mRNA for its polar localization. Strong CsrA-mediated translation repression of *flaA* upon *fliW* deletion is probably responsible for the diffuse *flaA* localization in the Δ*fliW* mutant. CsrA binding might mediate storage of translationally inactive *flaA* mRNA until synthesis of FlaA is required or proper localization is achieved, similar to mRNP granules in eukaryotes^50^. Future studies will show whether other flagellar mRNAs also polarly localize and if the CsrA-FliW regulatory network also impacts their localization. CsrA-mediated regulation of mRNA localization might also occur in *B. subtilis* and *B. burgdorferi,* where CsrA overexpression represses the major flagellin^51^,^52^,^53^. An analogous system might have also evolved in the Alphaproteobacterium *Caulobacter crescentus,* which encodes two proteins with opposing activities on flagellin regulation, FlaF and FlbT, whereby FlbT post-transcriptionally regulates flagellin expression^54^.

Our identification of *C. jejuni* CsrA titration by FliW indicates that CsrA-activity control by a protein antagonist, a mechanism first identified in the Gram-positive *B. subtilis*^9^, is more widespread than previously appreciated. Besides the post-transcriptional effect of FliW on *flaA* and other flagellar genes by antagonizing CsrA, deletion of *fliW* directly or indirectly increases *flaA* transcription. Transcription of *hag* is also twofold upregulated in *B. subtilis* upon *fliW* deletion^9^,^55^. Although FliW appears to be the main CsrA antagonist, its synergistic interplay with the *flaA* mRNA antagonist affects other flagellar genes showed that RNA-based regulation can also impact CsrA activity in this type of Csr network. Gammaproteobacterial genomes encode CsrA^56^ as well as the antagonizing sRNAs^5^ and an anti-correlation between the presence of the CsrB/C sRNAs and FliW has been observed^57^. As the *csrA* gene is located next to a tRNA cluster in *E. coli*, this strongly suggests the pleiotropic function of CsrA in Gammaproteobacteria might have been horizontally acquired, followed by evolution of the antagonizing sRNAs. Thus, the conserved or possibly more ancient function of the CsrA-FliW system might be to mediate temporal and spatial control of proper flagellum assembly. During our conservation analysis we observed that certain non-flagellated *Campylobacter* species, such as *C. hominis*, *C. gracilis* and *C. ureolyticus,* lack *csrA* and *fliW* homologues, further supporting their conserved function in flagellar regulation. Further studies are required to unravel the full complexity of the CsrA-FliW regulatory network and its impact on RNA localization.

## Methods

### Bacterial strains, oligonucleotides and plasmids

All *C. jejuni* and *E. coli* strains used in this study are listed in Supplementary Table 3 and DNA oligonucleotides in Supplementary Table 4, respectively. Plasmids are summarized in Supplementary Table 5.

### Bacterial growth conditions

*C. jejuni* strains were routinely grown on Müller-Hinton agar plates or with shaking in Brucella broth (BB), both supplemented with 10 μg ml^−1^ vancomycin, at 37 °C under microaerobic (10% CO_2_, 5% O_2_) conditions as described previously^14^. The agar was further supplemented with marker-selective antibiotics (20 μg ml^−1^ chloramphenicol, 50 μg ml^−1^ kanamycin, 20 μg ml^−1^ gentamicin or 250 μg ml^−1^ hygromycin B) where appropriate. *E. coli* strains were grown aerobically at 37 °C in Luria-Bertani (LB) medium supplemented with appropriate antibiotics. For induction of arabinose-inducible pBAD promoter, 0.001% (+) or 0.003% (++) L-arabinose was added to LB media.

### Construction of bacterial mutant strains

All *C. jejuni* mutant strains (deletion, chromosomal 3xFLAG-tagging, chromosomal point mutations) were constructed using double-crossover homologous recombination. Cloning strategies and the generation of constructs are described in detail in the Methods and Supplementary Methods. Oligonucleotides used to amplify regions of upstream/downstream homology and resistance cassettes for homologous recombination, as well as recipient strains and oligonucleotides for validation of mutant strains by colony PCR, are listed in Supplementary Table 6 for each generated strain. Introduction of PCR products with 500 bp homologous ends or genomic DNA with mutant constructs into *C. jejuni* was performed by electroporation or natural transformation, respectively, as described previously^14^.

### Construction of 3xFLAG epitope-tagged proteins in *C. jejuni*

*C. jejuni* genes were chromosomally tagged at their C-terminus either by cloning of constructs for C-terminal epitope tagging on plasmids or by construction of 3xFLAG constructs by overlap PCR.

*Tagging of proteins using PCR products amplified from plasmid constructs*. The CsrA, FlaA, FlgI and FlaB proteins were fused to a 3xFLAG epitope at their C-termini by cloning regions encoding ∼500 bp of their C-terminal coding region (C-term) and ∼500 bp downstream of the stop codon (DN) into plasmid pGG1 to flank a 3xFLAG tag and *aphA-3* Kan^R^ cassette. Afterwards, the 3xFLAG-tag constructs were amplified by PCR and introduced into the chromosome of *C. jejuni* strains by electroporation and double-crossover homologous recombination. An example of this plasmid cloning strategy is described for *csrA.* Approximately 500 bp of the region downstream of *csrA* was amplified from genomic DNA (gDNA) with primers CSO-0173/-0174. These primers included *Xba*I and *Eco*RI sites, respectively. Following cleanup, the PCR product was digested with *Eco*RI and *Xba*I and ligated into a similarly digested pGG1 backbone, generated by inverse PCR with primers CSO-0074/-0075, to create pGD2-1. The plasmid was verified by colony PCR with primers JVO-0054/CSO-0173 and the sequence was verified using JVO-0054. Next, the backbone of this plasmid, including the *csrA* ‘DN' region, was amplified by PCR with primers CSO-0073 (*Xho*I) and JVO-5142 (blunt). The C-terminal coding region of *csrA* (∼500 bp) without the stop codon was amplified with primers CSO-0171/-0172 from NCTC11168 WT gDNA. The sense primer (CSO-0172) included an *Xho*I site, whereas the antisense primer (CSO-0171) contained a 5′-phosphate. Both the plasmid backbone with the ‘DN' insert and the C-term insert were digested with *Xho*I and ligated to create plasmid pGD4-1. Integration of the PCR product was confirmed by colony PCR using primers CSO-0172/-0023 and the plasmid was validated by sequencing using CSO-0023. The entire integration cassette was then amplified with Phusion High-Fidelity DNA polymerase (NEB) using primers CSO-0172/-0173 and electroporated into *C. jejuni* and selected on kanamycin plates. Mutants were confirmed by colony PCR with primers CSO-0196/-0023 and western blot analysis with an anti-FLAG antibody.

*3xFLAG tagging of proteins by overlap PCR*. Construction of a C-terminal 3xFLAG translational fusion at its native locus was performed by overlap PCR for *flaG* as described in Supplementary Methods for gene deletions, but with the following modifications. The final overlap PCR product contained ∼500 bp of the C-terminal coding region of *flaG* minus the stop codon (C-term) and ∼500 bp downstream of *flaG* (DN) for homologous recombination. These regions flanked an in-frame 3xFLAG tag and stop codon followed by an *aphA-3* Kan^R^ cassette. For example, for tagging *flaG,* the 3xFLAG tag and Kan^R^ cassette was amplified from plasmid pGG1 with primers JVO-5142 and HPK2. The ‘C-term' region of *flaG* was amplified using primers CSO-1002/-1098, where CSO-1098 is antisense and contains region of complementarity at its 5′ end to the 3xFLAG tag/JVO-5142, from NCTC11168 gDNA. The ‘DN' region was amplified using primers CSO-1099/-1003, where CSO-1099 is sense to *flaG* DN and contains a region of complementarity to the 3′ end of the Kan^R^ cassette/primer HPK2. If the coding region of the target gene contained sequences required for expression of a downstream ORF (that is, SD sequence or codons), these sequences were included in the ‘DN' amplicon. The three PCR products were then used for overlap PCR with primers CSO-1002/-1003, and the resulting amplicon was electroporated into *C. jejuni*, followed by selection of positive clones on kanamycin plates. Mutants were checked by colony PCR with primers CSO-1005/HPK2 and western blot analysis with an anti-FLAG antibody.

### Introducing chromosomal point mutations into the *flaA* leader

To introduce point mutations into the 5′UTR of *flaA* at the native locus, a 1,100-bp region around the *flaA* promoter was amplified using oligos CSO-0752/-0753. These primers introduced *Xho*I and *Xba*I sites, respectively, into the resulting PCR product. After *Xho*I and *Xba*I digestion, the product was then ligated into a similarly digested plasmid pJV752-1, resulting in plasmid pGD70-5. Plasmid pGD70-5 was checked by colony PCR using primers pZE-A/CSO-0753 and sequencing with pZE-A. Next, plasmid pGD70-5 was amplified by inverse PCR using primers CSO-0754/-0755, thereby introducing *Nde*I and *Bam*HI restriction sites 40 nt upstream of the *flaA* transcriptional start site (TSS). An *aac*(3)-IV gentamicin resistance cassette with its own promoter and terminator was amplified using CSO-0483/-0576 and introduced into PCR-amplified pGD70-5 in the reverse orientation to *flaA,* just upstream of its promoter, using the *Nde*I/*Bam*HI restriction sites, resulting in plasmid pGD76-1. Plasmid pGD76-1 was checked by colony PCR using primers CSO-0576/-0753 and sequencing with CSO-0753.

Point mutations were then introduced into the *flaA* 5′UTR by inverse PCR on pGD76-1 using complementary oligos harbouring the desired mutation, followed by *Dpn*I digestion and transformation of the resulting purified PCR product into *E. coli* TOP10. For introduction of the *flaA* M1 mutation (GGA>AAA in stem-loop SL1 of the *flaA* leader), oligonucleotides CSO-1114/-1115 were used for PCR on pGD76-1. The mutation was confirmed in the resulting plasmid pGD92-1 by sequencing with CSO-0753. Similarly, the *flaA* M2 (GGA>UGA in stem-loop SL1 of the *flaA* leader), M3 (GGA>GGG in stem-loop SL2 of the *flaA* leader), X1 start codon (AUG>AAG), X2 start codon (AUG>AUU), X3 start codon (AUG>GUG), X4 3^rd^ codon (UUU>UAG), X5 3^rd^ codon (UUU>UUC) and X6 101^st^ codon (CAA>UAA) mutations were introduced using primer pairs CSO-0757/-0758, CSO-1116/-1117, CSO-2019/-2020, CSO-2827/-2828, CSO-2825/-2826, CSO-2829/-2830, CSO-2831/-2832 and CSO-2833/-2834, respectively, resulting in plasmids pGD77-1, pGD93-1, pGD114-2, pGD205-1, pGD204-1, pGD206-1, pGD207-1 and pGD208-1, respectively. For combination of the *flaA* M2 and M3 mutations, a similar mutagenesis approach was performed based on PCR amplification of the M2 plasmid pGD77-1 using oligonucleotides CSO-1116/-1117, resulting in pGD95-1 harbouring both the mutations. To introduce the *flaA* 5′UTR mutations into *C. jejuni*, a PCR product covering the homologous ends and the gentamicin resistance cassette was amplified from the respective WT (pGD76-1) or mutant plasmids using CSO-0752/-0850 and electroporated into *C. jejuni* as described above. To confirm introduction of point mutation in *C. jejuni,* colony PCR was performed using CSO-0576/-0753 and sequencing with CSO-0850.

### Construction of *E. coli* mutants

The *E. coli* Δ*pgaA* and Δ*pgaA* Δ*csrA* deletion strains were constructed in the TOP10 background using the λ Red protocol^58^. Briefly, a kanamycin resistance gene, amplified from plasmid pKD4 using primers CSO-0652/-0653, was used to replace the entire *pgaA* ORF excluding the start and stop codon. The mutant strain was verified by colony PCR using the primer pairs CSO-0654/-0653 and CSO-0652/-0655. After verification, helper plasmid pCP20 containing FLP recombinase was introduced to remove the kanamycin resistance marker^58^. The helper plasmid, which is temperature-sensitive and carries an ampicillin resistance marker, was then cured by recovering colonies at 37 °C and confirming ampicillin sensitivity, resulting in strain CSS-0556. Similarly, the ORF of the *csrA* gene excluding the start and stop codon was then replaced by the kanamycin resistance marker (amplified using CSO-0611/-0612) in the Δ*pgaA* strain resulting in strain CSS-0557, harbouring both *pgaA* and *csrA* deletions. The *csrA* deletion was verified by colony PCR using primer pairs CSO-0639/-0612 and CSO-0611/-0640.

### RIP-seq of *C. jejuni* CsrA-3xFLAG

coIP combined with RNA-seq (RIP-seq) to identify direct RNA-binding partners of CsrA-3xFLAG in *C. jejuni* was performed as previously described^18^,^59^ with minor modifications.

*CoIP of RNA with CsrA-3xFLAG*. CoIP of chromosomally epitope-tagged *C. jejuni* CsrA with an anti-FLAG antibody and Protein A-Sepharose beads was performed from lysates of *C. jejuni* NCTC11168 and 81-176 WT (control) and isogenic *csrA*-3xFLAG strains grown in 100 ml (50 ml × 2 flasks) BB containing 10 μg ml^−1^ vancomycin to mid-exponential phase (OD_600_=0.6) at 37 °C as described previously for *H. pylori*^18^. Cells were harvested by centrifugation at 6,000*g* for 15 min at 4 °C. Afterwards, cell pellets were resuspended in 1 ml Buffer A (20 mM Tris-HCl, pH 8.0, 150 mM KCl, 1 mM MgCl_2_, 1 mM dithiothreitol (DTT)) and subsequently centrifuged (3 min, 11,000*g*, 4 °C). The pellets were shock-frozen in liquid nitrogen and stored at −80 °C. Frozen pellets were thawed on ice and resuspended in 0.8 ml Buffer A. An equal volume of glass beads was then added to the cell suspension. Cells were then lysed using a Retsch MM40 ball mill (30 s^−1^, 10 min) in pre-cooled blocks (4 °C) and centrifuged for 2 min at 15,200*g*, 4 °C. The supernatant was transferred to a new tube, and an additional 0.4 ml of Buffer A was added to the remaining un-lysed cells with beads. Lysis of the remaining cells was achieved by a second round of lysis at 30 s^−1^ for 5 min. Centrifugation was repeated and this second supernatant was combined with the first one. The combined supernatant was centrifuged again for 30 min at 15,200*g*, 4 °C for clarification and the resulting supernatant (lysate fraction) was transferred to a new tube. The lysate was incubated with 35 μl anti-FLAG antibody (Monoclonal ANTI-FLAG M2, Sigma, #F1804) for 30 min at 4 °C on a rocker. Next, 75 μl of Protein A-Sepharose (Sigma, #P6649), prewashed with Buffer A, was added and the mixture was rocked for another 30 min at 4 °C. After centrifugation at 15,200*g* for 1 min, the supernatant was removed. Pelleted beads were washed five times with 0.5 ml Buffer A. Finally, 500 μl Buffer A was added to the beads and RNA and proteins were separated by phenol-chloroform-isoamyl alcohol extraction and precipitated as described previously^18^. From each coIP, 700–1,000 ng of RNA was recovered. 100 μl of 1 × protein loading buffer (62.5 mM Tris-HCl, pH 6.8, 100 mM DTT, 10% (v/v) glycerol, 2% (w/v) SDS, 0.01% (w/v) bromophenol blue) was added to the final protein sample precipitated along with beads. This sample was termed the coIP sample. For verification of a successful coIP, protein samples equivalent to 1.0 OD_600_ of cells were obtained during different stages of the coIP (culture, lysate, supernatant, wash and coIP (beads)) for further western blot analysis. One hundred microlitres of 1 × protein loading buffer was added to the protein samples and boiled for 8 min. Protein sample corresponding to an OD_600_ of 0.1 or 0.15 (culture, lysate, supernatant and wash fraction) and 10 or 5 (for proteins precipitated from beads) were used for western blot analysis.

*RIP-Seq cDNA library preparation*. Residual gDNA was removed from the coIP RNA samples isolated from the control (WT) and CsrA-3xFLAG coIPs of the two strains *C. jejuni* NCTC11168 and 81-176 using DNase I treatment. cDNA libraries for Illumina sequencing were constructed by vertis Biotechnologie AG (http://www.vertis-biotech.com) in a strand-specific manner as described previously^14^. In brief, equal amounts of RNA samples were poly(A)-tailed using poly(A) polymerase. Then, 5′-triphosphates were removed using tobacco acid pyrophosphatase, and an RNA adapter was then ligated to the resulting 5′-monophosphate. First-strand cDNA was synthesized with an oligo(dT)-adapter primer using M-MLV reverse transcriptase. In a PCR-based amplification step, using a high-fidelity DNA polymerase, the cDNA concentration was increased to 20–30 ng μl^−1^. For all libraries, the Agencourt AMPure XP kit (Beckman Coulter Genomics) was used to purify the DNA, which was subsequently analysed by capillary electrophoresis.

A library-specific barcode for multiplex sequencing was included as part of a 3′-sequencing adapter. The following adapter sequences flank the cDNA inserts:

TrueSeq_Sense_primer

5′-AATGATACGGCGACCACCGAGATCTACACTC TTTCCCTACACGAC GCTCTTCCGATCT-3′

TrueSeq_Antisense_NNNNNN_primer (NNNNNN=6nt barcode for multiplexing)

5′-CAAGCAGAAGACGGCATACGAGAT-NNNNNN-GTGACTGGAGTTCAGACGTGTGCTCTTCCGATC(dT25)-3′.

The samples were sequenced on an Illumina HiSeq instrument with 100 cycles in single-read mode. The resulting read numbers are listed in Supplementary Table 1.

### Analysis of deep sequencing data

To assure high sequence quality, the Illumina reads in FASTQ format were trimmed with a cutoff phred score of 20 by the programme fastq_quality_trimmer from FASTX toolkit version 0.0.13. After trimming, poly(A)-tail sequences were removed and a size filtering step was applied in which sequences shorter than 12 nt were eliminated. The collections of remaining reads were mapped to the *C. jejuni* NCTC11168 (NCBI Acc.-No: NC_002163.1) and 81-176 (NCBI Acc.-No: NC_008770.1, NC_008787.1, NC_008790.1) genomes using *segemehl*^60^ with an accuracy cutoff of 95%. Mapping statistics are listed in Supplementary Table 1. Coverage plots representing the numbers of mapped reads per nucleotide were generated. Reads that mapped to multiple locations contributed a fraction to the coverage value. For example, reads mapping to three positions contributed only one-third to the coverage values. Each graph was normalized to the number of reads that could be mapped from the respective library. To restore the original data range, each graph was then multiplied by the minimum number of mapped reads calculated over all libraries.

The overlap of sequenced cDNA reads to annotations was assessed for each library by counting all reads overlapping selected annotations on the sense strand. These annotations consist of strain-specific NCBI gene annotations complemented with annotations of previously determined 5′UTRs and small RNAs^14^. Each read with a minimum overlap of 10 nt was counted with a value based on the number of locations where the read was mapped. If the read overlapped more than one annotation, the value was divided by the number of regions and counted separately for each region (for example, one-third for a read mapped to three locations).

### Enrichment analysis of CsrA targets

Enrichment of transcripts in the CsrA-3xFLAG coIP versus control coIP libraries was determined based on mapped cDNA read counts for annotations provided in NC_002163.gff (NCBI) for NCTC11168 using GFOLD version 1.0.9 (ref. 61) but with manually defined normalization constants based on the number of reads that could be mapped to the respective libraries. For determination of genes enriched in the CsrA-3xFLAG-tagged library, log2 fold changes (FCs) rather than GFOLD values were used. Similar analysis was done for strains 81-176 using annotations provided in NC_008787.gff (chromosome), NC_008770.gff (pVir plasmid) and NC_008790.gff (pTet plasmid).

### Peak detection and CsrA-binding motif analyses

To automatically define CsrA-bound RNA regions or peaks from the CsrA-3xFLAG coIP data sets, an in-house tool ‘sliding_window_peak_calling_script' was developed based on a sliding window approach. A detailed description of the tool will be described elsewhere. The script has been deposited at Zenodo (https://zenodo.org/record/49292) under DOI 10.5281/zenodo.49292 (http://dx.doi.org/10.5281/zenodo.49292). The script is written in Python 3 and requires installation of the Python 3 packages *numpy* and *scipy* for execution.

In brief, the ‘sliding_window_peak_calling_script' software uses normalized wiggle files of the CsrA-3xFLAG and control coIP libraries as input to determine sites showing a continuous enrichment of the CsrA-3xFLAG-tagged library compared with the control. The identification of enriched regions is based on four parameters: a minimum required fold change (FC) for the enrichment, a factor multiplied by the 90th percentile of the wiggle graph, which reflects the minimum required expression (MRE) in the tagged library, a window size in nt (WS), for which the previous two values are calculated in a sliding window approach, and a nucleotide step size (SS), which defines the steps in which the window is moved along the genomic axis. All consecutive windows that fulfill the enrichment requirements are assembled into a single peak region. The peak detection is performed separately for the forward and reverse strand of each replicon. For the CsrA-3xFLAG coIP data set, the following parameters were used: FC=5, MRE=3, WS=25 and SS=5.

For the prediction of consensus motifs based on the peak sequences, MEME^24^ and CMfinder 0.2.1 (ref. 62) were used. For MEME^24^ predictions, the following settings were applied: Search 0 or 1 motif of length 4–7 bp per sequence in the given strand only. To search for the presence of a structural motif, CMfinder 0.2.1 (ref. 62) was run on the enriched peak sequences with default parameters except for allowing a minimum single stem loop candidate length of 20 nt. The top-ranked motif incorporated 276 of the 328 sequences and was visualized by R2R^63^.

### Functional classes enrichment analysis

To check for overrepresentation of functional classes of CsrA-bound genes, we considered genes with at least fivefold enrichment in their 5′UTR and/or coding sequence in the CsrA-3xFLAG coIP library (versus control) as CsrA-bound and the remaining genes as unbound. We applied an existing functional classification^64^ of genes from strain NCTC11168 to determine statistically enriched functional classes. Because a similar classification was not available for strain 81-176, a table with orthologue mappings between the two strains was downloaded from OrtholugeDB^65^ and used to assign the NCTC11168 functional classes to their respective 81-176 counterparts. Genes in our annotation lists without an existing functional classification in NCTC11168 or without an orthologue match were assigned to class 5.I, defined as ‘Unknown', in the original classification scheme. Genes encoded on the pVir and pTet plasmids of strain 81-176 were assigned to new pVir and pTet classes, respectively. Functional overrepresentation was analysed for each functional class via a two-sided Fisher's exact test followed by multiple-testing correction using the Benjamini–Hochberg method. An adjusted *P*-value of 0.05 was selected as significance threshold for functional overrepresentation.

### Protein–protein coIP

The FliW and CsrA protein–protein coIP was performed exactly as described for the RIP-seq coIP protocol (see above) until the step where beads were washed five times with Buffer A. After washing, the beads were suspended in 200 μl of 1 × protein loading buffer (62.5 mM Tris-HCl, pH 6.8, 100 mM DTT, 10% (v/v) glycerol, 2% (w/v) SDS, 0.01% (w/v) bromophenol blue) and boiled for 8 min. Lysate samples corresponding to an OD_600_ of 0.05 and 2 (for proteins precipitated from beads) were used for western blot analysis.

### SDS–PAGE and immunoblotting

Protein analyses were performed on cells collected from *C. jejuni* in mid-exponential phase (OD_600_ 0.5–0.6) or *E. coli* cultures in late-exponential phase (OD_600_ 1.0–1.5). Cells were collected by centrifugation at 11,000*g* for 3 min. Cell pellets were resuspended in 100 μl of 1 × protein loading buffer (62.5 mM Tris-HCl, pH 6.8, 100 mM DTT, 10% (v/v) glycerol, 2% (w/v) SDS, 0.01% (w/v) bromophenol blue) and boiled for 8 min. For western blot analysis, samples corresponding to an OD_600_ of 0.02 to 0.1 were separated by 12, 15 or 18% (v/v) SDS-polyacrylamide (PAA) gels and transferred to a nitrocellulose membrane by semidry blotting. Membranes were blocked for 1 h with 10% (w/v) milk powder/TBS-T (Tris-buffered saline-Tween-20) and incubated overnight with primary antibody at 4 °C. Membranes were then washed with TBS-T, followed by 1 h incubation with secondary antibody. After washing, the blot was developed using enhanced chemiluminescence-reagent. GFP-, FLAG- and Strep-tagged proteins of interest were detected with monoclonal anti-GFP (1:1,000 in 3% BSA/TBS-T; Roche, #11814460001), monoclonal anti-FLAG (1:1,000 in 3% BSA/TBS-T; Sigma-Aldrich, #F1804-1MG) or monoclonal anti-Strep (1:10,000 in 3% BSA/TBS-T; IBA GmbH, #2-1507-001) primary antibodies and anti-mouse IgG (1:10,000 in 3% BSA/TBS-T; GE-Healthcare, #RPN4201) secondary antibody. mCherry-tagged proteins were detected using a polyclonal anti-mCherry (1:4,000 in 3% BSA/TBS-T; Acris, #AB0040-20) primary antibody and an anti-goat (1:10,000 in 3% BSA/TBS-T; Santa Cruz Biotechnology, #sc2020) secondary antibody. A monoclonal antibody specific for GroEL (1:10,000 in 3% BSA/TBS-T; Sigma-Aldrich, # G6532-5ML) and an anti-rabbit IgG (1:10,000 in 3% BSA/TBS-T; GE-Healthcare, #RPN4301) secondary antibody were used as a loading control. Images of full blots that were cropped in main Figures are shown in Supplementary Fig. 19.

### Validation of CsrA targets with a GFP reporter system

Validation of CsrA targets was performed using a heterologous *E. coli* system previously developed for validation of sRNA–mRNA interactions^23^. Selected candidate *C. jejuni* CsrA target sequences from the coIP were cloned as translational fusions to GFP or FLAG in plasmids pXG-10 or pXG-30 as listed in Supplementary Tables 5 and 7. Levels of FLAG or GFP translational fusions were then determined by western blotting or FACS in *E. coli* Δ*pgaA*, Δ*pgaA* Δ*csrA* and a Δ*pgaA* Δ*csrA* strain harbouring plasmid pGD72-3 with *C. jejuni* CsrA-Strep under the control of an arabinose-inducible promoter.

### Flow cytometric analysis

For FACS analysis of GFP reporter fluorescence in *E. coli*, cells corresponding to 1 OD_600_ were collected from LB cultures in log phase and resuspended in 0.25 ml PBS. Cells were then fixed for 10 min with 0.25 ml of 4% paraformaldehyde, collected by centrifugation and washed twice with 0.5 ml PBS before final resuspension in 0.5 ml PBS. A 1/100 dilution of the fixed sample in PBS was used for measurement. Measurements (50 000 counts per sample) were performed on a BD FACSCalibur machine and analysed using FlowJo (V10).

### Purification of *C. jejuni* CsrA

Recombinant, C-terminal Strep-tagged *C. jejuni* CsrA (Cj1103) was overexpressed and purified from *E. coli* TOP10 Δ*pgaA*/Δ*csrA* using Strep-Tactin Sepharose (IBA GmbH, #2-1202-001). Primers and plasmids used for cloning are listed in Supplementary Tables 4 and 5 The *csrA* gene, including its SD sequence, was fused to a C-terminal Strep-tag in the arabinose-inducible plasmid pBAD/Myc-His A (Invitrogen) for overexpression and affinity purification. The *csrA*-coding region and SD were amplified from *C. jejuni* NCTC11168 genomic DNA using primers CSO-0746/-0747, and the pBAD/Myc-His A plasmid was amplified by inverse PCR with JVO-0900/-0901 as previously described^66^. CSO-0747 and JVO-0901 introduce an *Xba*I site to the insert and vector, respectively, whereas CSO-0746 has a 5′-phosphate to facilitate blunt-end ligation. *Xba*I-digested insert and vector were then ligated, resulting in pGD68-1. Plasmid pGD68-1 was checked by colony PCR using primers pBAD-FW/CSO-0747 and sequencing with pBAD-FW. A Strep-tag (WSHPQFEK) was then added at the C-terminus of *csrA* by inverse PCR using oligonucleotides CSO-0852/-0853, resulting in plasmid pGD72-3. Plasmid pGD72-3 was checked by sequencing with pBAD-FW. Plasmid pGD72-3 was then introduced into an *E. coli* TOP10 Δ*pgaA*/Δ*csrA* deletion strain resulting in strain CSS-0931. CSS-0931 was grown in 500 ml LB broth with 100 μg ml^−1^ of ampicillin at 37 °C and shaking at 220 r.p.m. to an OD_600_ of 0.3, at which time L-arabinose was added to a final concentration of 0.01%. The culture was then incubated for an additional 8 h at 18 °C. Cells were harvested by centrifugation at 7,000*g* for 30 min at 4 °C. The pellet was resuspended in 5 ml of Buffer W (IBA GmbH, #2-1003-100). The rest of the protocol was followed as per the manufacturer's instructions using 1 ml Gravity flow Strep-Tactin Sepharose. After washing steps, the CsrA-Strep protein was finally eluted using Buffer E (IBA GmbH, #2-1000-025) in three successive steps (E1: 0.8 ml, E2: 1.4 ml and E3: 0.8 ml). The majority of CsrA-Strep was concentrated in the E2 fraction. Concentration was quantified using Roti-Quant (Carl ROTH, #K015.3), and the protein was stored at −20 °C in 50 μl aliquots.

### RNA isolation

Bacteria were grown to the indicated growth phase and culture volume corresponding to a total amount of 4 OD_600_ was harvested and mixed with 0.2 volumes of stop-mix (95% ethanol and 5% phenol, vol/vol). The samples were snap-frozen in liquid nitrogen and stored at −80 °C until RNA extraction. Frozen samples were thawed on ice and centrifuged at 4 °C to collect cell pellets. Cell pellets were lysed by resuspension in 600 μl of a solution containing 0.5 mg ml^−1^ lysozyme in TE buffer (pH 8.0) and 60 μl of 10% SDS. The samples were incubated for 1–2 min at 65 °C to ensure lysis. Afterwards, total RNA was extracted using the hot-phenol method as described previously^13^,^14^.

### Northern blot analysis

For northern blot analysis, 5–10 μg RNA sample was loaded per lane. After separation on 6% PAA gels containing 7 M urea, RNA was transferred to Hybond-XL membranes (GE-Healthcare) by electroblotting. After blotting, the RNA was ultraviolet cross-linked to the membrane and hybridized with γ32P-ATP end-labelled DNA oligonucleotides (Supplementary Table 4).

### Rifampicin RNA stability assays

To determine the stability of *flaA* mRNA in *C. jejuni* NCTC11168 WT, Δ*csrA*, Δ*fliW* and Δ*csrA* Δ*fliW* strains, cells were grown to an OD_600_ of 0.45 (mid-log phase) and treated with rifampicin to a final concentration 500 μg ml^−1^. Samples were harvested for RNA isolation at indicated time points following rifampicin addition (0, 4, 8, 16 and 32 min) as described above. After RNA isolation, 10 μg of each RNA sample was used for northern blot analysis as detailed above.

### *In-vitro* T7 transcription and RNA labelling

DNA templates containing the T7 promoter sequence were generated by PCR using oligos and DNA templates listed in Supplementary Table 8. T7 *in-vitro* transcription of RNAs was carried out using the MEGAscript T7 kit (Ambion) and sequences of the resulting T7 transcripts are listed in Supplementary Table 8. *In vitro* transcribed RNAs were quality checked and 5′ end-labelled (γ^32^P) as previously described^66^,^67^.

### Gel mobility shift assays

Gel-shift assays were performed using ∼0.04 pmol 5′-labelled RNA (4 nM final concentration) with increasing amounts of purified *C. jejuni* CsrA in 10 μl reactions. In brief, 5′-radiolabelled RNA (^32^P, 0.04 pmol in 6 μl) was denatured (1 min, 95 °C) and cooled for 5 min on ice. Yeast tRNA (1 μg) and 1 μl of 10 × RNA Structure Buffer (Ambion: 10 mM Tris, pH 7, 100 mM KCl, 10 mM MgCl_2_) was then added to the labelled RNA. CsrA protein (2 μl diluted in 1 × Structure Buffer) was added to the desired final concentrations (0 mM, 10 nM, 20 nM, 50 nM, 100 nM, 200 nM, 500 nM, 1 μM or 2 μM CsrA). Binding reactions were incubated at 37 °C for 15 min. Before loading on a pre-cooled native 6% PAA, 0.5 × TBE gel, samples were mixed with 3 μl native loading buffer (50% (v/v) glycerol, 0.5 × TBE, 0.2% (w/v) bromophenol blue). Gels were run in 0.5 × TBE buffer at 300 V at 4 °C for 3 h. Gels were dried and analysed using a PhosphoImager (FLA-3000 Series, Fuji).

### *In vitro* structure probing assays

*In vitro* structure probing of *flaA* WT and *flaA* M1/M2 leaders with RNase T1 and lead(II) acetate was performed as previously described^68^. For each reaction, 0.1 pmol of a labelled *flaA* leader variant was denatured for 1 min at 95 °C and chilled on ice for 5 min. One microgram yeast tRNA as competitor and 10 × RNA Structure Buffer was added (provided together with RNase T1, Ambion). Unlabelled recombinant *C. jejuni* CsrA protein was then added at 0-, 20-, 50- or 100-fold molar excess. After incubation for 15 min at 37 °C, 2 μl RNase T1 (0.01 U μl^−1^) or 2 μl freshly prepared lead(II)-acetate solution (25 mM) were added and reactions were incubated for 3 min or 90 s, respectively. As a control, ∼0.1 pmol labelled RNA with 100-fold excess CsrA was also prepared without nuclease/lead(II) treatment. The reactions were stopped by addition of 12 μl Gel loading buffer II (#AM8546G, Ambion). For RNase T1 ladders, ∼0.1 pmol labelled RNA was denatured in 1 × Structure Buffer for 1 min at 95 °C and afterwards incubated with 0.1 U μl^−1^ RNase T1 for 5 min. The OH ladder was generated by incubation of ∼0.1 pmol labelled *flaA* WT leader RNA in 1 × alkaline hydrolysis buffer (Ambion) for 5 min at 95 °C. Ladders and samples were then separated on 10% (v/v) PAA/7M urea gels in 1 × TBE buffer. Gels were dried, exposed to a screen and analysed using a PhosphorImager (FLA-3000 Series, Fuji).

### Transmission electron microscopy

*C. jejuni* WT and mutant strains were grown for 14 h on MH plates supplemented with vancomycin (10 μg ml^−1^). Cells were resuspended gently in PBS using a cotton swab and centrifuged at 5,000*g* for 5 min. The cell pellet was resuspended in 2% glutaraldehyde in 0.1 M cacodylate and incubated at 4 °C overnight. The next day, samples were stained with 2% uranyl acetate and imaged using a Zeiss EM10 transmission electron microscope.

### Motility assays

*C. jejuni* strains were inoculated from the appropriate selective MH agar plates into 20 ml BB containing 10 μg ml^−1^ vancomycin and grown microaerobically with shaking at 37 °C to an OD_600_ of ∼0.5. Cells were harvested by centrifugation at 6,500*g* for 5 min and resuspended at an OD_600_ of 0.5 in BB. For each strain, 0.5 μl of bacterial suspension was inoculated into motility soft-agar plates (MH broth+0.4% agar) poured the day before. Plates were incubated right-side-up for ∼24 h microaerobically at 37 °C. Three measurements of each motility halo were made for each inoculation, which were averaged to give the mean swim distance for each strain on a plate. All strains were inoculated together on six replicate plates and the mean swim distance±standard error on these plates was used to the compare motility of each strain.

### Autoagglutination assay

Autoagglutination was determined as described previously^26^. Briefly, strains grown in liquid cultures for motility assays were resuspended in PBS, pH 7.4, to an OD_600_ of 1.0. Two millilitres were placed into three replicate tubes and the OD_600_ was measured. Tubes were incubated at 37 °C microaerobically without shaking, and at indicated time points, 100 μl was carefully removed from the top of the suspension, diluted tenfold in PBS, and the OD_600_ was measured. Measurements were normalized to the optical density of each strain at the zero time point.

### Time-lapse microscopy to monitor cell division

*C. jejuni* Δ*fliA* mutant cells corresponding to an OD_600_ of 0.5 were collected from BB culture in log phase by centrifugation and resuspended in 0.5 ml BB. The cells were further serially diluted 100- and 1,000-fold in BB. Five microlitres of the diluted samples were spotted on a BB-agarose (1%) plate. The plate was incubated under microaerophilic conditions at 37 °C for 10 min. The agarose patch was excised and inverted onto a Petri dish with a glass bottom. Single cells were then monitored over time using several bright-field images in a fluorescence microscope (Leica DMI6000 B) maintained at 37 °C under aerobic conditions.

### RNA FISH

RNA-FISH was performed as previously described^69^ with some modifications. A total amount of cells corresponding to two OD_600_ was collected from BB cultures in mid-log phase (OD_600_=0.4) and resuspended in 0.5 ml PBS. Cells were then fixed for 3 h with 0.5 ml 4% paraformaldehyde at room temperature, collected by centrifugation and washed twice with 0.5 ml PBS before final resuspension in 0.5 ml 70% ethanol. After 10 min, cells were collected by centrifugation and resuspended in 95% ethanol and incubated at room temperature for 1 h. Cells were again collected by centrifugation, completely dried in a laminar flow hood and then washed once with 2 × SSC before final resuspension in 0.5 ml of 2 × SSC containing 10% formamide. Fluorescently labelled DNA oligos (14 Cy5-labelled oligos to detect *flaA* mRNA and one FITC-labelled oligo specific for 16 S rRNA, Sigma, Supplementary Table 4) were then added at a concentration of 10 ng μl^−1^ and incubated at 37 °C overnight. The next day, cells were collected by centrifugation and washed three times for 1 h at 37 °C with 0.5 ml of 2 × SSC containing 10% formamide before final resuspension in 2 × SSC (50–250 μl). Cells were then imaged in a Leica Confocal TCS SP5 II microscope using sequential scanning mode.

### *d*STORM

For super-resolution imaging, *C. jejuni* cells were grown, fixed and labelled using the above-described RNA-FISH protocol (14 Cy5-labelled DNA oligonucleotides to detect *flaA* mRNA and a FITC oligo to label 16S rRNA, Sigma, Supplementary Table 4). Labelled cells were immobilized on poly-D-lysine (Sigma-Aldrich)-coated eight-well chambered cover glasses (Sarstedt). For fluorophore photo switching, a buffer with a pH of 8.3–8.5 was used^30^,^70^ containing 50 mM Tris-HCl (pH 8), 10% glucose, 1% 2-mercaptoethanol (Carl Roth), 3 U ml^−1^ pyranose oxidase (Sigma-Aldrich) and 90 U ml^−1^ catalase (Sigma-Aldrich) in 2 × SSC.

*d*STORM was performed on a wide-field setup for localization microscopy^71^. An optically pumped semiconductor laser (Genesis MX STM-Series, Coherent) with a wavelength of 639 nm (maximum power of 1 W) was used for excitation of Cy5 and a diode laser (iBeam smart Family, TOPTICA Photonics) with a wavelength of 405 nm (maximum power of 120 mW) was used for reactivation of Cy5. Laser beams were cleaned-up by bandpass filters (Semrock/Chroma) and combined by appropriate dichroic mirrors (LaserMUX filters, Semrock). Afterwards they were focused onto the back focal plane of the high numerical oil-immersion objective (Olympus APON 60XO TIRF, numerical aperture 1.49), which is part of an inverted fluorescence microscope (Olympus IX71). To separate the excitation light from the fluorescence light, suitable dichroic beam splitters (Semrock) were placed into the light path before the laser beams enter the objective. Fluorescence light collected by the objective was filtered by appropriate detection filters (Semrock/Chroma) and was detected by an EMCCD camera with 512 × 512 pixels (iXon Ultra 897, Andor Technology). The pixel size in the image was 129 nm px^−1^. Cy5 was excited with the 639-nm laser at a maximum intensity of 4.19 kW cm^−2^. During imaging, the 405-nm laser was switched on to keep up a suitable switching ratio. Its laser power was increased successively to a maximum intensity of 0.04 kW cm^−2^. For every image, 5,000–25,000 frames were taken with an integration time of 15 ms per frame. For every imaged area, additionally a bright-field image was taken to identify single bacteria. Data analysis was performed using rapi*d*STORM open source software^72^.

### Statistical analysis

All data for western, northern blot or FISH analysis are presented as mean±s.e.m. Statistical analysis was carried out using Student's *t*-test. For statistical comparison of two groups, a two-tailed paired Student's *t*-test was used. A value of *P*<0.05 was considered significant and marked with an asterisk (*) as explained in the legends. For FISH analysis, fluorescence data curves from 10 cells from a single image were merged as a single averaged curve after cell length normalization. The data were acquired and normalized over cell length using ImageJ and subsequently the merged average curve was generated using Microsoft Excel.

### Code availability

The ‘sliding_window_peak_calling_script' for identification of CsrA-binding sites based on RIP-seq data has been deposited at Zenodo (https://zenodo.org/record/49292) under DOI: 10.5281/zenodo.49292 (http://dx.doi.org/10.5281/zenodo.49292).

### Data availability

The raw, de-multiplexed reads as well as coverage files of the RIP-seq libraries have been deposited in the NCBI Gene Expression Omnibus^73^ under the accession number GSE58419. The authors declare that all other data supporting the findings of this study are available within the article and its supplementary information files, or from the corresponding author upon request.

## Additional information

**How to cite this article:** Dugar, G. *et al*. The CsrA-FliW network controls polar localization of the dual-function flagellin mRNA in *Campylobacter jejuni*. *Nat. Commun.* 7:11667 doi: 10.1038/ncomms11667 (2016).

## Supplementary Material

Supplementary Information Supplementary Figures 1-19, Supplementary Tables 1-8, Supplementary Methods, Supplementary References

Supplementary Data 1 Potential CsrA targets from *C. jejuni* NCTC11168.

Supplementary Data 2 List of potential CsrA binding sites predicted by a peak detection algorithm for *C. jejuni* strains NCTC11168 and 81-176.

## Figures and Tables

**Table 1:** Enrichment of genes involved in flagellar biosynthesis in the CsrA coIP data.

	***C. jejuni*** **NCTC11168**		***C. jejuni*** **81-176**	
**Enrichment (reads)**	**5′UTR**	**ORF**	**5′UTR**	**ORF**
*Regulation of expression (class 1)*				
** *****rpoN*** **(Cj0670)**	1.5 × (26)	**25 × (1,747)**	−	**8 × (634)**
** ***fliA* (Cj0061c)	−	1.1 × (121)	−	0.7 × (79)
** ***flgS* (Cj0793)	−	1.7 × (43)	1.3 × (1)	1.7 × (15)
** ***flgR* (Cj1024)	1.2 × (4)	1.1 × (137)	1.3 × (4)	1.2 × (101)
*Flagellar protein secretion (class 1)*				
** *****flgM*** **(Cj1464)**	−	**5 × (1429)**	−	**2.3 × (380)**
** *****fliF*** **(Cj0318)**	−	**6.1 × (1,005)**	−	**7.8 × (1,105)**
** ***flhA* (Cj0882c)	−	1.2 × (82)	−	0.9 × (53)
** ***flhB* (Cj0335)	0.6 × (3)	1.2 × (99)	0.7 × (1)	1.0 × (67)
** ***fliO* (Cj0352)	−	1.4 × (41)	−	0.7 × (1)
** ***fliP* (Cj0820c)	−	1.2 × (29)	−	0.5 × (15)
** ***fliQ* (Cj1675)	−	1.3 × (45)	−	0.9 × (29)
** ***fliR* (Cj1179c)	−	1.2 × (6)	−	0.5 × (5)
** ***fliH* (Cj0320)	−	4.8 × (202)	−	1.3 × (100)
** ***fliI* (Cj0195)	−	3.6 × (442)	−	0.6 × (86)
*Basal body components (classes 1 and 2)*				
** ***fliE* (Cj0526c)	−	2.4 × (268)	−	1.4 × (120)
** ***flgC* (Cj0527c)	−	1.2 × (397)	−	0.9 × (217)
** ***flgB* (Cj0528c)	1.6 × (7)	1.6 × (181)	0.7 × (2)	0.9 × (61)
** *****flgG2*** **(Cj0697)**	−	**43.9 × (8,133)**	−	**77.4 × (9,670)**
** *****flgG*** **(Cj0698)**	1.2 × (1)	1.4 × (253)	**5.3 × (4)**	1.3 × (165)
** ***flgJ* (Cj1463)	−	4.4 × (180)	−	1.0 × (26)
** *****flgI*** **(Cj1462)**	**170.5 × (5,750)**	**52.7 × (12,087)**	**157.1 × (1,666)**	**61.5 × (5,401)**
** *****flgA*** **(Cj0769c)**	0.8 × (2)	**15.8 × (410)**	0.9 × (4)	**3.7 × (104)**
** *****flgH*** **(Cj0687c)**	**200.9 × (1,911)**	**20.1 × (3,288)**	**110.1 × (917)**	**27.6 × (2,487)**
*Flagellar hook components (class 2)*				
** *****flgE*** **(Cj1729c)**	−	**68.2 × (104,324)**	−	**7.1 × (3,967)**
** ***flgD* (Cj0042)	−	3.6 × (1015)	−	2.0 × (284)
** ***flgE2* (Cj0043)	−	2.5 × (1045)	−	1.1 × (250)
** ***fliK* (Cj0041)	−	4.1 × (613)	−	1.2 × (89)
Cj0040*	**356.2 × (3,389)**	**110.6 × (9,277)**	**38 × (230)**	**20.4 × (727)**
** ***flgK* (Cj1466)	−	0.7 × (4)	−	1.0 × (141)
** ***flgL* (Cj0887c)	−	2.0 × (484)	2.3 × (74)	0.9 × (193)
*Flagellar filament components (classes 2 and 3)*				
** *****flaA*** **(Cj1339c)**	**304.5 × (693,471)**	**111 × (473,588)**	**324.7 × (158,590)**	**45.3 × (138,159)**
** *****flaB*** **(Cj1338c)**	**58.8 × (915)**	**14.1 × (17,880)**	**59.4 × (1,170)**	**14.9 × (29,530)**
** *****fliD*** **(Cj0548)**	−	**6.8 × (4,348)**	−	**5.4 × (3,929)**
** ***fliS* (Cj0549)		1.6 × (149)		1.3 × (165)
** ***flaC* (Cj0720)	1.2x (344)	1.2 × (1,298)	1.3 × (239)	1.2 × (1,237)
*Other enriched genes (>5x) involved in flagella formation*				
** *****pseB*** **(Cj1293)**	**119.7 × (2,298)**	**9.5 × (2,280)**	**34.5 × (470)**	4.1 × (759)
** *****pseI*** **(Cj1317)**	1.7 × (22)	**7.2 × (864)**	2.3 × (14)	0.8 × (111)
** *****flaG*** **(Cj0547)**	**346.1 × (11,077)**	**72.4 × (18,150)**	**168.5 × (3,701)**	**84.2 × (16,012)**
** *****motA*** **(Cj0337c)**	**10.3 × (89)**	1.8 × (660)	1.4 × (16)	0.8 × (271)
** Cj0951c**	−	**15.2 × (79)**	2 × (3)	1.3 × (194)
** Cj0248**	**5.5 × (120)**	1.8 × (387)	1.1 × (38)	0.9 × (257)
** *****flhX*** **(Cj0848c)**	−	**7.5 × (13)**	−	1.5 × (7)

CoIP, co-immunoprecipitation; UTR, untranslated region; ORF, open reading frame.

Classification of flagellar genes is based on ref. 75. Transcripts with >5-fold enrichment in cDNA read counts in the CsrA-3xFLAG versus control coIP libraries are highlighted in bold. Numbers in brackets indicate the absolute cDNA read counts in the CsrA-3xFLAG coIP libraries.

^*^Cj0040 (unknown function) is the first gene of the hook gene operon.
