# Supplementary material for: The CsrA-FliW network controls polar localization of the dual-function flagellin mRNA in Campylobacter jejuni
Source: Nat Commun. 2016 May 27;7:11667. doi: 10.1038/ncomms11667 (PMC4894983; doi:10.1038/ncomms11667)
Supplement: Supplementary Information — Supplementary Figures 1-19, Supplementary Tables 1-8, Supplementary Methods, Supplementary References [file ncomms11667-s1.pdf]

Supplementary Figures

Supplementary Figure 1

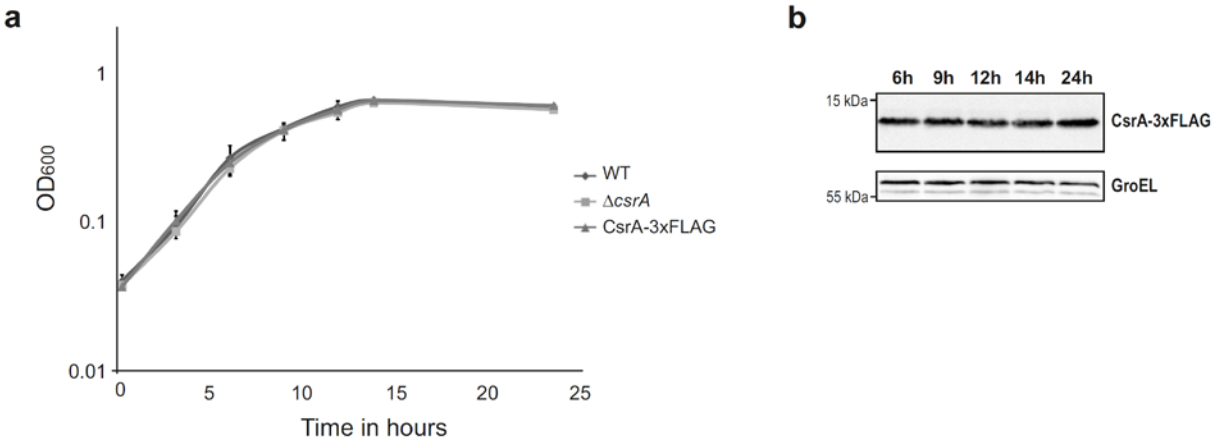

Supplementary Figure 1. Growth curves and expression of *C. jejuni* CsrA in strain NCTC11168. (a) Semi-log growth curves over 24 h for *C. jejuni* NCTC11168 wild-type (WT), Δ*csrA* and CsrA-3xFLAG tagged strains grown in Brucella broth in duplicate. Error bars are mean ± s.e.m. **(b)** Western blot analysis of CsrA-3xFLAG expression during growth in liquid culture in *C. jejuni* strain NCTC11168. Total protein samples corresponding to 0.05 OD_{600} were loaded for different time points. GroEL was probed as sample processing control on a separate blot.

Supplementary Figure 2

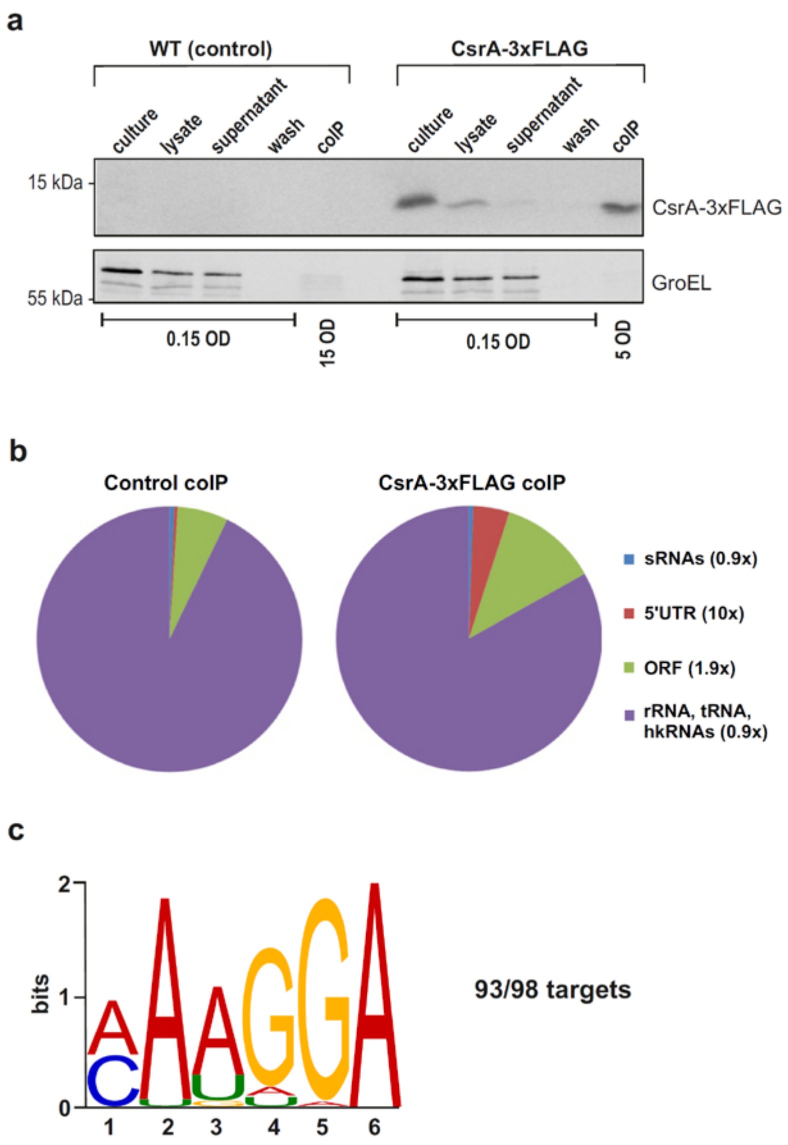

Supplementary Figure 2. RIP-seq of CsrA-3xFLAG in strain 81-176. (a) Western blot of protein samples from CsrA-3xFLAG and control coIPs from strain 81-176. The protein amount loaded in each lane corresponds to the OD_{600} of cells as indicated below. GroEL was probed as sample processing control on a separate blot. **(b)** Pie charts showing the relative proportions of mapped cDNAs of different RNA classes in the control and CsrA-3xFLAG coIP libraries from strain 81-176. Values in brackets for each RNA class denote its relative enrichment in the CsrA-3xFLAG vs. control coIP. **(c)** Consensus motif for CsrA determined by MEME using peak sequences enriched more than 5-fold in the CsrA-3xFLAG coIP from *C. jejuni* strain 81-176.

Supplementary Figure 3

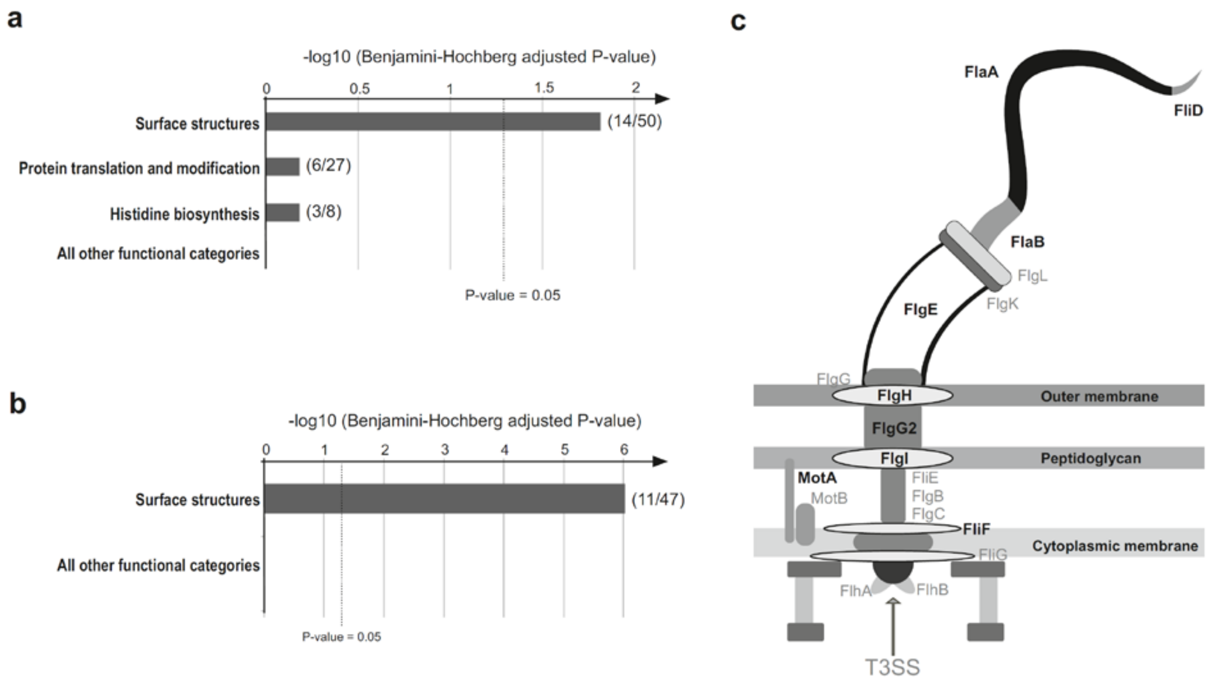

Supplementary Figure 3. Statistical analysis for overrepresentation of functional categories among potential CsrA target genes. (a), (b) Statistical analysis was performed on all genes with more than 5-fold enrichment in their 5'UTR and/or coding sequence in the CsrA-coIP in comparison to non-enriched genes. P-values, adjusted using the Benjamini-Hochberg method, were calculated for overrepresentation of enriched genes in each functional category in strain NCTC11168 **(a)** and strain 81-176 **(b)**. Values in brackets denote the number of enriched genes in the particular functional category (only functional categories with non-zero $\log_{10}$ P-values are shown for clarity). Functional categories are based on reannotation of the NCTC11168 genome[1]. **(c)** Schematic representation of structural components of the *Campylobacter* flagellum. Proteins encoded by mRNAs which showed >5-fold enrichment in the CsrA coIP are marked in black and bold. T3SS: Type III secretion system.

Supplementary Figure 4

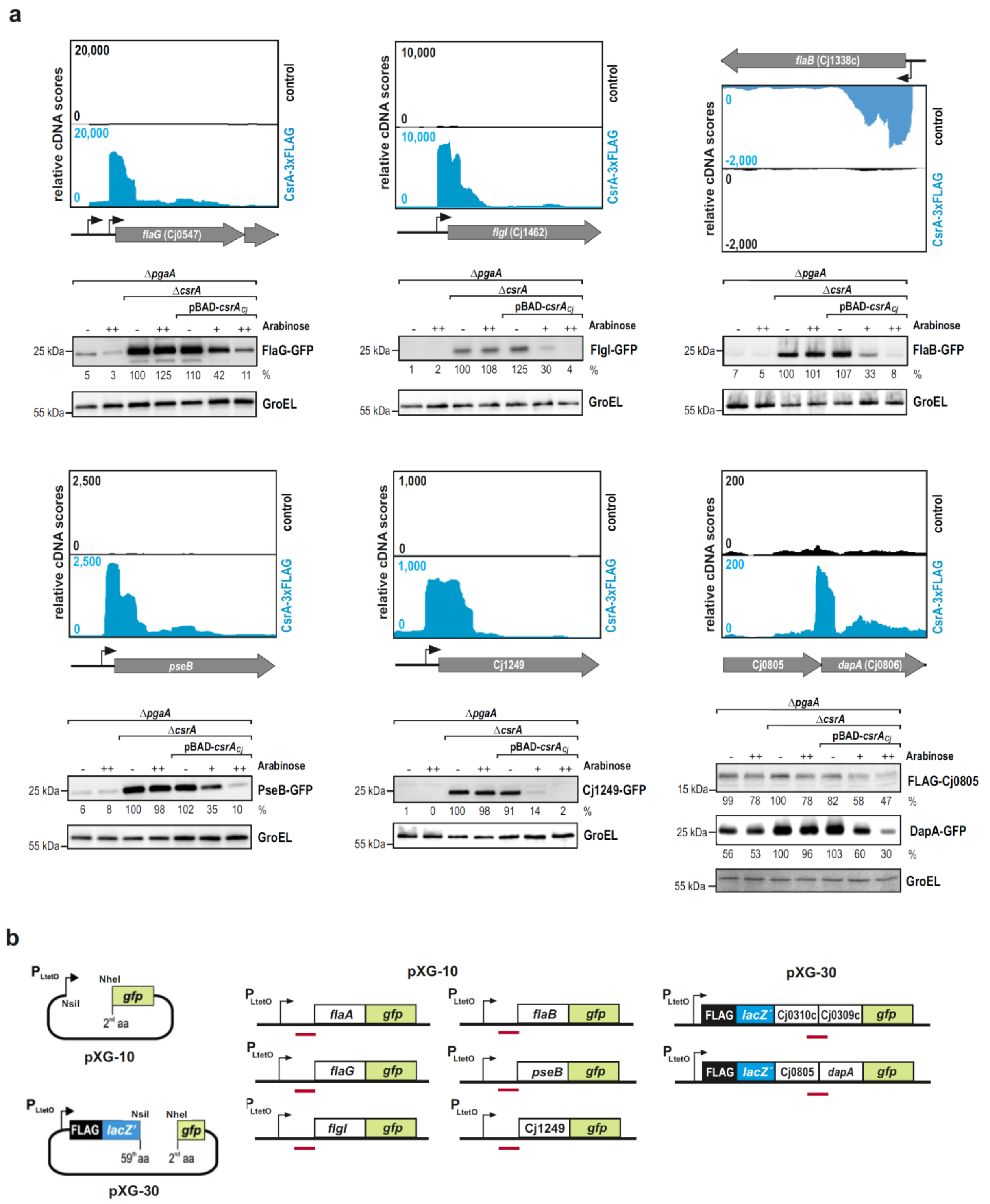

Supplementary Figure 4. Validation of potential CsrA targets using a GFP reporter system in *E. coli*. (a) *(Top panels)* Examples of enrichment patterns indicating potential CsrA binding sites in 5'UTRs (*flgG, flgI, flaB, pseB,* and Cj1249 mRNAs) and between genes in polycistronic transcripts (Cj0805-*dapA* operon; encoding a zinc protease and dihydrodipicolinate synthase) that were tested in the *E. coli* system. Mapped cDNA reads are shown for the control (black) and

CsrA-3xFLAG coIP (blue) libraries in strain NCTC11168. ORFs are indicated by grey arrows and TSS - based on dRNA-seq[2] - by black arrows, respectively. *(Lower panels)* Western blot analysis using anti-FLAG and anti-GFP antibodies of reporter translational fusions to potential *C. jejuni* CsrA target genes in *E. coli* Δ*pgaA*, Δ*pgaA/*Δ*csrA,* and Δ*pgaA/*Δ*csrA* complemented with plasmid pGD72-3 carrying arabinose-inducible *csrA*-Strep from *C. jejuni.* Strains were grown to late log phase in LB medium or LB media supplemented with 0.001% (+) or 0.003% (++) L-arabinose. **(b)** Overview of GFP reporter plasmids pXG-10 and pXG-30[3] used to validate 5'UTR and intergenic/ORF targets, respectively. For putative targets in 5'UTRs, the entire leader, as well as the first few codons, were cloned as a translational fusion to *gfp* in low-copy vector pXG-10 (pSC101* origin). Fusions to *gfp* were made downstream of the +1 (TSS) site of the P_L promoter and were transcribed from a constitutive λ PLtetO-1 promoter (PL derivative). To examine CsrA binding between genes in polycistrons, or in downstream regions of ORFs, the operon plasmid pXG-30 was used. The C-terminus of the upstream ORF was fused in-frame after a short artificial reading frame composed of a FLAG epitope and truncated *lacZ* gene, whereas the N-terminus of the downstream gene was fused in frame to *gfp*, thus mimicking operon mRNA expression. Putative CsrA binding regions are marked by a red line.

Supplementary Figure 5

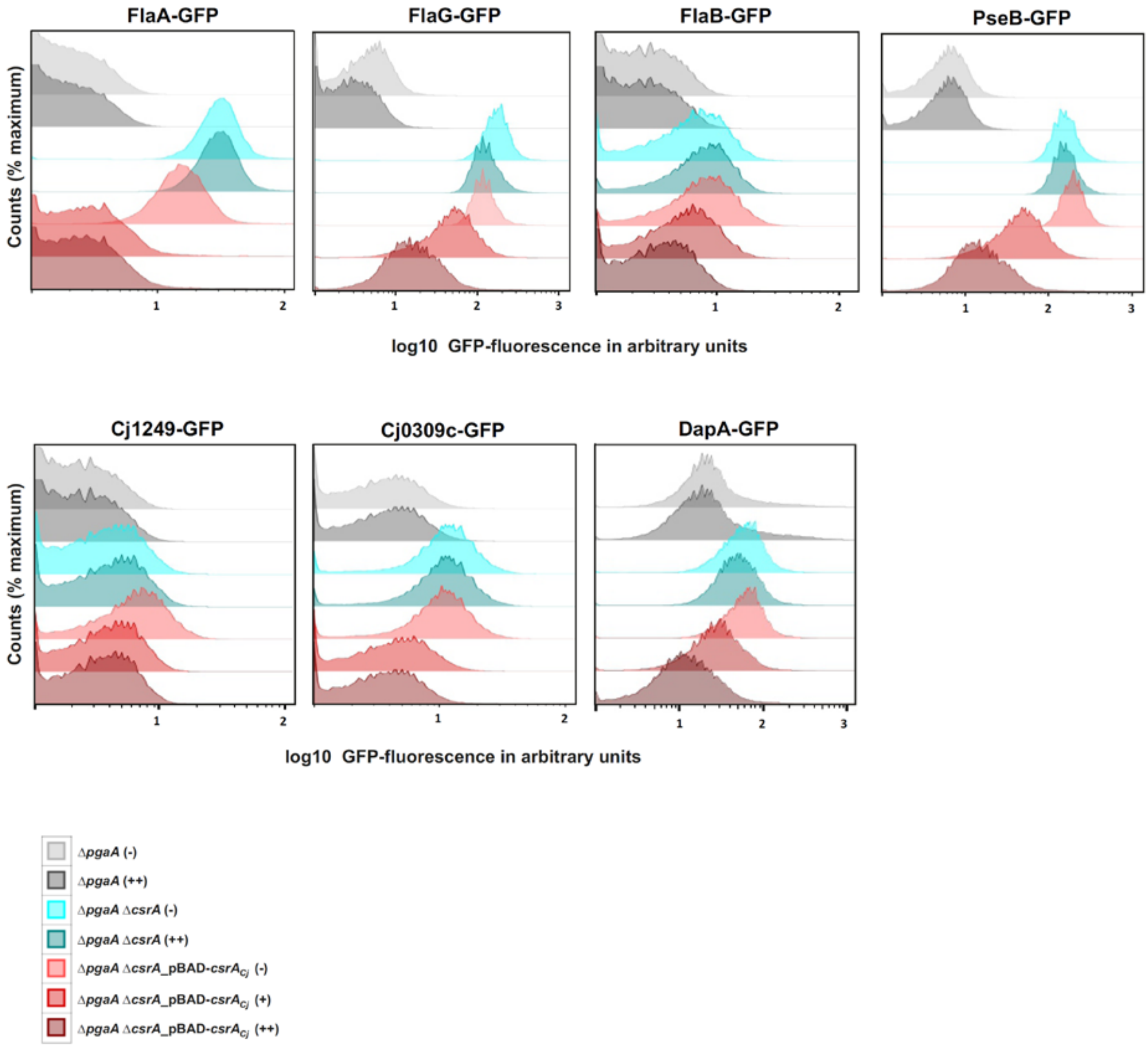

Supplementary Figure 5. FACS analysis of GFP reporter fusions. pXG-10- or pXG-30-based GFP reporter plasmids from Supplementary Fig. 3b were introduced into *E. coli* strains Δ*pgaA*, Δ*pgaA*/Δ*csrA*, or Δ*pgaA*/Δ*csrA* complemented with arabinose-inducible *C. jejuni csrA*-Strep. All strains were grown to late log phase in LB or LB supplemented with 0.001% (+) or 0.003% (++) L-arabinose, and GFP levels were measured by flow cytometry. Data acquired in each experiment is plotted in fluorescence histograms generated from all events measured (50,000 events). Cellular fluorescence is given in arbitrary units (GFP intensity). Regulation by CsrA is visible as a shift of the peak of fluorescence curves to the right (higher GFP intensity) in the Δ*pgaA*/Δ*csrA* background and a shift to the left upon complementation with CsrA-Strep. Please note that levels of FlgI-GFP were not detectable in FACS due to low expression or fluorescence of this fusion.

Supplementary Figure 6

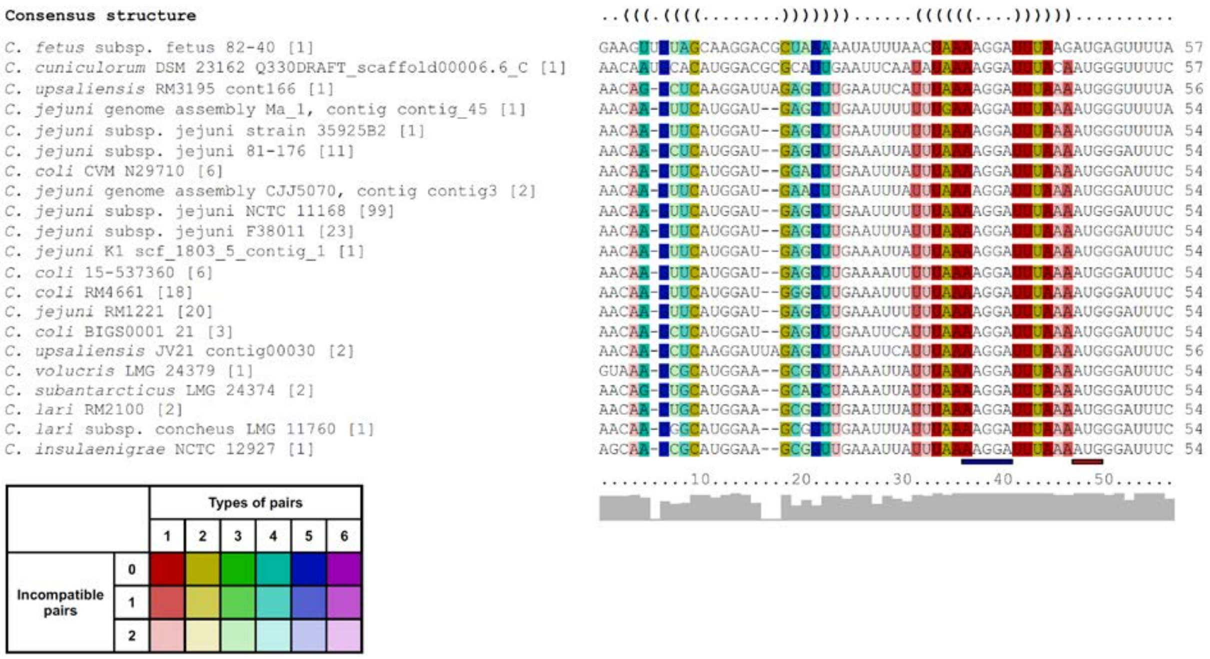

Supplementary Figure 6. Structure annotated sequence alignment of *flaA* 5'UTR. Structure-annotated sequence alignment based on representative unique sequences of the *flaA* 5'UTR and the first 10 nt of the coding region from different *Campylobacter* species. The numbers in square brackets indicate how often an identical sequence occurred in the original complete alignment (see Supplementary Fig. 7). The consensus structure is shown in bracket-dot notation at the top and sequence conservation as a bar chart at the bottom. The color legend was adapted from the RNAalifold online help (http://rna.tbi.univie.ac.at/help.html) and reflects how many different kinds of nucleotide combinations support a certain base pair. Here, paler colors indicate that a base pair cannot be formed by all sequences in the alignment. The RBS and start codon of *flaA* mRNA are indicated by blue and red bars below the alignment, respectively.

Supplementary Figure 7

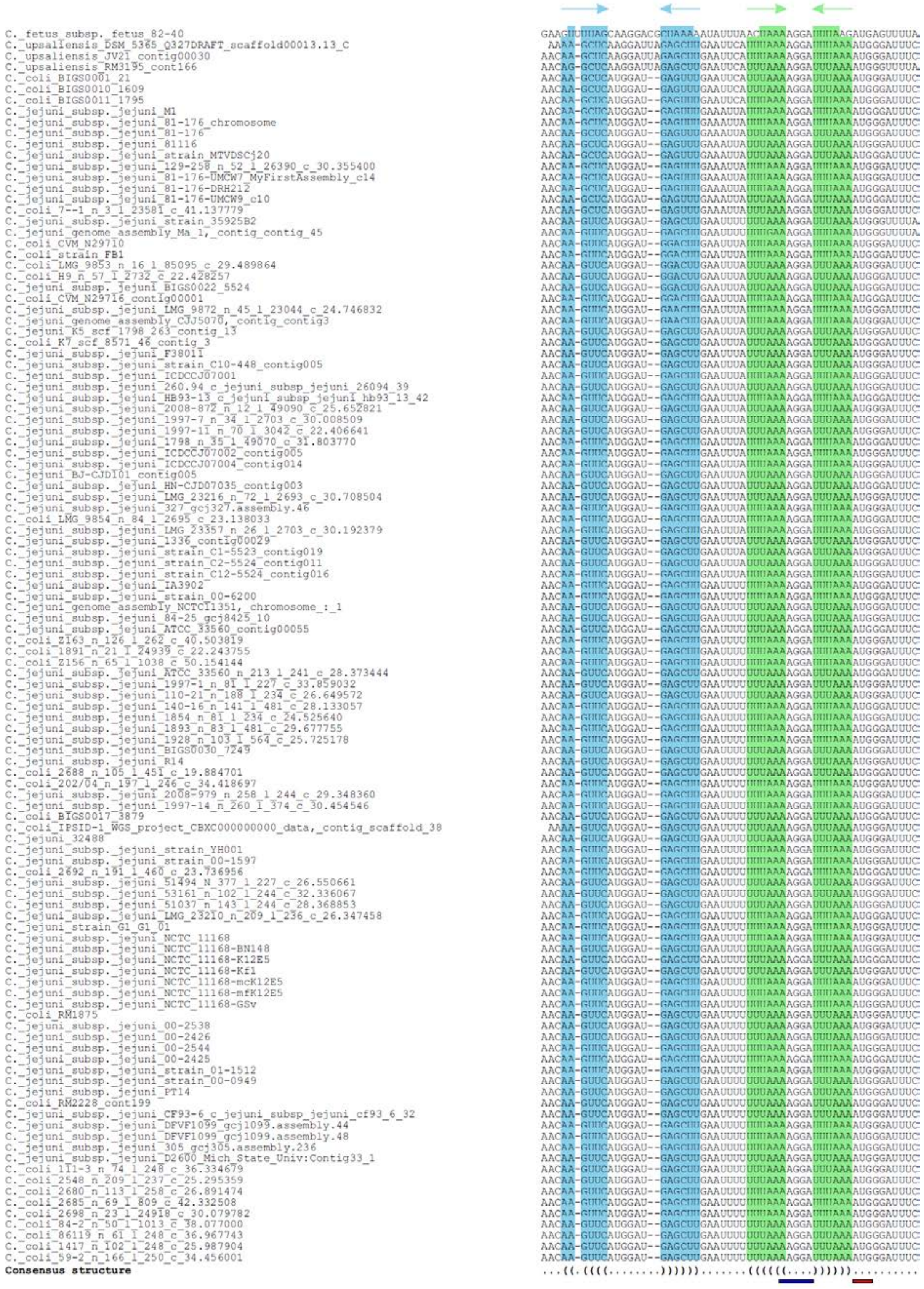

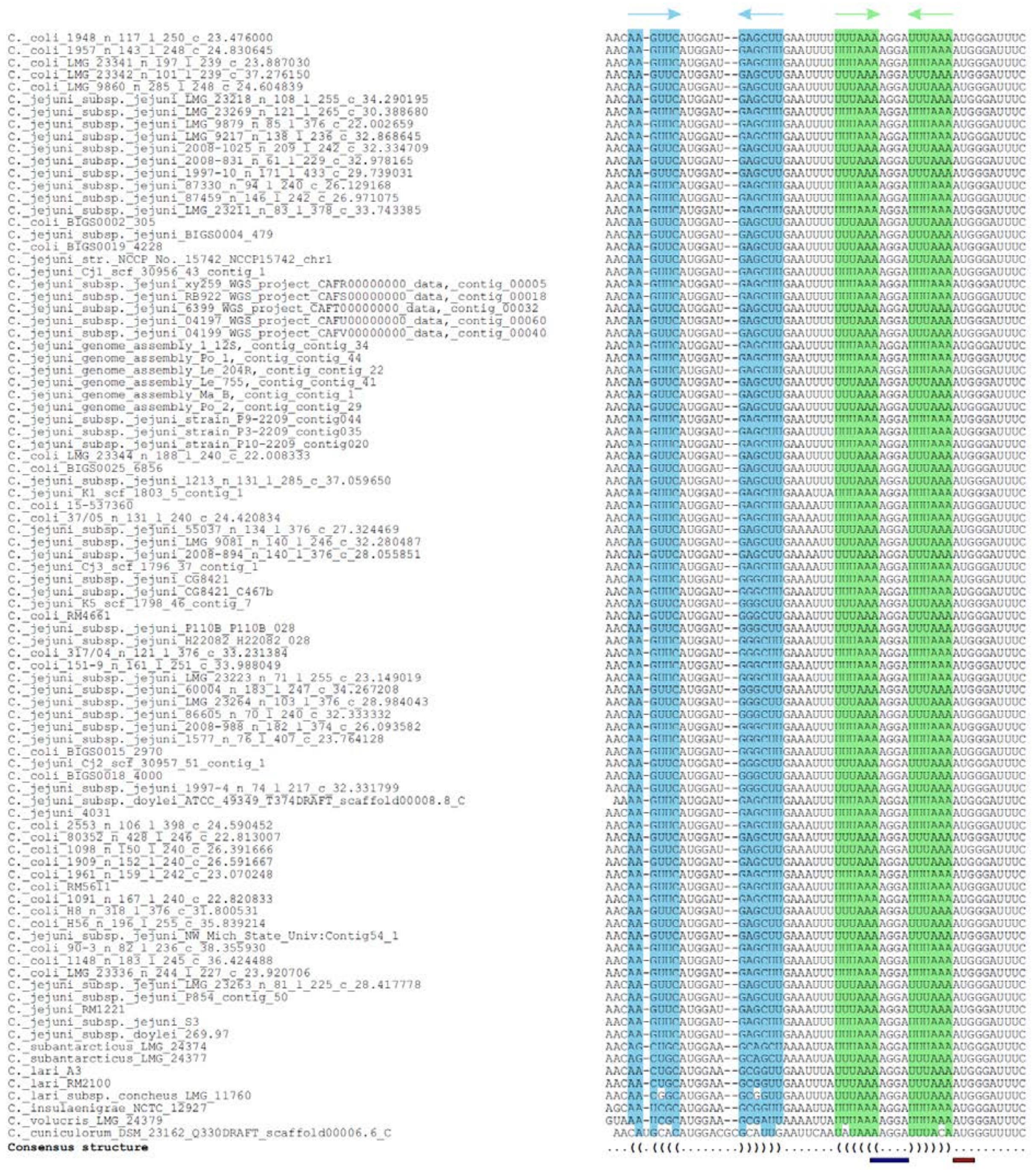

Supplementary Figure 7. Sequence alignment of *flaA* leaders from *Campylobacter* species. Sequence alignment of redundant sequences for the *flaA* 5'UTR and the first 10 nt of the coding region from different *Campylobacter* species. The consensus structure calculated based on the collapsed version of the alignment (see Supplementary Fig. 6) is shown at the bottom of each page. The blue and green arrows at the top mark stem loops in the consensus structure while pointing to the unpaired loop region, respectively. The nucleotides of each sequence below the stems are marked in the respective color if the base pair can be formed and have a white background otherwise. The RBS and start codon of *flaA* mRNA are indicated by blue and red bars below the alignment, respectively.

Supplementary Figure 8

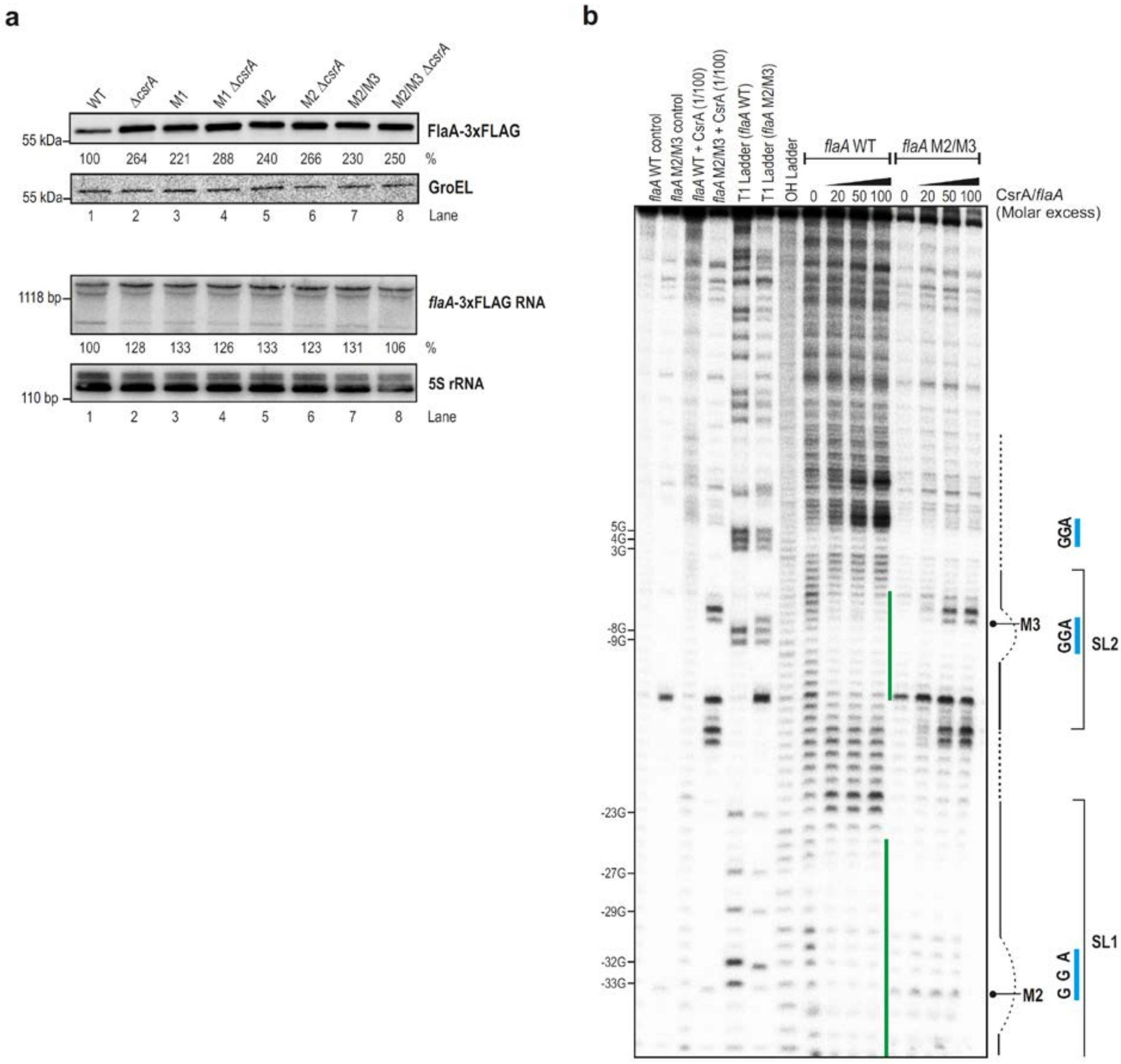

Supplementary Figure 8. Analysis of regulation and binding of CsrA to *flaA* mutant leaders. (a) Representative Western and Northern blot of FlaA-3xFLAG and its mRNA in various *flaA* 5'UTR mutant strains (from main Fig. 2b) from liquid cultures in log phase. Anti-FLAG antibody was used to detect FlaA-3xFLAG. See main Fig. 2b for M1, M2 and M3 mutations. GroEL was probed as sample processing control on a separate blot. **(b)** Footprinting assays of ~0.2 pmol P^{32} labeled *flaA* (WT or M2/M3 mutant) leaders in the absence or presence of increasing *C. jejuni* CsrA concentrations (CsrA/*flaA* molar ratio of 0, 20, 50 and 100) using lead(II) acetate. Untreated *flaA* leader alone, or *flaA* leader incubated with 100-fold excess CsrA, served as controls. Partially RNase T1- or alkali (OH)-digested *flaA* WT and M2/M3 leaders are included as ladders. Blue lines: three GGA motifs in the WT *flaA* leader; green lines: regions protected from cleavage upon increasing CsrA concentration. Bands representing the M2 or M3 mutations are marked next to the gel.

Supplementary Figure 9

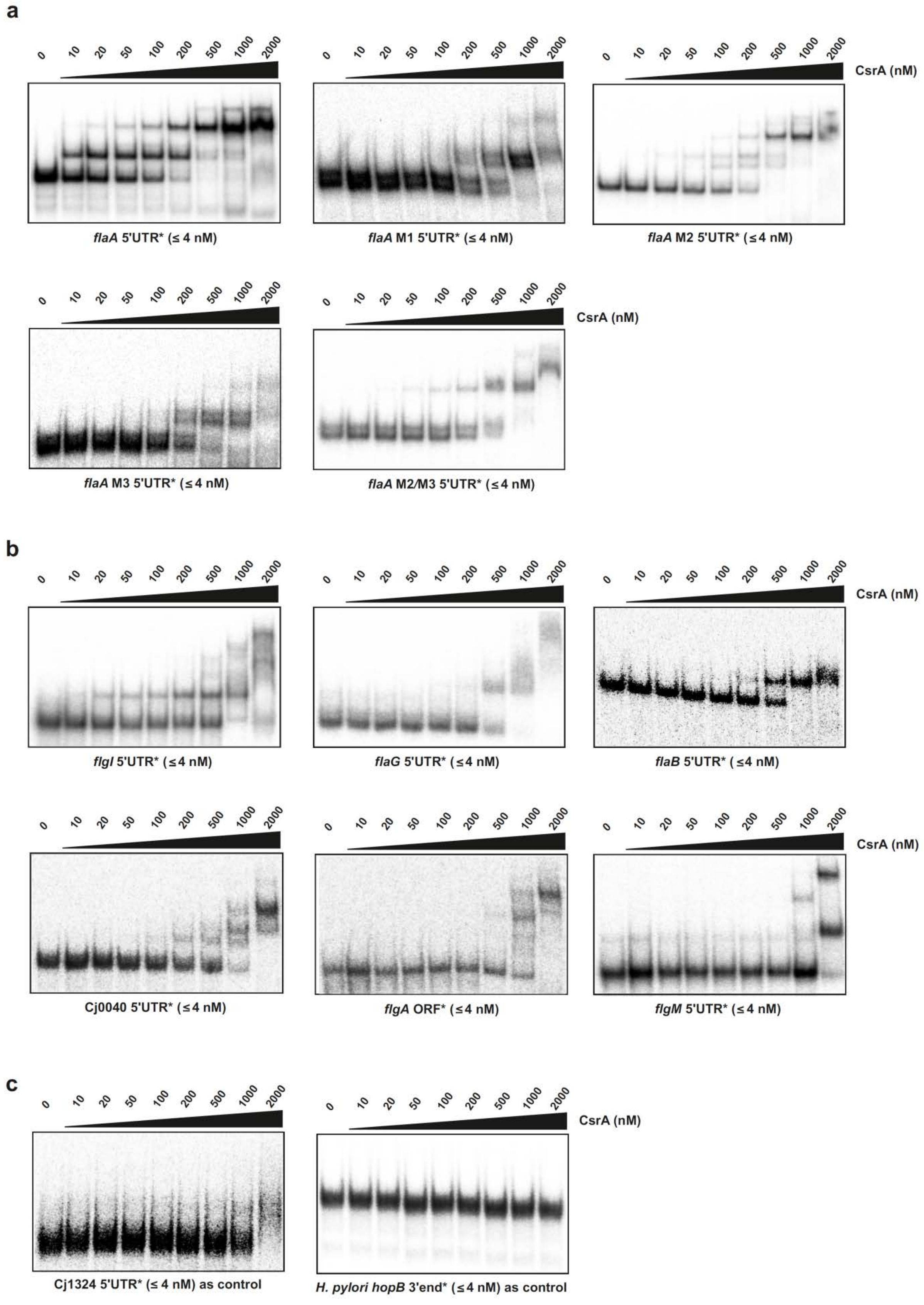

Supplementary Figure 9. *In vitro* gel-shift assays of 5'-labeled T7-transcripts and purified *C. jejuni* CsrA. (a) Gel-shift assays with 5'-labeled WT *flaA* leader and its mutant variants (M1, M2, M3, and M2/M3) with increasing concentrations of CsrA. M1: GGA→AAA in stem-loop 1 (SL1, see Fig. 2a); M2: GGA>UGA in SL1; M3: GGA>GGG in stem-loop 2 (SL2); M2/M3: combination of M2 and M3. **(b)** Gel-shift assays of T7-transcribed, 5'-labeled RNAs of flagellar targets with increasing concentrations of CsrA. **(c)** Gel-shift assays with negative controls using the leader of Cj1324 from *C. jejuni* (one GGA, not enriched in either coIP) and a fragment of the unrelated *hopB* mRNA (no GGAs, from *Helicobacter pylori* G27).

Supplementary Figure 10

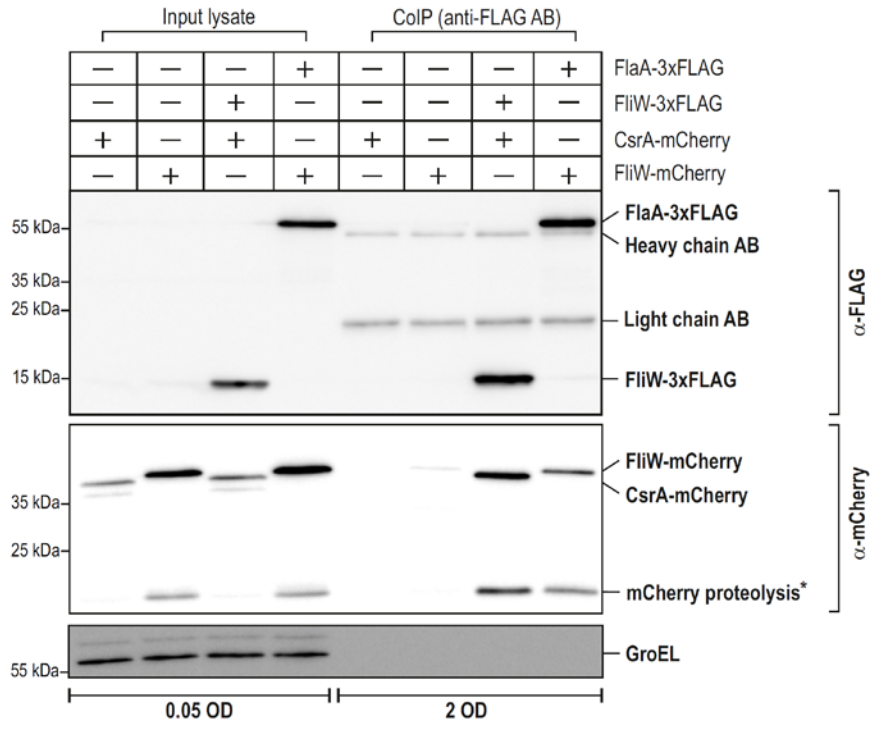

Supplementary Figure 10. Protein-protein coIP confirms direct interactions of FliW with CsrA and FlaA. CsrA-mCherry and FliW-mCherry were specifically co-purified in a coIP of FliW-3xFLAG and FlaA-3xFLAG, respectively, using an anti-FLAG antibody. In a negative control reaction with non-tagged FliW and FlaA, CsrA-mCherry and FliW-mCherry were not pulled down. Western Blots were performed for the input lysates and coIP protein samples (FLAG) using anti-FLAG and anti-mCherry antibodies. GroEL served as loading control for the input lysate samples on the mCherry blot and was not detected in the coIP fraction. (*Please note that the lower band represents partially hydrolysed mCherry resulting from sample preparation[4].)

Supplementary Figure 11

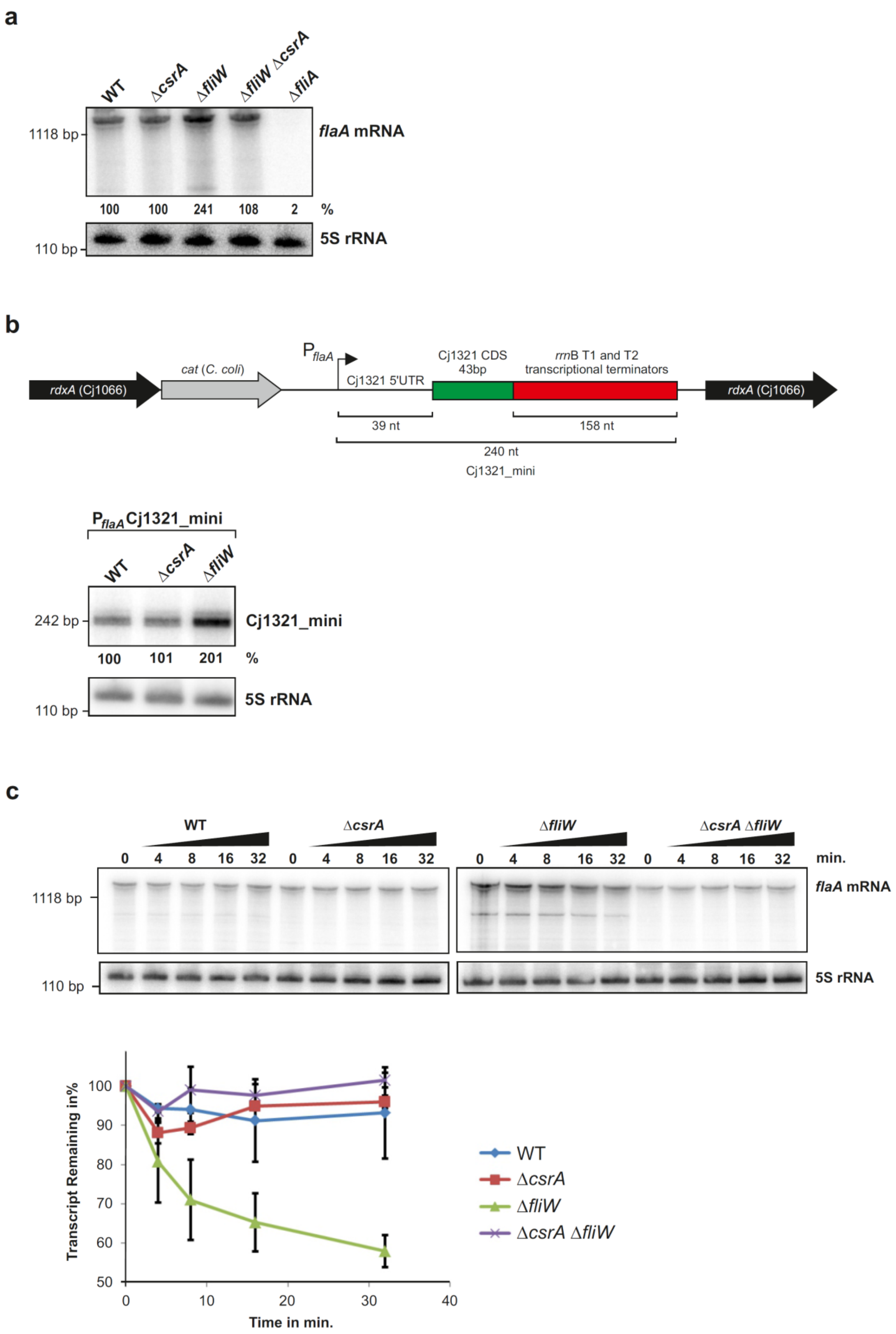

Supplementary Figure 11. Examination of effects of FliW on *flaA* transcription and translation. (a) Northern blot analysis of *flaA* mRNA using oligonucleotide probe CSO-0486, as

well as 5S rRNA (loading control, CSO-0192) in *C. jejuni* NCTC11168 wild-type and the indicated mutant strains grown to mid-log phase. Relative expression levels are quantified below the blot. **(b)** (*top*) Schematic representation of a Cj1321_mini gene construct that was expressed from the P_{flaA} promoter and was integrated into the unrelated *rdxA* locus of *C. jejuni*. The Cj1321 gene is not a CsrA target according to the coIP results and thus should be independent of CsrA. The Cj1321 5'UTR and the first 42 bp of the coding sequence were fused to a stable *rrnB* terminator to express as stable mini transcript under control of the P_{flaA} promoter. Northern blotting was used to monitor expression of the stable Cj1321_mini transcript, which was used as a transcriptional reporter for the *flaA* promoter. The ~240-nt long Cj1321_mini transcript was detected by Northern blot analysis of total RNA from *C. jejuni* cells expressing Cj1321_mini either in the wildtype, Δ*csrA*, or Δ*fliW* background using oligonucleotide probe CSO-2746. Probing for 5S rRNA (CSO-0192) served as a loading control. **(c)** *flaA* mRNA rifampicin stability assay. Northern blot probed for *flaA* mRNA in *C. jejuni* wildtype (WT) and the mutant strains over a time course after rifampicin addition (0-32 min) using oligonucleotide probe CSO-2835. Averaged quantification of *flaA* mRNA transcript levels over time from two independent rifampicin stability assays is shown. Error bars indicate mean ± s.e.m.

Supplementary Figure 12

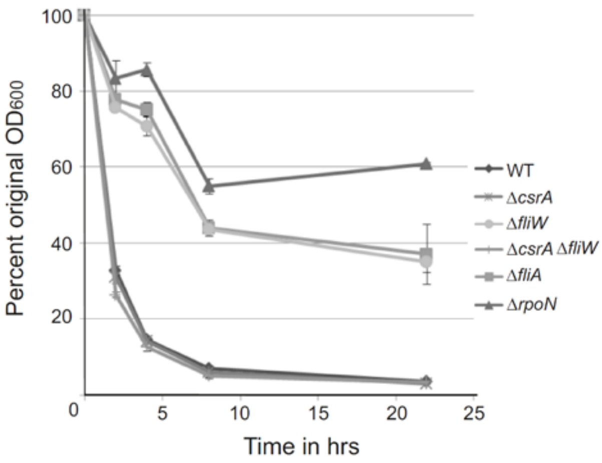

Supplementary Figure 12. Influence of *csrA* and *fliW* deletion on autoagglutination. Autoagglutination assay of *C. jejuni* WT and mutant strains (OD_{600} of supernatants of 1.0 OD bacterial suspensions grown in Brucella broth at the indicated time points) in PBS. Error bars indicate the s.e.m.

Supplementary Figure 13

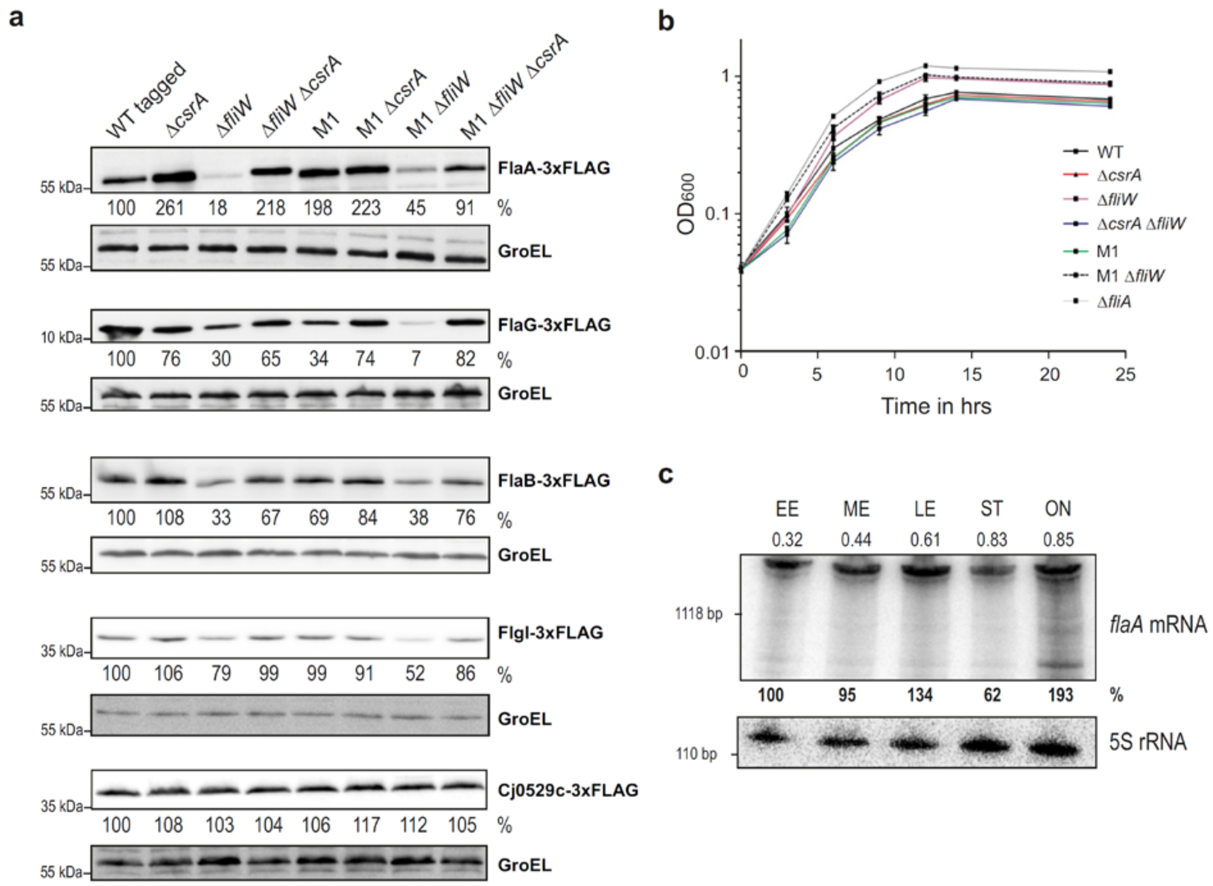

Supplementary Figure 13. Representative Western blots of 3xFLAG-tagged CsrA targets in various mutant strains and Northern blot of *flaA* mRNA over growth in the WT strain. **(a)** FlaA-3xFLAG, FlaG-3xFLAG, FlaB-3xFLAG, and FlgI-3xFLAG levels, as well as Cj0529c-3xFLAG levels as a negative control, were examined by Western blot in *C. jejuni* NCTC11168 wildtype (WT), Δ*csrA*, Δ*fliW*, Δ*csrA*/Δ*fliW,* M1*,* M1/Δ*csrA*, M1/Δ*fliW,* and M1/Δ*fliW*/Δ*csrA* strains. Cells were grown to mid-log phase in liquid culture, and protein samples (amounts corresponding to an OD_{600} of cells of 0.02 for FlaA-3xFLAG, 0.075 for FlaG-3xFLAG, or 0.05 for FlaB-3xFLAG, FlgI-3xFLAG and Cj0529c-3xFLAG) were analyzed by Western blotting with an anti-FLAG antibody. GroEL levels served as loading control for FlgI-3xFLAG and Cj0529c-3XFLAG and was probed as sample processing control on separate blots for FlaA-, FlaG- and FlaB-3xFLAG. **(b)** Semi-log growth curves in Brucella broth over 24 h for the untagged strains from **(a)** based on 2 biological replicates. Error bars indicate mean ± s.e.m. **(c)** Northern blot analysis of *flaA* mRNA using RNA extracted from *C. jejuni* samples collected at different growth phases (EE-Early Exponential, ME-Mid Exponential, LE-Late Exponential, ST-Stationary and ON-Overnight). The OD_{600} of the culture is also indicated below each phase. 5S rRNA was probed as a loading control.

Supplementary Figure 14

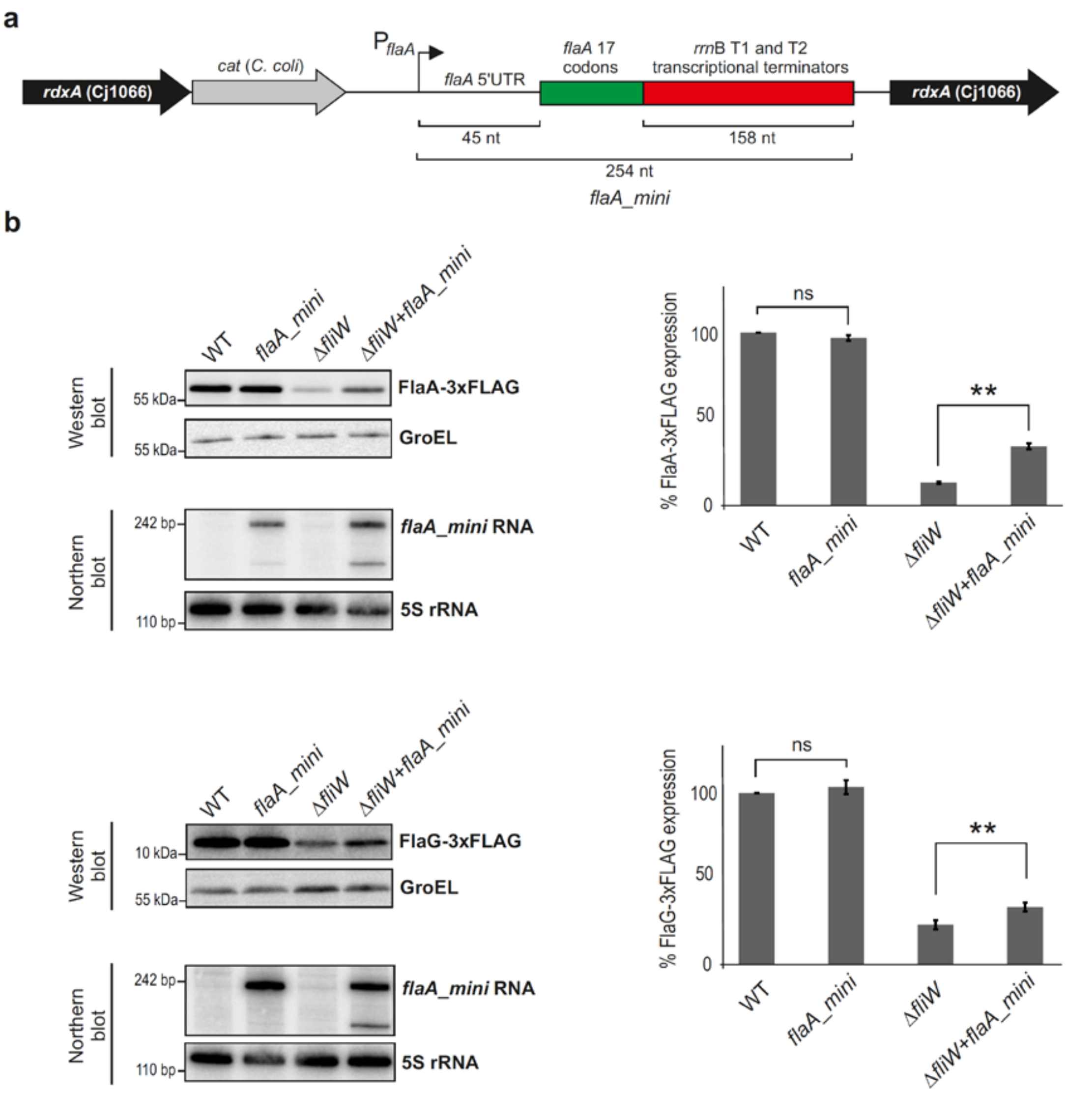

Supplementary Figure 14. An ectopically-expressed *flaA* mini-gene can partially complement CsrA-mediated effects on FlaA and FlaG translation upon *fliW* deletion. **(a)** Schematic representation of a *flaA_mini* gene construct expressed from the *rdxA* locus of *C. jejuni*. **(b)** *(Left)* Representative Western blots of FlaA-3xFLAG and FlaG-3xFLAG used for the quantifications on the right and Northern blots of *flaA_mini* RNA expression from liquid cultures in log phase. Anti-FLAG antibody was used to detect the tagged proteins. GroEL was probed as sample processing control on separate blots. *(Right)* Quantification of FlaA-3xFLAG (top panel) and FlaG-3xFLAG (bottom panel) determined by Western blot in the indicated strains (n ≥ 4). Error bars indicate mean ± s.e.m (**P<0.01). Deletion of *fliW* leads to strong CsrA-mediated translational repression of *flaA*-3xFLAG and *flaG*-3xFLAG due to release of CsrA repression in the absence of the FliW protein antagonist. Expression of the stable *flaA_mini* transcript partially relieves CsrA-mediated translational repression of *flaA*-3xFLAG and *flaG*-3xFLAG upon *fliW* deletion, indicating that it can sequester CsrA and act as an antagonist of CsrA activity.

Supplementary Figure 15

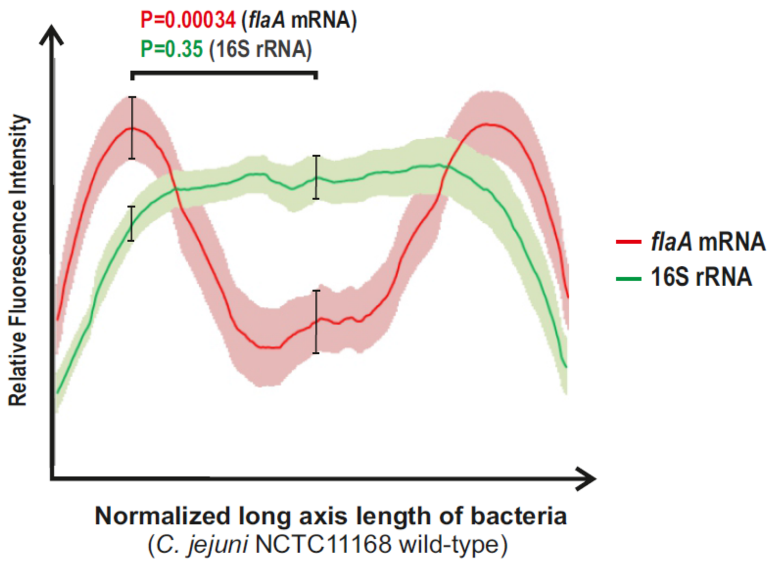

Supplementary Figure 15. Averaged fluorescence intensity curves for *flaA* mRNA and 16S rRNA FISH signals. Averaged fluorescence intensities (based on 10 cells) from RNA-FISH analysis of 16S rRNA (green) and *flaA* mRNA (red) plotted along the long cell axis for *C. jejuni* NCTC11168 WT. The shaded regions along the curves mark the boundary of errors bars (±s.e.m.) of 320 points along the long axis. The points taken for statistical analysis are highlighted by black error bars (Student's *t*-test). These points correspond to the pole and the mid-cell of the bacterium.

Supplementary Figure 16

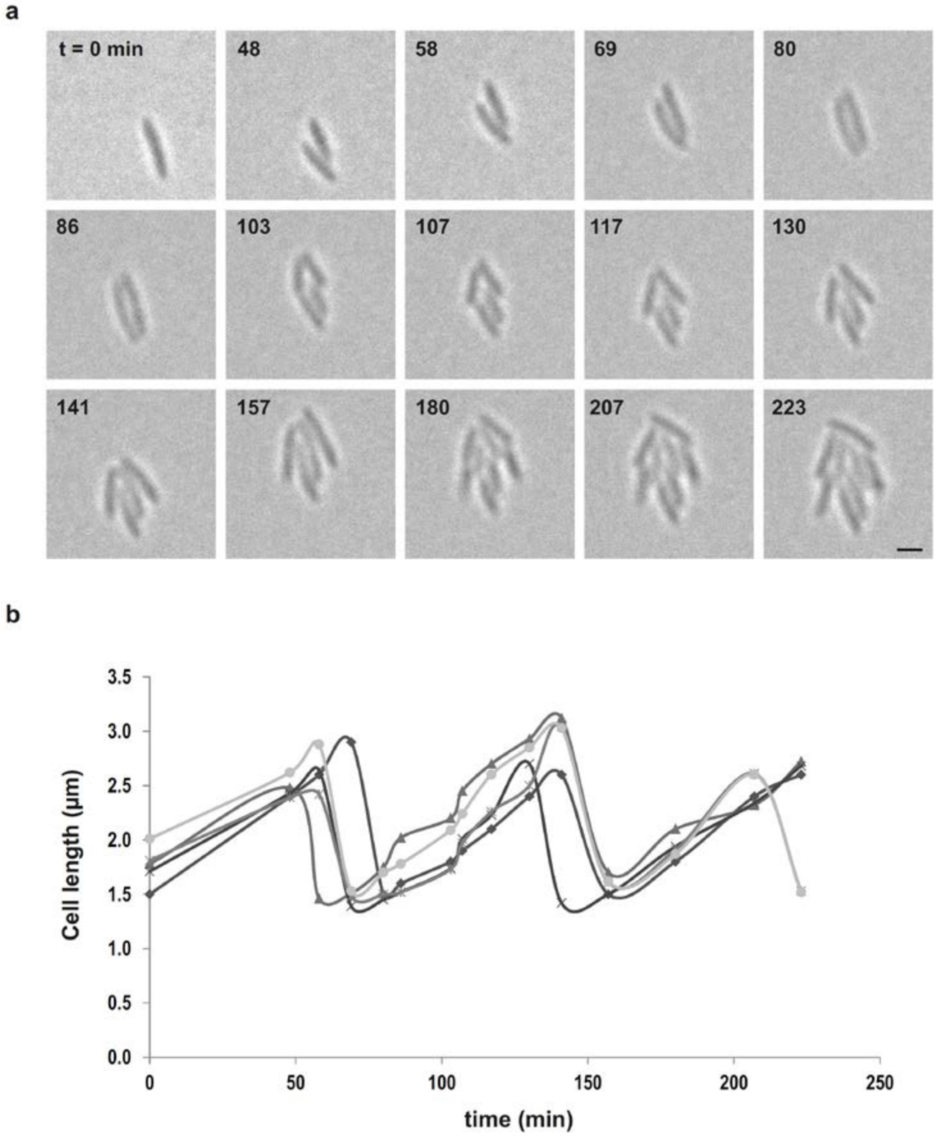

Supplementary Figure 16. Live-cell imaging of growth of non-motile *C. jejuni* over 2-3 generations. **(a)** A dividing non-motile (Δ*fliA*) *C. jejuni* cell (collected from Brucella broth culture in log phase) was imaged under a fluorescence microscope in bright field mode. Fifteen images were taken over a period of 223 min and 3 cell divisions. The black bar (lower right) represents 1 µm in length. **(b)** Cell lengths were measured over 2-3 divisions for five representative cells using ImageJ and were plotted along the time frame (each curve represents one cell). Please note that the cell lengths were longer (up to ~3 µm) compared to those of the fixed WT cells used in the FISH analysis (Figure 6, up to ~2 µm). This length difference could be due to slightly different morphology of the non-motile strain or different growth conditions (aerobic) used during microscopy.

Supplementary Figure 17

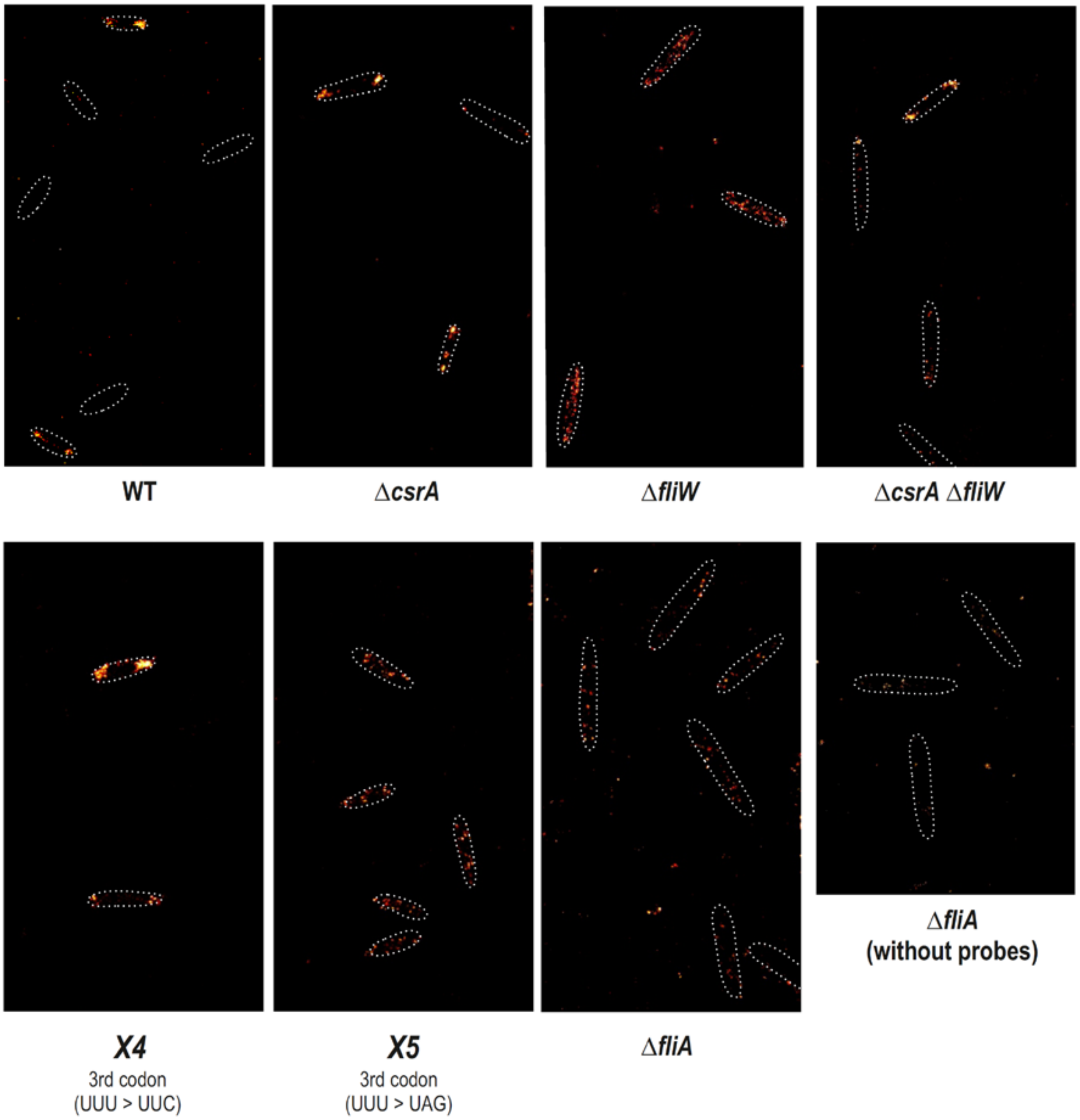

Supplementary Figure 17. Super-resolution imaging of *flaA* mRNA. RNA-FISH analysis of *flaA* mRNA (14 Cy5-labeled oligos) in the indicated *C. jejuni* strains using *d*STORM imaging. Cell boundaries from bright field images are depicted by white dotted lines. As a negative control, the *C. jejuni fliA* deletion strain was analyzed with and without probes to check for background signals.

Supplementary Figure 18

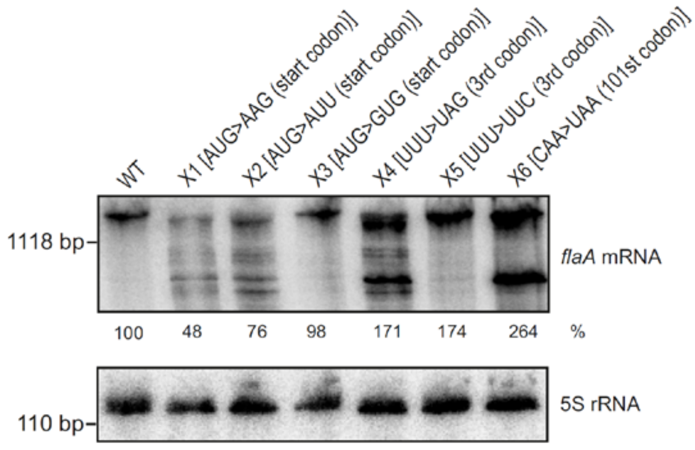

Supplementary Figure 18. Northern blot analysis of *flaA* mutant mRNAs. Northern blot analysis of *flaA* mRNA using oligonucleotide probe CSO-2835, as well as of 5S rRNA (loading control, probe CSO-0192) in *C. jejuni* NCTC11168 wild-type and the indicated mutant strains with point mutations in the *flaA* coding region. RNA was extracted from cells grown to mid-log phase.

Supplementary Figure 19

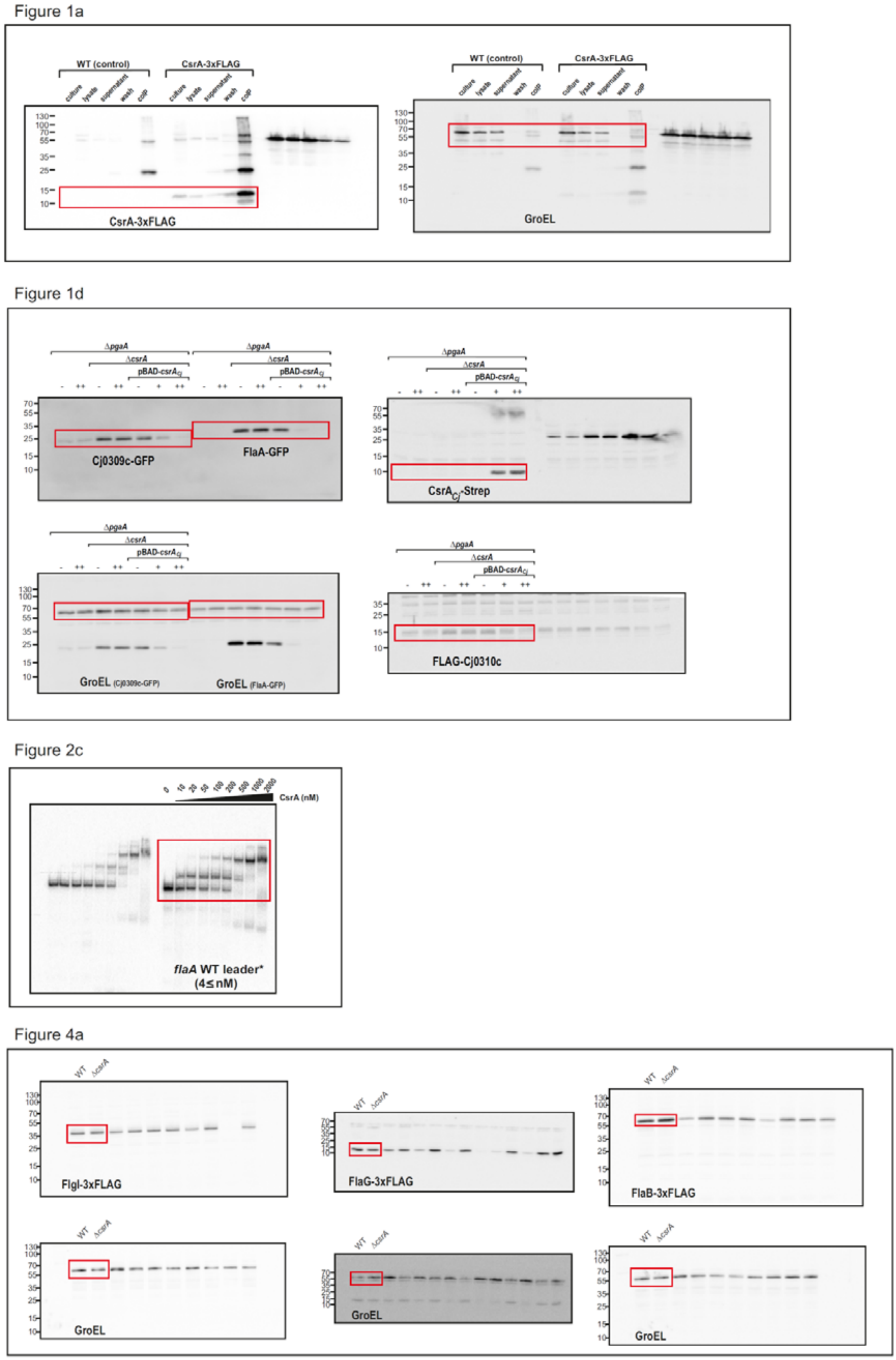

Supplementary Figure 19. Uncropped images of all Western blots and gels shown in the main Figures. The cropped parts that are shown in the main Figures are marked by red boxes.

Supplementary Tables

Supplementary Table 1. Mapping statistics of *C. jejuni* CsrA coIP RNA-seq libraries. The table indicates the total number of sequenced cDNA reads considered in the analysis, the number of reads that were removed due to insufficient length (<12 nt) after poly(A)-tail clipping (before read mapping), the number of reads that were successfully mapped to the reference genomes (or the pVir and pTet plasmids of strain 81-176) using *segemehl* (see Methods), the number of mappings (i.e. some reads map to different locations with the same score), and the number of uniquely-mapped reads. For the number of mapped reads and number of uniquely mapped reads, the percentage values (relative to the total number of reads) are also listed.

	C. jejuni NCTC11168 Control coIP	*C. jejuni* NCTC11168 CsrA-3xFLAG coIP
Total number of reads	6,214,261	5,389,919
Failed size filter after clipping	144,133	113,458
Total number of mapped reads	5,933,127	5,164,774
Total number of mappings (NC_002163)	13,207,712	8,897,385
Uniquely mapped reads	2,079,985	3,103,694
% mappable reads	95.48	95.82
% of uniquely mapped reads	33.47	57.58

	C. jejuni 81-176 Control coIP	*C. jejuni* 81-176 CsrA-3xFLAG coIP
Total number of reads	6,053,715	4,605,355
Failed size filter after clipping	295,445	223,949
Total number of mapped reads	5,641,676	4,299,931
Total number of mappings	13,439,403	9,866,887
Uniquely mapped reads	1,545,585	1,385,653
% mappable reads	93.19	93.37
% of uniquely mapped reads	25.53	30.09
Mapped reads in plasmid pVir (NC_008770)	16,383	9,753
Mapped reads in chromosome (NC_008787)	5,601,153	4,268,583
Mapped reads in plasmid pTet (NC_008790)	24,140	21,595
Mappings in plasmid pVir (NC_008770)	17,832	10,780
Mappings in chromosome (NC_008787)	13,396,129	9,833,383
Mappings in plasmid pTet (NC_008790)	25,442	22,724

Supplementary Table 2. Distribution of mapped reads to annotations. The table indicates the total number of mapped reads overlapping annotations for different RNA classes (sRNAs, 5'UTRs, ORFs, rRNAs, tRNAs, housekeeping RNAs, and pseudogenes) with a minimum overlap size of 10 nt (for details see Methods). Absolute read numbers and percentage values based on the total number of reads overlapping all annotations are shown for all libraries.

	C. jejuni NCTC11168 Control coIP		*C. jejuni* NCTC11168 CsrA-3xFLAG coIP	
sRNAs	28,720.50	0.50%	25,507.28	0.51%
5'UTRs	23,806.78	0.41%	756,447.88	15.08%
ORFs	249,394.84	4.30%	1,092,168.37	21.77%
rRNAs	3,285,700.41	56.66%	1,593,895.71	31.78%
tRNAs	1,509,545.38	26.03%	1,191,322.49	23.75%
housekeeping RNAs	699,295.33	12.06%	351,895.93	7.02%
pseudogenes	2,893.90	0.05%	4,752.24	0.09%
total	5,799,357.14	100.00%	5,015,989.91	100.00%

	C. jejuni 81-176 Control CoIP		*C. jejuni* 81-176 CsrA-3xFLAG CoIP	
sRNAs	32,282.87	0.58%	22,503.67	0.53%
5'UTRs	23,432.45	0.42%	187,578.08	4.40%
ORFs	344,388.16	6.16%	509,091.48	11.94%
rRNAs	3,641,665.55	65.09%	2,630,055.93	61.69%
tRNAs	1,267,744.45	22.66%	698,004.19	16.37%
housekeeping RNAs	284,913.59	5.09%	215,899.56	5.06%
total	5,594,427.06	100.00%	4,263,132.91	100.00%

Supplementary Table 3. Bacterial strains. List of all *C. jejuni* and *E. coli* strains used in this study. All strains were generated in this study unless otherwise stated. All *C. jejuni* strains correspond to NCTC11168 background unless otherwise stated.

Name	Description	Strain number	Resistance
C. jejuni strains	All *C. jejuni* strains have NCTC11168 background unless otherwise stated		
NCTC11168	WT strain; (Kindly provided by Arnoud van Vliet, Institute of Food Research, Norwich, UK)	CSS-0032	-
81-176	WT strain; (Patricia Guerry, Naval Medical Research Center, Silver Spring, MD, USA)	CSS-0063	Tet^R
CsrA-3xFLAG NCTC11168	*csrA*-3xFLAG::*aphA-3*<br>C-terminal 3xFLAG tag at native locus (Cj1103) in NCTC11168 background	CSS-0625	Kan^R
CsrA-3xFLAG 81-176	81-176, C-terminal 3xFLAG tag in 81-176 background	CSS-0604	Tet^R Kan^R
**Δ*csrA***	*csrA*::*cat*, Deletion of *csrA* (Cj1103)	CSS-0643	Cm^R
**Δ*fliW***	*fliW*::*aac*(3)-IV<br>Deletion of *fliW* (Cj1075)	CSS-0820	Gm^R
**Δ*csrA* Δ*fliW***	*csrA*::*cat; fliW*::*aac(3)-IV*<br>Deletion of *csrA* and *fliW*	CSS-1134	Cm^R Gm^R
**Δ*rpoN***	*rpoN*::*aac*(3)-IV<br>Deletion of *rpoN* (Cj0670)	CSS-1141	Gm^R
**Δ*fliA***	*fliA*::*aac*(3)-IV<br>Deletion of *fliA* (Cj0061c)	CSS-1133	Gm^R
**Δ*flaA***	*flaA*::*aphA-3*<br>Deletion of *flaA* (Cj1339c)	CSS1512	Kan^R
**Δ*flaB***	*flaB*::*aphA-3*<br>Deletion of *flaB* (Cj1338c)	CSS-2892	Kan^R
**Δ*flaA* Δ*flaB***	*flaAB*::*aphA-3*<br>Deletion of *flaA* (Cj1339c) and *flaB* (Cj1338c)	CSS-2891	Kan^R
flaA-3xFLAG	*flaA*-3xFLAG::*aphA-3*<br>C-terminal 3xFLAG tag of *flaA* at native locus (Cj1339c)	CSS-0640	Kan^R
flaA-3xFLAG **Δ*csrA***	*flaA*-3xFLAG::*aphA-3; csrA*::*cat*<br>Deletion of *csrA* in *flaA*-3xFLAG background	CSS-0644	Kan^R Cm^R
flaA-3xFLAG **Δ*fliW***	*flaA*-3xFLAG::*aphA-3; fliW*::*aac*<br>Deletion of *fliW* in *flaA*-3xFLAG background	CSS-1100	Gm^R Kan^R
flaA-3xFLAG **Δ*csrA* Δ*fliW***	*flaA*-3xFLAG::*aphA-3; csrA*::*cat; fliW*::*aac*(3)-IV<br>Deletion of *csrA* and *fliW* in *flaA*-3xFLAG background	CSS-1107	Kan^R Cm^R Gm^R
FlaA-3xFLAG M1	*flaA*[5'UTR SL1$^{GGA\rightarrow AAA}$::*aac*(3)-IV]-3xFLAG::*aphA-3*<br>SL1^{flaA} 5'UTR point mutant in *flaA*-3xFLAG background	CSS-0991	Kan^R Gm^R
FlaA-3xFLAG M1 **Δ*csrA***	*flaA*[5'UTR SL1$^{GGA\rightarrow AAA}$::*aac*(3)-IV]-3xFLAG::*aphA-3; csrA*::*cat*<br>SL1^{flaA} point mutant and *csrA* deletion in *flaA*-3xFLAG background	CSS-1410	Kan^R Cm^R Gm^R
FlaA-3xFLAG M1 **Δ*fliW***	*flaA*[5'UTR SL1$^{GGA\rightarrow AAA}$::*cat*]-3xFLAG::*aphA-3; fliW*::*aac*(3)-IV<br>Deletion of *fliW* in *flaA* 5'UTR point mutant/3xFLAG-tag	CSS-1418	Kan^R Cm^R Gm^R
FlaA-3xFLAG M1 **Δ*fliW* Δ*csrA***	*flaA*-3xFLAG::*aphA-3; flaA*[5'UTR SL1$^{GGA\rightarrow AAA}$::*cat*]; *fliW*::*aac*(3)-IV *csrA*:: *aph(7")*<br>SL1^{flaA} point mutant, *fliW* deletion and *csrA* deletion in *flaA*-3xFLAG background	CSS-1554	Kan^R Cm^R Gm^R Hyg^R
FlaA-3xFLAG M2	*flaA*[5'UTR SL1$^{GGA\rightarrow UGA}$::*aac*(3)-IV]-3xFLAG::*aphA-3*<br>SL1^{flaA} point mutant in *flaA*-3xFLAG background	CSS-0955	Kan^R Gm^R
FlaA-3xFLAG M2 **Δ*csrA***	*flaA*[5'UTR SL1$^{GGA\rightarrow UGA}$::*aac*(3)-IV]-3xFLAG::*aphA-3 csrA*::*cat*<br>SL1^{flaA} point mutant and *csrA* deletion in *flaA*-3xFLAG background	CSS-1105	Kan^R Cm^R Gm^R
FlaA-3xFLAG M2/M3	*flaA*[5'UTR SL1$^{GGA\rightarrow UGA}$ SL2$^{GGA\rightarrow GGG}$::*aac*(3)-IV]-3xFLAG::*aphA-3*<br>SL1^{flaA} and SL2^{flaA} double point mutant in *flaA*-3xFLAG background	CSS-1095	Kan^R Gm^R
FlaA-3xFLAG M2/M3 **Δ*csrA***	*flaA*[5'UTR SL1$^{GGA\rightarrow UGA}$ SL2$^{GGA\rightarrow GGG}$::*aac*(3)-IV]-3xFLAG::*aphA-3* *csrA*::*cat*<br>SL1^{flaA} and SL2^{flaA} double point mutant and *csrA* deletion in *flaA*-3xFLAG background	CSS-1106	Kan^R Cm^R Gm^R
FlaG-3xFLAG	*flaG*-3xFLAG::*aphA-3*;<br>C-terminal 3xFLAG-tag of *flaG* at native locus (Cj0547)	CSS-0968	Kan^R

FlaG-3xFLAG<br>**Δ*csrA***	*flaG*-3xFLAG::*aphA-3*; *csrA*::*cat*<br>Deletion of *csrA* in *flaG*-3xFLAG background	CSS-0983	Kan^R Cm^R
FlaG-3xFLAG<br>**Δ*fliW***	*flaG*-3xFLAG::*aphA-3*; *fliW*::*aac*(3)-IV<br>Deletion of *fliW* in FlaG-3xFLAG background	CSS-1112	Kan^R Gm^R
FlaG-3xFLAG<br>**Δ*csrA* Δ*fliW***	*flaG*-3xFLAG::*aphA-3; csrA*::*cat; fliW*::*aac*(3)-IV<br>Deletion of *csrA* and *fliW* in FlaG-3xFLAG background	CSS-1204	Kan^R Cm^R<br>Gm^R
FlaG-3xFLAG M1	*flaG*::3xFLAG::*aphA-3; flaA*[5'UTR SL1$^{GGA \rightarrow AAA}$::*aac*(3)-IV]<br>SL1^{flaA} point mutant in *flaG*-3xFLAG background	CSS-1399	Kan^R Gm^R
FlaG-3xFLAG M1<br>**Δ*csrA***	*flaG*-3xFLAG::*aphA-3; flaA*[5'UTR SL1$^{GGA \rightarrow AAA}$::*aac*(3)-IV] *csrA*::*cat*<br>SL1^{flaA} point mutant and *csrA* deletion in *flaG*-3xFLAG background	CSS-1411	Kan^R Cm^R<br>Gm^R
FlaG-3xFLAG M1<br>**Δ*fliW***	*flaG*-3xFLAG::*aphA-3; flaA*[5'UTR SL1$^{GGA \rightarrow AAA}$::*cat*] *fliW*::*aac*(3)-IV<br>SL1^{flaA} point mutant and *fliW* deletion in *flaG*-3xFLAG background	CSS-1421	Kan^R Cm^R<br>Gm^R
FlaG-3xFLAG M1<br>**Δ*fliW* Δ*csrA***	*flaG*-3xFLAG::*aphA-3; flaA*[5'UTR SL1$^{GGA \rightarrow AAA}$::*cat*]*; fliW*::*aac*(3)-IV *csrA*::<br>*aph(7");* SL1^{flaA} point mutant, *fliW* deletion and *csrA* deletion in *flaG*-<br>3xFLAG background	CSS-1435	Kan^R Cm^R<br>Gm^R Hyg^R
FlgI-3xFLAG	*flgI*-3xFLAG::*aphA-3*<br>C-terminal 3xFLAG tag of *flgI* at native locus (Cj1462)	CSS-0967	Kan^R
FlgI-3xFLAG<br>**Δ*csrA***	*flgI*-3xFLAG::*aphA-3; csrA*::*cat*<br>Deletion of *csrA* in *flgI*-3xFLAG background	CSS-0982	Kan^R Cm^R
FlgI-**3xFLAG Δ*fliW***	*flgI*-3xFLAG::*aphA-3; fliW*::*aac*(3)-IV<br>Deletion of *fliW* in *flgI*-3xFLAG background	CSS-1114	Kan^R Gm^R
FlgI-3xFLAG<br>**Δ*csrA* Δ*fliW***	*flgI*-3xFLAG::*aphA-3; csrA*::*cat; fliW*::*aac*(3)-IV<br>Deletion of *csrA* and *fliW* in *flgI*-3xFLAG background	CSS-1203	Kan^R Cm^R<br>Gm^R
FlgI-3xFLAG M1	*flgI*-3xFLAG::*aphA-3; flaA*[5'UTR SL1$^{GGA \rightarrow AAA}$::*aac*(3)-IV]<br>SL1^{flaA} point mutant in *flgI*-3xFLAG background	CSS-1426	Kan^R Gm^R
FlgI-3xFLAG M1<br>**Δ*csrA***	*flgI*-3xFLAG::*aphA-3; flaA*[5'UTR SL1$^{GGA \rightarrow AAA}$::*aac*(3)-IV] *csrA*::*cat*<br>SL1^{flaA} point mutant and *csrA* deletion in *flgI*-3xFLAG background	CSS-1542	Kan^R Cm^R<br>Gm^R
FlgI-3xFLAG M1<br>**Δ*fliW***	*flgI*-3xFLAG::*aphA-3; flaA*[5'UTR SL1$^{GGA \rightarrow AAA}$::*cat*] *fliW*::*aac*(3)-IV<br>SL1^{flaA} point mutant and *fliW* deletion in *flgI*-3xFLAG background	CSS-1420	Kan^R Cm^R<br>Gm^R
FlgI-3xFLAG M1<br>**Δ*fliW* Δ*csrA***	*flgI*-3xFLAG::*aphA-3; flaA*[5'UTR SL1$^{GGA \rightarrow AAA}$::*cat*]*; fliW*::*aac*(3)-IV *csrA*::<br>*aph(7")*<br>SL1^{flaA} point mutant, *fliW* deletion and *csrA* deletion in *flgI*-3xFLAG<br>background	CSS-1436	Kan^R Cm^R<br>Gm^R Hyg^R
FlaB-3xFLAG	*flaB*-3xFLAG::*aphA-3;*<br>C-terminal 3xFLAG tag of *flaB* at native locus (Cj1338c)	CSS-0641	Kan^R
FlaB-3xFLAG<br>**Δ*csrA***	*flaB*-3xFLAG::*aphA-3; csrA*::*cat*<br>Deletion of *csrA* in *flaB*-3xFLAG background	CSS-0645	Kan^R Cm^R
FlaB-3xFLAG<br>**Δ*fliW***	*flaB*-3xFLAG::*aphA-3; fliW*::*aac*(3)-IV<br>Deletion of *fliW* in *flaB*-3xFLAG background	CSS-1201	Kan^R Gm^R
FlaB-3xFLAG<br>**Δ*csrA* Δ*fliW***	*flaB*-3xFLAG::*aphA-3; csrA*::*cat; fliW*::*aac*(3)-IV<br>Deletion of *csrA* and *fliW* in *flaB*-3xFLAG background	CSS-1202	Kan^R Cm^R<br>Gm^R
FlaB-3xFLAG M1	*flaB*-3xFLAG::*aphA-3; flaA*[5'UTR SL1$^{GGA \rightarrow AAA}$::*aac*(3)-IV]<br>SL1^{flaA} point mutant in *flaB*-3xFLAG background	CSS-1405	Kan^R Gm^R
FlaB-3xFLAG M1<br>**Δ*csrA***	*flaB*-3xFLAG::*aphA-3; flaA*[5'UTR SL1$^{GGA \rightarrow AAA}$::*aac*(3)-IV]<br>SL1^{flaA} point mutant and *csrA* deletion in *flaB*-3xFLAG background	CSS-1543	Kan^R Cm^R<br>Gm^R
FlaB-3xFLAG M1<br>**Δ*fliW***	*flaB*-3xFLAG::*aphA-3; flaA*[5'UTR SL1$^{GGA \rightarrow AAA}$::*cat*] *fliW*::*aac*(3)-IV<br>SL1^{flaA} point mutant and *fliW* deletion in *flaB*-3xFLAG background	CSS-1419	Kan^R Cm^R<br>Gm^R
FlaB-3xFLAG M1<br>**Δ*fliW* Δ*csrA***	*flaB*-3xFLAG::*aphA-3; flaA*[5'UTR SL1$^{GGA \rightarrow AAA}$::*cat*]*; fliW*::*aac*(3)-IV *csrA*::<br>*aph(7")*<br>SL1^{flaA} point mutant, *fliW* deletion and *csrA* deletion in *flaB*-3xFLAG<br>background	CSS-1438	Kan^R Cm^R<br>Gm^R Hyg^R
Cj0529-3xFLAG	Cj0529-3xFLAG::*aphA-3*<br>C-terminal 3xFLAG tag of Cj0529 at native locus	CSS-1541	Kan^R
Cj0529-3xFLAG<br>**Δ*csrA***	Cj0529-3xFLAG::*aphA-3; csrA*::*cat*<br>Deletion of *csrA* in Cj0529-3xFLAG background	CSS-1431	Kan^R Cm^R
Cj0529-3xFLAG<br>**Δ*fliW***	Cj0529-3xFLAG::*aphA-3; fliW*::*aac*(3)-IV<br>Deletion of *fliW* in Cj0529-3xFLAG background	CSS-1430	Kan^R Gm^R
Cj0529-3xFLAG<br>**Δ*csrA* Δ*fliW***	Cj0529-3xFLAG::*aphA-3; csrA*::*cat; fliW*::*aac*(3)-IV<br>Deletion of *csrA* and *fliW* in Cj0529-3xFLAG background	CSS-1437	Kan^R Cm^R<br>Gm^R
Cj0529-3xFLAG<br>M1	Cj0529-3xFLAG::*aphA-3; flaA*[5'UTR SL1$^{GGA \rightarrow AAA}$::*aac*(3)-IV]<br>SL1^{flaA} point mutant in Cj0529-3xFLAG background	CSS-1432	Kan^R Gm^R
Cj0529-3xFLAG<br>**M1 Δ*csrA***	Cj0529-3xFLAG::*aphA-3; flaA*[5'UTR SL1$^{GGA \rightarrow AAA}$::*aac*(3)-IV]; *csrA*::*cat*<br>SL1^{flaA} point mutant and *csrA* deletion in Cj0529-3xFLAG background	CSS-1576	Kan^R Cm^R<br>Gm^R

Cj0529-3xFLAG<br>**M1 Δ*fliW***	Cj0529-3xFLAG::*aphA-3; flaA*[5'UTR SL1$^{GGA\rightarrow AAA}$::*cat*]*; fliW*::*aac*(3)-IV<br>SL1^{flaA} point mutant and *fliW* deletion in Cj0529-3xFLAG background	CSS-1575	Kan^R Cm^R<br>Gm^R
Cj0529-3xFLAG<br>M1 **Δ*fliW* Δ*csrA***	Cj0529-3xFLAG::*aphA-3; flaA*[5'UTR SL1$^{GGA\rightarrow AAA}$::*cat*]*; fliW*::*aac*(3)-IV<br>*csrA:: aph(7")*<br>SL1^{flaA} point mutant, *fliW* deletion and *csrA* deletion in Cj0529-3xFLAG background	CSS-1582	Kan^R Cm^R<br>Gm^R Hyg^R
flaA X1	*flaA*[Start Codon$^{AUG\rightarrow AAG}$::*aac*(3)-IV]	CSS-1586	Gm^R
flaA X2	*flaA*[Start Codon$^{AUG\rightarrow AUU}$::*aac*(3)-IV]	CSS-3089	Gm^R
flaA X3	*flaA*[Start Codon$^{AUG\rightarrow GUG}$::*aac*(3)-IV]	CSS-3087	Gm^R
flaA X4	*flaA*[3rd Codon$^{UUU\rightarrow UAG}$::*aac*(3)-IV]	CSS-3091	Gm^R
flaA X5	*flaA*[3rd Codon$^{UUU\rightarrow UUC}$::*aac*(3)-IV]	CSS-3093	Gm^R
flaA X6	*flaA*[101st Codon$^{CAA\rightarrow UAA}$::*aac*(3)-IV]	CSS-3095	Gm^R
P_{MetK}-*flaA*	Exchange of *flaA* native promoter with *metK* promoter	CSS-3096	Gm^R
P_{MetK}-*flaA*-3xFLAG	*flaA*-3xFLAG::*aphA-3*; P_{MetK}-*flaA:: aac*(3)-IV<br>C-terminal 3xFLAG tag of *flaA* in *flaA* promoter exchanged strain	CSS-3098	Gm^R Kan^R
P_{MetK}-*flaA*-3xFLAG<br>**Δ*csrA***	*flaA*-3xFLAG::*aphA-3*; P_{MetK}-*flaA:: aac*(3)-IV*; csrA*::*cat*<br>C-terminal 3xFLAG tag of *flaA* in *flaA* promoter exchanged strain	CSS-3102	Gm^R Kan^R<br>Cm^R
P_{MetK}-*flaA*-3xFLAG<br>**Δ*fliW***	*flaA*-3xFLAG::*aphA-3*; P_{MetK}-*flaA:: aac*(3)-IV*; fliW*::*hyg*<br>C-terminal 3xFLAG tag of *flaA* in *flaA* promoter exchanged strain	CSS-3104	Gm^R Kan^R<br>Hyg^R
P_{MetK}-*flaA*-3xFLAG<br>**Δ*csrA* Δ*fliW***	*flaA*-3xFLAG::*aphA-3*; P_{MetK}-*flaA:: aac*(3)-IV; *csrA*::*cat; fliW*::*aph(7")*<br>C-terminal 3xFLAG tag of *flaA* in *flaA* promoter-exchanged strain	CSS-3124	Gm^R Kan^R<br>Cm^R Hyg^R
FliW-3xFLAG	*fliW*-3xFLAG::*aac*(3)-IV<br>C-terminal 3xFLAG tag of *fliW* at native locus (Cj1075)	CSS-0962	Gm^R
FliW-mCherry	*fliW*-mCherry::*aac*(3)-IV<br>C-terminal mCherry tag of *fliW* at native locus (Cj1075)	CSS-3073	Gm^R
CsrA-mCherry	*csrA*-mCherry::*aphA-3*<br>C-terminal mCherry tag of *csrA* at native locus (Cj1103)	CSS-3071	Kan^R
FliW-3xFLAG<br>CsrA-mCherry	*fliW*-3xFLAG::*aac*(3)-IV<br>*csrA*-mCherry::*aphA-3*	CSS-3126	Gm^R Kan^R
FlaA-3xFLAG<br>FliW-mCherry	*flaA*-3xFLAG::*aphA-3*<br>*fliW*-mCherry::*aac*(3)-IV	CSS-3128	Gm^R Kan^R
*flaA*_mini	*flaA*_mini::*rdxA*<br>Introduction of *flaA_mini* in *rdxA* (Cj1066) complementation locus	CSS-3075	Cm^R
FlaA-3xFLAG<br>*flaA*_mini	*flaA*-3xFLAG::*aphA-3*; *flaA_mini*::*rdxA*<br>Introduction of *flaA*_mini in *rdxA* (Cj1066) in *flaA*-3xFLAG background	CSS-3076	Cm^R Kan^R
FlaA-3xFLAG<br>**Δ*fliW***<br>*flaA*_mini	*flaA*-3xFLAG::*aphA-3*; *fliW*::*aac*(3)-IV*; flaA_mini*::*rdxA*<br>Introduction of *flaA_mini* in *rdxA* (Cj1066) in *flaA*-3xFLAG background with *fliW* deletion	CSS-3106	Cm^R Kan^R<br>Gm^R
FlaG-3xFLAG<br>*flaA*_mini	*flaG*-3xFLAG::*aphA-3*; *flaA_mini*::*rdxA*<br>Introduction of *flaA_mini* in *rdxA* (Cj1066) in *flaG*-3xFLAG background	CSS-3080	Cm^R Kan^R
FlaG-3xFLAG<br>**Δ*fliW***<br>*flaA*_mini	*flaG*-3xFLAG::*aphA-3*; *fliW*::*aac*(3)-IV*; flaA_mini*::*rdxA*<br>Introduction of *flaA_mini* in *rdxA* (Cj1066) in *flaG*-3xFLAG background with *fliW* deletion	CSS-3112	Cm^R Kan^R<br>Gm^R
Cj1321_mini	Cj1321_mini::*rdxA*<br>Introduction of *flaA_mini* in *rdxA* (Cj1066) complementation locus	CSS-3130	Cm^R
Cj1321_mini<br>**Δ*csrA***	Cj1321_mini::*rdxA csrA*::*aph(7")*<br>Introduction of *flaA_mini* in *rdxA* (Cj1066) complementation locus	CSS-3131	Cm^R Hyg^R
Cj1321_mini<br>**Δ*fliW***	Cj1321_mini::*rdxA fliW*::*aac*(3)-IV<br>Introduction of *flaA_mini* in *rdxA* (Cj1066) complementation locus	CSS-3132	Cm^R Gm^R
E. coli strains			
TOP10	*mcrA* Δ(*mrr-hsdRMS-mcrBC*) Φ80*lacZ* ΔM15 Δ*lacX74 deoR recA1 araD139* Δ(*ara-leu*)7697 *galU galK rpsL* endA1 nupG (from Invitrogen)	CSS-0070	Str^R
**Δ*pgaA***	*pgaA* deletion in TOP10 background	CSS-0556	Str^R
**Δ*pgaA* Δ*csrA***	*pgaA* and *csrA* deletion in TOP10 background	CSS-0557	Str^R Kan^R

Supplementary Table 4. DNA oligonucleotides. List of all DNA oligonucleotides used in this study for PCR amplification, Northern blot hybridization, and FISH assays. DNA sequences are given in 5' to 3' direction; P- denotes a 5' monophosphate.

Name	Sequence (5' → 3')	Description
CSO-0023	CCACCAGCTTATATACCTTAGCA	Antisense to *aphA-3* for verification
CSO-0073	CTAACAAGCTTTCATCTACGCA	3xFLAG Tagging using pGG1
CSO-0074	GTTTTTGAATTCTATTCCCTCCAGGTACTAAAACA	3xFLAG Tagging using pGG1
CSO-0075	TCCTTCACAAAGAAGGGG	3xFLAG Tagging using pGG1
CSO-0171	P-TTTGATTAGTTTTTTGCTTAAGTCAT	Cloning of *csrA*-3xFLAG in pGG1
CSO-0172	GTTTTTCTCGAGCTCTTTAGAGCGCATTAAAGAA	Cloning of *csrA* -3xFLAG in pGG1
CSO-0173	GTTTTTTCTAGACAAGATATTTGTGGAAAAGTCC	Cloning of *csrA*-3xFLAG in pGG1
CSO-0174	GTTTTTGAATTCATCAAATGAAAGCTTACGCTAA	Cloning of *csrA*-3xFLAG in pGG1
CSO-0196	GTATTTGATTGCAAGATCTTAAGC	Verification of *csrA*-3xFLAG in *C. jejuni*
CSO-0392	TACTCCTTAAGTCTTGATGATCAA	Verification of *csrA* deletion in *C. jejuni*
CSO-0393	TCCTAGTTAGTCACCCGGGTACCTTGATAATATTAACAT TTTTCAACCT	Deletion of *csrA* using *hyg* in *C. jejuni*
CSO-0394	TGCAAGGAATTATCTCCTATACAC	Deletion of *csrA* using *hyg/cat* in *C. jejuni*
CSO-0395	ATCATAAACAGCTTTAGTTTGGC	Deletion of *csrA* using *hyg/cat* in *C. jejuni*
CSO-0396	ATTGTTTTAGTACCTGGAGGGAATAGCAAAAAACTAATC AAATGAAAG	Deletion of *csrA* using *hyg* in *C. jejuni*
CSO-0483	GTTTTTGGATCCTTTTATGGATAATTTTTAAAATCATTTG	Cloning of *aac*(3)-IV upstream of *flaA* 5'UTR
CSO-0486	GTGTTAATACGAAATCCCATTTTAAATC	NB detection *flaA* mRNA (Binds 5'UTR)
CSO-0553	P-CTGTAGTAATCTTAAAACATTTTGTTGA	Cloning of *flaA*-3xFLAG in pGG1
CSO-0554	GTTTTTCTCGAGTGGTTATTCTTCTGTTAGTGCCT	Cloning of *flaA*-3xFLAG in pGG1
CSO-0555	GTTTTTTCTAGAGCGATATTGTCAAGTTCTTCC	Cloning of *flaA*-3xFLAG in pGG1
CSO-0556	GTTTTTGAATTCTTTACAAAAGCTGCAATATATACAAA	Cloning of *flaA*-3xFLAG in pGG1
CSO-0557	CTCTCAAGCTTCTGTTTCTTTAAG	Verification of *flaA*-3xFLAG in *C. jejuni*
CSO-0558	P-TTGAAGAAGTTTTAAAACATTTTGC	Cloning of *flaB*-3xFLAG in pGG1
CSO-0559	GTTTTTCTCGAGTTAGTGCCTATATGAGTAGCGC	Cloning of *flaB*-3xFLAG in pGG1
CSO-0560	GTTTTTTCTAGAGTGCTAGGATAGAAAGCGCT	Cloning of *flaB*-3xFLAG in pGG1 / Overlap PCR construction of *flaB* deletion with *aphA-3*
CSO-0561	GTTTTTGAATTCTTTCTTAGATGCTTTTATGCATCT	Cloning of *flaB*-3xFLAG in pGG1
CSO-0562	GATGCTAATATCGCTGATGC	Verification of *flaB*-3xFLAG
CSO-0575	CAATACGAATGGCGAAAAG	*aac*(3)-IV cloning in pGG1
CSO-0576	GTTTTTCATATGAAACACCCCCATAAGTGCAATTATGGG GATAAATCATCTCGTTCTCCGCTC	Cloning of *aac*(3)-IV upstream of *flaA* 5'UTR
CSO-0577	P-CATTTATTCCTCCTAGTTAGTCACC	*aac*(3)-IV cloning in pGG1
CSO-0606	GTTTTTATGCATTTTATTCAAGAAAATTCAACTACGG	Cj0805-Cj0806 (*dapA*) cloning in pXG-30
CSO-0607	GTTTTTGCTAGCTTGCTCATCAACTTTTCCAT	Cj0805-Cj0806 (*dapA*) cloning in pXG-30
CSO-0608	GTTTTTATGCATGCAATTTTACTTTTAAGTATTATAGCCC	Cj0310c-Cj0309c cloning in pXG-30
CSO-0609	GTTTTTGCTAGCAAGTTCTTTCATGATCACCACG	Cj0310c-Cj0309c cloning in pXG-30
CSO-0611	TACAGAGAGACCCGACTCTTTTAATCTTTCAAGGAGCAA AGAATGGTGTAGGCTGGAGCTGCTTC	Deletion of *csrA* in *E. coli* (using λ-red system)
CSO-0612	TTTGAGGGTGCGTCTCACCGATAAAGATGAGACGCGGA AAGATTAGGTCCATATGAATATCCTCCTTAG	Deletion of *csrA* in *E. coli* (using λ-red system)
CSO-0613	AACAAATCGGAATTTACGGA	Amplification of *C. coli cat* cassette
CSO-0614	GGCACCAATAACTGCCTTAA	Amplification of *C. coli cat* cassette

CSO-0615	CTCCGTAAATTCCGATTTGTTCTTGATAATATTAACATTTTTCAACCT	Deletion of *csrA* using *cat* in *C. jejuni*
CSO-0616	TTTTAAGGCAGTTATTGGTGCCGCAAAAAACTAATCAAATGAAAG	Deletion of *csrA* using *cat* in *C. jejuni*
CSO-0621	GTTTTTATGCATTAACAAGTTCATGGATGAGCTT	*flaA* cloning in pXG-10
CSO-0622	GTTTTTGCTAGCACTAAGTCTGCTTAAAGAAGCATC	*flaA* cloning in pXG-10
CSO-0639	GATGTAATGTGTTTGTCATTGCT	Verification of *csrA* deletion in *E. coli*
CSO-0640	GAGACTTAAGTTGAATGAACGG	Verification of *csrA* deletion in *E. coli*
CSO-0652	AGATACAGAGAGAGATTTTGGCAATACATGGAGTAATACAGGATGGTGTAGGCTGGAGCTGCTTC	Deletion of *pgaA* in *E.coli* (using λ-red system)
CSO-0653	GCATCAGGAGATATTTATTTCCATTACGTAACATATTTATCCTTAGGTCCATATGAATATCCTCCTTAG	Deletion of *pgaA* in *E.coli* (using λ-red system)
CSO-0654	TCTCTCTTCCGCGTTTAATAAC	Verification of *pgaA* deletion in *E.coli*
CSO-0655	CTGTGGCGGTATAAATGATG	Verification of *pgaA* deletion in *E.coli*
CSO-0694	GTTTTTATGCATACAATAGATTAAAGGAAGAATCCAT	*flgI* cloning in pXG-10
CSO-0695	GTTTTTGCTAGCACCTATAAGTTGGTTATCTCTTACACC	*flgI* cloning in pXG-10
CSO-0701	GTTTTTTTTAATACGACTCACTATAGGAAAGCTGGTGGCGCTG	*in vitro* transcription of *hopB 3*'end, carries T7 promoter
CSO-0702	GTTAAATCAAAGCCTATAAAAGGCC	*in vitro* transcription of *hopB 3*'end
CSO-0709	GTTTTTTTTAATACGACTCACTATAGGTAACAAGTTCATGGATGAGCTT	*in vitro* transcription of *flaA* leader, carries T7 promoter
CSO-0710	ACTAAGTCTGCTTAAAGAAGCATCT	*in vitro* transcription of *flaA* leader
CSO-0713	GTTTTTTTTAATACGACTCACTATAGGACAATAGATTAAAGGAAGAATCCAT	*in vitro* transcription of *flgI* leader, carries T7 promoter
CSO-0714	ACCTATAAGTTGGTTATCTCTTACACC	*in vitro* transcription of *flgI* leader
CSO-0748	P-TAACAAGTTCATGGATGAGCTT	*flaA* leader cloning in pBAD plasmid
CSO-0749	GTTTTTTCTAGAGTTTGCTTTTGCATTTAAAGCT	*flaA* leader cloning in pBAD plasmid
CSO-0752	GTTTTTCTCGAGAAGGTGGAGCAAGGATTAA	*flaA* cloning in pJV752.1 / Overlap PCR construction of *flaA* deletion with *aphA-3*
CSO-0753	GTTTTTTCTAGATCTTAGAAGATTGAGTTGCTCC	*flaA* cloning in pJV752.1
CSO-0754	GTTTTTCATATGCAATAAAATTTCATACTTTTGACA	Cloning of *aac*(3)-IV upstream of *flaA* 5'UTR
CSO-0755	GTTTTTGGATCCTAAAGTATAAAATATTTTTTTGATTGCA	Cloning of *aac*(3)-IV upstream of *flaA* 5'UTR
CSO-0756	TATGCAGGCAAAGGTGAAG	Verification of *flaA* deletion in *C. jejuni*
CSO-0757	TAACAAGTTCATTGATGAGCTTGAATTTTTTTAAAAG	Introduction of $SL1^{GGA>UGA}$ (M2) mutation into *flaA* 5' UTR
CSO-0758	TTCAAGCTCATCAATGAACTTGTTAAATGCTATATCGT	Introduction of $SL1^{GGA>UGA}$ (M2) mutation into *flaA* 5' UTR
CSO-0831	GTTTTTCATATGTGTTTTAGTACCTGGAGGGAATA	*aac*(*3*)-*IV* cloning in pGG1
CSO-0832	GTTTTTCATATGTCATCTCGTTCTCCGCTC	*aac*(*3*)-*IV* cloning in pGG1
CSO-0852	CGGGTGGCTCCATTTGATTAGTTTTTTGCTTAAGT	Addition of Strep-tag to *csrA* in pBAD plasmid
CSO-0853	P-CAGTTCGAAAAATGAAAGCTTACGCTCTAGA	Addition of Strep-tag to *csrA* in pBAD plasmid
CSO-0997	GATAACGAATATAATCAGCATTGC	Deletion of *fliW* using *aac*(3)-IV in *C. jejuni*
CSO-0998	TCCTAGTTAGTCACCCGGGTACGCATTTCAGCTAGGGTCATG	Deletion of *fliW* using *aac*(3)-IV in *C. jejuni*
CSO-0999	TGTGTTTTAGTACCTGGAGGGAATACCGACTTTTTTCAAGCTGATC	Deletion of *fliW* using *aac*(3)-IV in *C. jejuni*
CSO-1000	GACAAACCTTCATAAACTCCAG	Deletion of *fliW* using *aac*(3)-IV in *C. jejuni*
CSO-1002	GTTTTTCTCGAGAAATTTTGGCACAGTTTTTGCTTA	Overlap PCR construction of *flaG*-3xFLAG with *aphA-3* cassette
CSO-1003	GTTTTTTCTAGACCTGTGTTTACAATCTTAGCAAC	Overlap PCR construction of *flaG*-3xFLAG with *aphA -3* cassette
CSO-1005	GTGATAGAAGATTTGATCTTGC	Verification of *flaG*-3xFLAG in *C. jejuni*
CSO-1011	P-GATGATCTCCAAATCCGCGT	Cloning of *flgI*-3xFLAG in pGG1
CSO-1012	GTTTTTCTCGAGAAATTCCACAAAATTTTAGCC	Cloning of *flgI*-3xFLAG in pGG1

CSO-1013	GTTTTTTCTAGATGCGATTTTACTCGCTTTATCA	Cloning of *flgI*-3xFLAG in pGG1
CSO-1014	GTTTTTGAATTCACGCGGATTTGGAGATCATC	Cloning of *flgI*-3xFLAG in pGG1
CSO-1015	AACTGTAATGGGCGGAGCTA	Verification of *flgI*-3xFLAG in *C. jejuni*
CSO-1072	TAAAGCCTGATTACGATTTGGC	Verification of *fliW* deletion in *C. jejuni*
CSO-1081	GTTTTTTTTAATACGACTCACTATAGGTAACAAGTTCATT GATGAGCTTG	*in vitro* transcription of *flaA* M1/M2 variant, carries T7 promoter
CSO-1082	GTTTTTTTTAATACGACTCACTATAGGAAATTAAAATTTTA AAAAGGAAGTTAAA	*in vitro* transcription of Cj0040, carries T7 promoter
CSO-1083	TCTAAAACTTGAAGCAAAACTTC	*in vitro* transcription of Cj0040
CSO-1084	GTTTTTTTTAATACGACTCACTATAGGACTAGCAATAGGA AATTTTAAAAAG	*in vitro* transcription of *flaG*, carries T7 promoter
CSO-1085	TGTGTCTCACTTGTTCTTTGG	*in vitro* transcription of *flaG*
CSO-1088	GTTTTTTTTAATACGACTCACTATAGGCAAATGTTGATGT TTTAATCGAA	*in vitro* transcription of *flgA*, carries T7 promoter
CSO-1089	TTTACCCACTACGATACCTTG	*in vitro transcription of flgA*
CSO-1092	GTTTTTTTTAATACGACTCACTATAGGATTATAACTAAGA TCAAGGAG	*in vitro* transcription of *flgM*, carries T7 promoter
CSO-1093	CTTTATCTATTCTATTTGTATTTAATG	*in vitro* transcription of *flgM*
CSO-1098	TCACCGTCATGGTCTTTGTAGTCACTCTCCTTATCAAATA TCATTCC	Overlap PCR construction of *flaG*-3xFLAG with *aphA -3* cassette
CSO-1099	ATTGTTTTAGTACCTGGAGGGGGAATGATATTTGATAAG GAGAGT	Overlap PCR construction of *flaG*-3xFLAG with *aphA -3* cassette
CSO-1114	TAACAAGTTCATAAATGAGCTTGAATTTTTTTAAAAGG	Introduction of $SL1^{GGA>AAA}$ (M1) mutation into *flaA* 5'UTR
CSO-1115	ATTCAAGCTCATTTATGAACTTGTTAAATGCTATATCG	Introduction of $SL1^{GGA>AAA}$ (M1) mutation into *flaA* 5'UTR
CSO-1116	TTTTTTAAAAGGGTTTAAAATGGGATTTCGTATTAACA	Introduction of $SL2^{GGA>GGG}$ (M3) mutation into *flaA* 5'UTR
CSO-1117	TCCCATTTTAAACCCTTTTAAAAAAATTCAAGCTCAT	Introduction of $SL2^{GGA>GGG}$ (M3) mutation into *flaA* 5'UTR
CSO-1138	GTTTTTCATATGTATAAAATATTTTTTTGATTGCACGATAT AGCATTTAACAAGTTCATGGATGAGCTT	Cloning of *flaA_mini* in *Campylobacter rdxA* complementation plasmid
CSO-1139	GTTTTTATCGATAAGGCCCAGTCTTTCGACT	Cloning of *flaA_mini* in *Campylobacter rdxA* complementation plasmid
CSO-1144	AGTGGAAAAGTTCTTTTAGACGG	Overlap PCR construction of *rpoN* deletion with *aac*(3)-IV
CSO-1145	TCCTAGTTAGTCACCCGGGTACCTTGGGTGATTTTTTGC TTTAACA	Overlap PCR construction of *rpoN* deletion with *aac*(3)-IV
CSO-1146	TGTGTTTTAGTACCTGGAGGGAATATCTATCAATCTATC AAACCCATTAC	Overlap PCR construction of *rpoN* deletion with *aac*(3)-IV
CSO-1147	CATTGGACGCTCAGGACG	Overlap PCR construction of *rpoN* deletion with *aac*(3)-IV
CSO-1148	AACAACTTTTTATATGATATGTGGAC	Verification of *rpoN* deletion in *C. jejuni*
CSO-1149	GAATTCTTAGGTCATTTAAGCGC	Overlap PCR construction of *fliA* deletion with *aac*(3)-IV
CSO-1150	TCCTAGTTAGTCACCCGGGTACTTTCTTTAGCATTTGTG CATAAGC	Overlap PCR construction of *fliA* deletion with *aac*(3)-IV
CSO-1151	TGTGTTTTAGTACCTGGAGGGAATATAAAAAACTTAGAG AAAGGCTAGTG	Overlap PCR construction of *fliA* deletion with *aac*(3)-IV
CSO-1152	GATAACAATCTCATTTTGAGATACG	Overlap PCR construction of *fliA* deletion with *aac*(3)-IV
CSO-1153	TGCAGATGCAAACATTAAAAATCC	Verification of *fliA* deletion in *C. jejuni*
CSO-1407	CAGCCAAACAACTTGGACTT	Verification of Cj0529c-3xFLAG in *C. jejuni*
CSO-1408	TCACCGTCATGGTCTTTGTAGTCTTTTCCTTTTTGTAAAT TTATGGCTT	Overlap PCR construction of Cj0529-3xFLAG with *aphA - 3* cassette
CSO-1409	GCTCCTTATGATGAAGGAGT	Overlap PCR construction of Cj0529-3xFLAG with *aphA - 3* cassette
CSO-1410	CTTTAACTTAATTTAGAGCTTGC	Overlap PCR construction of Cj0529-3xFLAG with *aphA - 3* cassette
CSO-1411	ATTGTTTTAGTACCTGGAGGGAATATTTTTGATATTTTTA TACAAAATAGTTAA	Overlap PCR construction of Cj0529-3xFLAG with *aphA - 3* cassette
CSO-1471	GTTTTTTTTAATACGACTCACTATAGGTAACAAGTTCATA AATGAGCTTGA	*in vitro* transcription of *flaA* 5'UTR M1 variant, carries T7 promoter
CSO-1548	TCCTAGTTAGTCACCCGGGTATTTAAATCCTTTTAAAAAA ATTCAAGCT	Overlap PCR construction of *flaA* deletion with *aphA-3*

CSO-1549	ATTGTTTTAGTACCTGGAGGGAATATTTACAAAAGCTGC AATATATACAAAT	Overlap PCR construction of *flaA* deletion with *aphA-3*
CSO-1550	ATAGCTTGACCTAAAGTGGCT	Overlap PCR construction of *flaA* deletion with *aphA-3*
CSO-1665	GTTTTTTTTAATACGACTCACTATAGGATTTTTATTAAATT GAAGGGGTGGG	*in vitro* transcription of Cj1324, carries T7 promoter
CSO-1666	TACCTTCTTTATCTTTTGTAAAATTAATAC	*in vitro* transcription of Cj1324
CSO-1678	GTACCCGGGTGACTAACTAGGGTGACTAACTAGGAGGA ATAAATG	Amplification of Hyg^{R} cassette
CSO-1679	TATTCCCTCCAGGTACTAAAACAGTCATATTCCCTCCAG GTATCA	Amplification of Hyg^{R} cassette
CSO-1815	GTTTTTATGCATCGATGCAATATTTTGAAAGGATT	*flaB* cloning in pXG-10
CSO-1816	GTTTTTGCTAGCACCTGAACTAAGTCTGCTTAAA	*flaB* cloning in pXG-10
CSO-1817	GTTTTTTTTAATACGACTCACTATAGGCGATGCAATATTT TGAAAGGATT	*in vitro* transcription of *flaB* leader, carries T7 promoter
CSO-1818	ACCTGAACTAAGTCTGCTTAAA	*in vitro* transcription of *flaB* leader
CSO-1819	TAGAAATTTCAAGGAAGAAATATGCATGGAAAAATAGCT ATTTATATGGATTCTACAGGACGTGGAACCG	Cj1249 cloning in pXG-10
CSO-1820	CTAGCGGTTCCACGTCCTGTAGAATCCATATAAATAGCT ATTTTTCCATGCATATTTCTTCCTTGAAATTTCTATGCA	Cj1249 cloning in pXG-10
CSO-1823	GTTTTTATGCATACTAGCAATAGGAAATTTTAAAAAG	*flaG* cloning in pXG-10
CSO-1824	GTTTTTGCTAGCCTGTGTCTCACTTGTTCTTTG	*flaG* cloning in pXG-10
CSO-1825	GTTTTTATGCATAAAAAACTTAAGCAAAGGAAGGC	*pseB* cloning in pXG-10
CSO-1826	GTTTTTGCTAGCTTCTAGCAAAACCTTAGTATAAGTT	*pseB* cloning in pXG-10
CSO-1895	[CY5] ATTGGTGTTAATACGAAATCCCATTT	FISH oligo 1 to detect *flaA* mRNA (Cy5-labeled 5' end)
CSO-1896	[CY5] AATTCAAGCTCATCCATGAACTTGT	FISH oligo 2 to detect *flaA* mRNA (Cy5-labeled 5' end)
CSO-1963	[CY5] CGTTTGCTTTTGCATTTAAAGCTG	FISH oligo 3 to detect *flaA* mRNA (Cy5-labeled 5' end)
CSO-1964	[CY5] CTTAAAGAAGCATCTAAACTTTTACTAT	FISH oligo 4 to detect *flaA* mRNA (Cy5-labeled 5' end)
CSO-2006	[FITC] GCTGCCTCCCGTAGGAGT	Universal FISH oligo to detect 16s rRNA (FITC- labeled 5' end)
CSO-2019	AAGGATTTAAAAAGGGATTTCGTATTAACACCAAT	Introduction of start codon$^{AUG>AAG}$ mutation into *flaA* 5'UTR
CSO-2020	ATACGAAATCCCTTTTTAAATCCTTTTAAAAAAATTCAAG C	Introduction of start codon$^{AUG>AAG}$ mutation into *flaA* 5'UTR
CSO-2023	[CY5] GAGCTAAGATATTTGCTTTAGAGTAG	FISH oligo 5 to detect *flaA* mRNA (Cy5-labeled 5' end)
CSO-2024	[CY5] TGCCATGGCATAAGAGCCGCT	FISH oligo 6 to detect *flaA* mRNA (Cy- labeled 5' end)
CSO-2150	GTGAGCAAGGGCGAGGA	Amplification of *mCherry* (2nd codon to stop)
CSO-2151	TTACTTGTACAGCTCGTCCAT	Amplification of *mCherry* (2nd codon to stop)
CSO-2155	CCTCCTCGCCCTTGCTCACTTTTTTAATATAATTAGCAAT TTGATCA	Overlap PCR construction of *fliW-mCherry* with *aac*(3)-IV cassette
CSO-2156	CCTCCTCGCCCTTGCTCACTTTGATTAGTTTTTTGCTTAA GTCAT	Overlap PCR construction of *csrA-mCherry* with *aphA -3* cassette
CSO-2746	GTAAAGCCACCCGCTCCTATG	NB oligo to detect Cj1321_mini
CSO-2809	[Cy5] CACGGATTTGCGATTCTGCTG	FISH oligo 7 to detect *flaA* mRNA (Cy5-labeled 5' end)
CSO-2810	[Cy5] GTGATGTTGTTTATAGTTGATGTAAC	FISH oligo 8 to detect *flaA* mRNA (Cy5-labeled 5' end)
CSO-2811	[Cy5] AACTAAGGCTCCATTAGCATCAC	FISH oligo 9 to detect *flaA* mRNA (Cy5 -labeled 5' end)
CSO-2812	[Cy5] GTAATCTACTTTACCGATTTTTACCC	FISH oligo 10 to detect *flaA* mRNA (Cy5-labeled 5' end)
CSO-2813	[Cy5] AGATCTTAAACTATCTGCTATCGC	FISH oligo 11 to detect *flaA* mRNA (Cy5-labeled 5' end)
CSO-2814	[Cy5] TAGATATAGCTTGACCTAAAGTATTAG	FISH oligo 12 to detect *flaA* mRNA (Cy5-labeled 5' end)
CSO-2815	[Cy5] TATTAGCATCAATTTGTCCTTTTGAC	FISH oligo 13 to detect *flaA* mRNA (Cy5 -labeled 5' end)
CSO-2816	[Cy5] GTAATCTTAAAACATTTTGTTGAACAGAA	FISH oligo 14 to detect *flaA* mRNA (Cy5-labeled 5' end)
CSO-2821	P-TTTGATAAGTTTATTTGGATACAATTGTGGTAACAAGT TCATGGATGAGCTT	Exchange of *flaA* promoter with *metK* promoter
CSO-2841	CTTCAGGTTCAGGTTATTCTG	Verification of *flaB* deletion in *C. jejuni*

CSO-2842	GGCTCAGGTTTTTCAAGTGG	Overlap PCR construction of *flaB* deletion with *aphA-3*
CSO-2843	TCCTAGTTAGTCACCCGGGTAATCCTAAAACCCATTTTAAATCCTT	Overlap PCR construction of *flaB* deletion with *aphA-3*
CSO-2844	ATTGTTTTAGTACCTGGAGGGAATACAGCAAAATGTTTTAAAACTTCTTC	Overlap PCR construction of *flaB* deletion with *aphA-3*
CSO-2853	GCTTGATAAATTAAAAATTTACTAAAATTAGGATCCTTTTATGGATAATTTTTAAA	Exchange of *flaA* promoter with *metK* promoter
CSO-2825	AAAGGATTTAAAGTGGGATTTCGTATTAACACCAA	Introduction of start codon$^{AUG>GUG}$ mutation into *flaA* 5' UTR
CSO-2826	TACGAAATCCCACTTTAAATCCTTTTAAAAAAATTCAAGC	Introduction of start codon$^{AUG>GUG}$ mutation into *flaA* 5' UTR
CSO-2827	AGGATTTAAAATTGGATTTCGTATTAACACCAATG	Introduction of start codon$^{AUG>AUU}$ mutation into *flaA* 5' UTR
CSO-2828	AATACGAAATCCAATTTTAAATCCTTTTAAAAAAATTCAAGC	Introduction of start codon$^{AUG>AUU}$ mutation into *flaA* 5' UTR
CSO-2829	TTAAAATGGGATAGCGTATTAACACCAATGTTGCAG	Introduction of 3rd codon$^{UUU>UAG}$ mutation into *flaA* 5' UTR
CSO-2830	GGTGTTAATACGCTATCCCATTTTAAATCCTTTTAAAAAAAT	Introduction of 3rd codon$^{UUU>UAG}$ mutation into *flaA* 5' UTR
CSO-2831	TTAAAATGGGATTCCGTATTAACACCAATGTTGCAG	Introduction of 3rd codon$^{UUU>UUC}$ mutation into *flaA* 5' UTR
CSO-2832	GGTGTTAATACGGAATCCCATTTTAAATCCTTTTAAAAAAAT	Introduction of 3rd codon$^{UUU>UUC}$ mutation into *flaA* 5' UTR
CSO-2833	ACTCAAGCGGCTTAAGATGGACAAAGTTTAAAAACAAG	Introduction of 101st codon$^{CAA>UAA}$ mutation into *flaA* 5' UTR
CSO-2834	TTTGTCCATCTTAAGCCGCTTGAGTTGCCTTA	Introduction of 101st codon$^{CAA>UAA}$ mutation into *flaA* 5' UTR
CSO-2835	GCTGCAACATTGGTGTTAATACG	NB oligo to detect *flaA* mRNA in all 5'UTR point mutants and *flaA_mini*
HPK1	GTACCCGGGTGACTAACTAGG	Amplification of *aphA-3* cassette
HPK2	TATTCCCTCCAGGTACTAAAACA	Amplification of *aphA-3* cassette
JVO-0054	GGGATCAAGCCTGATTG	Sense to *aphA-3* for verification
JVO-0900	GGAGAAACAGTAGAGAGTTGC	Antisense oligo for inverse PCR on pBAD/*Myc*-His A
JVO-0901	TTTTTTCTAGATTAAATCAGAACGCAGA	Sense oligo for inverse PCR on pBAD /*Myc*-His A
JVO-5142	GACTACAAAGACCATGACGG	Sense oligo to 3xFLAG tag
pBAD-FW	ATGCCATAGCATTTTTATCC	Verification of insert in pBAD/*Myc*-His A plasmid
pZE-A	GTGCCACCTGACGTCTAAGA	Verification of insert in pJV752.1

Supplementary Table 5. Plasmids. List of all plasmids used in this study.

Name	Description/Generation	Origin/ Marker	Reference
pJV752.1	Cloning vector, pZE12-*luc* with modified p15A origin	p15A mod/ Amp^R	[5]
pUC1813 *apra*	Carries *aac*(*3*)-IV gene	pBR322/ Gm^R	[6]
pAC1H	Carries *aph*(7") gene	ColE1/pBR 322/Hyg^R	[7]
pBAD/Myc-His A	pBAD expression plasmid	pBR322/ Amp^R	Invitrogen
pZE12-luc	General expression plasmid	ColE1/ Amp^R	[8]
pXG-10	Standard plasmid for directional cloning of a target mRNA as N-terminal translational fusion to GFP	pSC101*/ Cm^R	[3]
pXG-30	Plasmid for cloning operon fusions with the N-terminus of downstream gene fused to GFP and the C-terminus of upstream gene fused to a short artificial reading frame composed of a FLAG epitope and truncated *lacZ* gene	pSC101*/ Cm^R	[3]
pGD68-1	pBAD::CsrA$_{Cj}$, based on pBAD/Myc-His A	pBR322/ Amp^R	This study
pGD72-3	pBAD::CsrA$_{Cj}$-Strep, based on pGD68-1	pBR322/ Amp^R	This study
pGG1	Plasmid (based on pZE12-luc) harbouring 3xFLAG and non-polar *aphA-3* cassette. Used for introduction of 'UP' and 'DN' regions of a gene of interest to be FLAG-tagged	ColE1/ Kan^R Amp^R	Sharma lab
pGD78-1	*aphA -3* ORF in pGG1 replaced by *aac*(*3*)-*IV* ORF	ColE1/ Gm^R Amp^R	This study
pGD4-1	Plasmid harbouring *csrA*-3xFLAG C-terminal translational fusion, *csrA* upstream and downstream regions, and *aphA-3* cassette in pGG1 for chromosomal epitope tagging at native locus	ColE1/ Kan^R Amp^R	This study
pMW5-2	Plasmid harbouring *flaA*-3xFLAG C-terminal translational fusion, *flaA* upstream and downstream regions, and *aphA-3* cassette in pGG1 for chromosomal epitope tagging at native locus	ColE1/ Kan^R Amp^R	This study
pMW6-1	Plasmid harbouring *flaB*-3xFLAG C-terminal translational fusion, *flaB* upstream and downstream regions, and *aphA-3* cassette in pGG1 for chromosomal epitope tagging at native locus	ColE1/ Kan^R Amp^R	This study
pSSv1-2	Plasmid harbouring *flgI*-3xFLAG C-terminal translational fusion, *flgI* upstream and downstream regions, and *aphA-3* cassette in pGG1 for chromosomal epitope tagging at native locus	ColE1/ Kan^R Amp^R	This study
pGD70-5	Plasmid harbouring 1,100 bp region around *flaA* promoter; based on pJV752.1	p15A mod/ Amp^R	This study
pGD76-1	*aac*(3)-IV gentamicin cassette introduced upstream of *flaA* promoter in pGD70-5 in reverse orientation to *flaA*	p15A mod/ Gm^R Amp^R	This study
pGD92-1	M1 (SL1 GGA>AAA) mutation in *flaA* 5'UTR in pGD76-1	p15A mod/ Gm^R Amp^R	This study
pGD77-1	M2 (SL1 GGA>UGA) mutation in *flaA* 5'UTR in pGD76-1	p15A mod/ Gm^R Amp^R	This study
pGD93-1	M3 (SL2 GGA>GGG) mutation in *flaA* 5'UTR in pGD76-1	p15A mod/ Gm^R Amp^R	This study
pGD95-1	M2 (SL1 GGA>UGA) /M3 (SL2 GGA>GGG) mutation in *flaA* 5'UTR in pGD76-1	p15A mod/ Gm^R Amp^R	This study
pGD114-2	Start codon mutation in *flaA* (AUG→AAG) in pGD76-1	p15A mod/ Gm^R Amp^R	This study
pGD205-1	Start codon mutation in *flaA* (AUG→AUU) in pGD76-1	p15A mod/ Gm^R Amp^R	This study
pGD204-1	Start codon mutation in *flaA* (AUG→GUG) in pGD76-1	p15A mod/ Gm^R Amp^R	This study
pGD206-1	3rd codon mutation in *flaA* (AUG→UAG) in pGD76-1	p15A mod/ Gm^R Amp^R	This study
pGD207-1	3rd codon mutation in *flaA* (AUG→UUC) in pGD76-1	p15A mod/ Gm^R Amp^R	This study
pGD208-1	101st codon mutation in *flaA* (CAA→UAA) in pGD76-1	p15A mod/ Gm^R Amp^R	This study
pGD209-1	*flaA* promoter replaced by *metK* promoter in pGD76-1	p15A mod/	This study

		Gm^R Amp^R	
pGD107-1	*aac(3)*-IV gentamicin cassette replaced by *cat* cassette in pGD92-1	p15A mod/ Cm^R Amp^R	This study
pGD31-1	5'UTR along with first 33 codons of *flaA* fused in-frame to *gfp* in pXG-10	pSC101*/ Cm^R	This study
pGD111-1	5'UTR along with first 25 codons of *flaG* fused in-frame to *gfp* in pXG-10	pSC101*/ Cm^R	This study
pGD38-1	5'UTR along with first 35 codons of *flgI* fused in-frame to *gfp* in pXG-10	pSC101*/ Cm^R	This study
pGD109-1	5'UTR along with first 35 codons of *flaB* fused in-frame to *gfp* in pXG-10	pSC101*/ Cm^R	This study
pGD112-1	5'UTR along with first 26 codons of *pseB* fused in-frame to *gfp* in pXG-10	pSC101*/ Cm^R	This study
pGD110-1	5'UTR along with first 16 codons of Cj1249 fused in-frame to *gfp* in pXG-10	pSC101*/ Cm^R	This study
pGD28-1	Last 17 codons of Cj0310c and first 23 codons of Cj0309c fused in-frame to FLAG-*lacZ* and *gfp,* respectively, in pXG-30	pSC101*/ Cm^R	This study
pGD27-1	Last 17 codons of Cj0805 and first 25 codons of *dapA* fused in-frame to FLAG-*lacZ* and *gfp,* respectively, in pXG-30	pSC101*/ Cm^R	This study

Supplementary Table 6. Construction of *C. jejuni* mutants.

Mutation	'UP' PCR primers	'DN' PCR primers	Cassette/ Primers	UP-cassette-DN amplification primers	Mutant validation primers
Single gene deletions (overlap PCR)					
**Δ***csrA* (Cm[R])	CSO-0394 CSO-0615	CSO-0616 CSO-0395	*cat* (CSO-0613/-0614)	CSO-0394 CSO-0395	CSO-0392 CSO-0614
**Δ***csrA* (Hyg[R])	CSO-0393 CSO-0394	CSO-0395 CSO-0396	*aph(7")* (CSO-1678/-1679)	CSO-0394 CSO-0395	CSO-0392 HPK2
**Δ***fliW* (Gm[R])	CSO-0997 CSO-0998	CSO-0999 CSO-1000	*aac*(3)-IV (HPK1/HPK2)	CSO-0997 CSO-1000	CSO-1072 HPK2
**Δ***fliW* (Hyg[R])	CSO-0997 CSO-0998	CSO-0999 CSO-1000	*aph(7")* (CSO-1678/-1679)	CSO-0997 CSO-1000	CSO-1072 HPK2
**Δ***flaA* (Kan[R])	CSO-0752 CSO-1548	CSO-1549 CSO-1550	*aphA-3* (HPK1/HPK2)	CSO-0999 CSO-1000	CSO-0756 CSO-0023
**Δ***flaB* (Kan[R])	CSO-2842 CSO-2843	CSO-2844 CSO-0560	*aphA-3* (HPK1/HPK2)	CSO-0999 CSO-1000	CSO-2841 CSO-0023
**Δ***flaAB* (Kan[R])	CSO-0752 CSO-1548	CSO-1549 CSO-1550	*aphA-3* (HPK1/HPK2)	CSO-0999 CSO-1000	CSO-0756 CSO-0023
**Δ***rpoN* (Gm[R])	CSO-1144 CSO-1145	CSO-1146 CSO-1147	*aac*(3)-IV (HPK1/HPK2)	CSO-1144 CSO-1147	CSO-1148 HPK2
**Δ***fliA* (Gm[R])	CSO-1149 CSO-1150	CSO-1151 CSO-1152	*aac*(3)-IV (HPK1/HPK2)	CSO-1149 CSO-1152	CSO-1153 HPK2
3xFLAG tags (cloned in pGG1)					
csrA-3xFLAG (NCTC11168 and 81-176) (pGD4-1)	CSO-0171 CSO-0172	CSO-0173 CSO-0174	*aphA-3* (from pGG1)	CSO-0172 CSO-0173	CSO-0023 CSO-0196
flaA-3xFLAG (pMW5.2)	CSO-0553 CSO-0554	CSO-0555 CSO-0556	*aphA-3* (from pGG1)	CSO-0554 CSO-0555	CSO-0023 CSO-0557
flgI-3xFLAG (pSSv1.2)	CSO-1011 CSO-1012	CSO-1013 CSO-1014	*aphA-3* (from pGG1)	CSO-1012 CSO-1013	CSO-1015 HPK2
flaB-3xFLAG (pMW6.1)	CSO-0558 CSO-0559	CSO-0560 CSO-0561	*aphA-3* (from pGG1)	CSO-0559 CSO-0560	CSO-0562 HPK2
3xFLAG tags (overlap PCR)					
flaG-3xFLAG	CSO-1002 CSO-1098	CSO-1099 CSO-1003	*aphA-3* (HPK1/HPK2)	CSO-1002 CSO-1003	CSO-1005 HPK2
Cj0529-3xFLAG	CSO-1408 CSO-1409	CSO-1410 CSO-1411	*aphA-3* (HPK1/HPK2)	CSO-1409 CSO-1410	CSO-1407 CSO-0023
For construction of *flaA* 5'UTR point mutations refer to Methods.					

Supplementary Table 7. GFP fusions for validating CsrA-target interactions in *E. coli*.

Plasmid	*C. jejuni* target gene(s)	Primers used for target amplification	Plasmid backbone	Colony PCR	Description
pGD31-1	*flaA*	CSO-0621<br>CSO-0622	pXG-10	CSO-0621<br>CSO-0155	5'UTR along with first 33 codons of *flaA* fused in-frame to *gfp*
pGD111-1	*flaG*	CSO-1823<br>CSO-1824	pXG-10	CSO-1823<br>CSO-0155	5'UTR along with first 25 codons of *flaG* fused in-frame to *gfp*
pGD38-1	*flgI*	CSO-0694<br>CSO-0695	pXG-10	CSO-0694<br>CSO-0155	5'UTR along with first 35 codons of *flgI* fused in-frame to *gfp*
pGD109-1	*flaB*	CSO-1815<br>CSO-1816	pXG-10	CSO-1815<br>CSO-0155	5'UTR along with first 35 codons of *flaB* fused in-frame to *gfp*
pGD112-1	*pseB*	CSO-1825<br>CSO-1826	pXG-10	CSO-1825<br>CSO-0155	5'UTR along with first 26 codons of *pseB* fused in-frame to *gfp*
pGD110-1	Cj1249	CSO-1819*<br>CSO-1820*	pXG-10	CSO-1819<br>CSO-0155	5'UTR along with first 16 codons of Cj1249 fused in-frame to *gfp*
pGD28-1	Cj0310c-Cj0309c	CSO-0608<br>CSO-0609	pXG-30	CSO-0608<br>CSO-0155	Last 17 codons of Cj0310c and first 23 codons of Cj0309c were fused in-frame to FLAG-*lacZ* and *gfp*, respectively.
pGD27-1	Cj0805-*dapA*	CSO-0606<br>CSO-0607	pXG-30	CSO-0606<br>CSO-0155	Last 17 codons of Cj0805 and first 25 codons of *dapA* were fused in-frame to FLAG-*lacZ* and *gfp*, respectively

Sequencing on plasmids was performed using oligonucleotide CSO-0155 which binds antisense to *gfp.*

*CSO-1819/-1820 were annealed together to yield the insert (without PCR) for direct introduction into pXG-10.

Supplementary Table 8. Details of RNAs used for *in vitro* work. WT GGA motifs are marked in blue and introduced point mutations in the leader variants are marked in red. Start (ATG) and stop (TAA) codons are underlined.

Name	DNA template (plasmid or gDNA)	Primers	Size of T7-transcript [nt]	Sequence (5' → 3')
flaA WT leader	gDNA NCTC11168	CSO-0709<br>CSO-0710	144	UAACAAGUUCAUGGAUGAGCUUGAAUUUUUUUAAAAGGAUUUAAAA<br>UGGGAUUUCGUAUUAACACCAAUGUUGCAGCUUUAAAUGCAAAAGC<br>AAACGCUGAUUUAAAUAGUAAAAGUUUAGAUGCUUCUUUAAGCAGAC<br>UUAGU
flaA M1 leader	pGD92-1	CSO-1656<br>CSO-0710	144	UAACAAGUUCAUAAAUGAGCUUGAAUUUUUUUAAAAGGAUUUAAAA<br>UGGGAUUUCGUAUUAACACCAAUGUUGCAGCUUUAAAUGCAAAAGC<br>AAACGCUGAUUUAAAUAGUAAAAGUUUAGAUGCUUCUUUAAGCAGAC<br>UUAGU
flaA M2 leader	pGD77-1	CSO-1081<br>CSO-0710	144	UAACAAGUUCAUUGAUGAGCUUGAAUUUUUUUAAAAGGAUUUAAAA<br>UGGGAUUUCGUAUUAACACCAAUGUUGCAGCUUUAAAUGCAAAAGC<br>AAACGCUGAUUUAAAUAGUAAAAGUUUAGAUGCUUCUUUAAGCAGAC<br>UUAGU
flaA M3 leader	pGD93-1	CSO-0709<br>CSO-0710	144	UAACAAGUUCAUGGAUGAGCUUGAAUUUUUUUAAAAGGGUUUAAAA<br>UGGGAUUUCGUAUUAACACCAAUGUUGCAGCUUUAAAUGCAAAAGC<br>AAACGCUGAUUUAAAUAGUAAAAGUUUAGAUGCUUCUUUAAGCAGAC<br>UUAGU
flaA M2/M3 leader	pGD95-1	CSO-1081<br>CSO-0710	144	UAACAAGUUCAUUGAUGAGCUUGAAUUUUUUUAAAAGGGUUUAAAA<br>UGGGAUUUCGUAUUAACACCAAUGUUGCAGCUUUAAAUGCAAAAGC<br>AAACGCUGAUUUAAAUAGUAAAAGUUUAGAUGCUUCUUUAAGCAGAC<br>UUAGU
flgI leader	gDNA NCTC11168	CSO-0713<br>CSO-0714	128	ACAAUAGAUUAAAGGAAGAAUCCAUGAGAGUUUUAACGAUAUUUUUA<br>CUCUUUAUGACAAGCAUUUUUGCAGUGCAAAUCAAGGAUGUAGCAA<br>AUACUGUAGGUGUAAGAGAUAACCAACUUAUAGGU
flaG leader	gDNA NCTC11168	CSO-1084<br>CSO-1085	108	ACUAGCAAUAGGAAAUUUUAAAAAGGAUUUUAAAAUGGAAAUAUCGA<br>AGGCAAAUGGGCAAAUGGAUACAGCUUUGGCAAACAUUAGCCAAAG<br>AACAAGUGAGACACA
flaB leader	gDNA NCTC11168	CSO-1817<br>CSO-1818	132	CGAUGCAAUAUUUUGAAAGGAUUUAAAAUGGGUUUUAGGAUAAACA<br>CCAACAUCGGUGCAUUAAAUGCACAUGCAAAUUCAGUUGUUAAUGC<br>UAGAGAACUGGAUAAGUCUUUAAGCAGACUUAGUUCAGGU
Cj0040 leader	gDNA NCTC11168	CSO-1082<br>CSO-1083	117	AAAUUAAAAUUUUAAAAAGGAAGUUAAAAUGUCAAAACCAUUAAAUG<br>AAGAGAUUUUUGUUGAAUUUAAAAGUGAUCUAGCUGAAAGAAAAAAU<br>GAAGUUUUGCUUCAAGUUUUAGA
flgA 3'end	gDNA NCTC11168	CSO-1088<br>CSO-1089	113	CAAAUGUUGAUGUUUUAAUCGAACUUGUGGCUUUGCAAAGUGCAAA<br>UAUGGGCGAAAGGAUUCGUGCAAAAAACAAAGAAGGUAAAGUUAUG<br>CAAGGUAUCGUAGUGGGUAAA
flgM leader	gDNA NCTC11168	CSO-1092<br>CSO-1093	97	AGGAUUAUAACUAAGAUCAAGGAGGCAGAAAUGAUCAAUCCUAUACA<br>ACAAAGUUAUGUGGCAAAUACCGCAUUAAAUACAAAUAGAAUAGAUA<br>AAG
Cj1324 leader	gDNA NCTC11168	CSO-1665<br>CSO-1666	100	AUUUUUAUUAAAUUGAAGGGGUGGGGAAUGAUUUAUUGUGAUCACU<br>GCGUGAUGCCAAAUACUAGACCUGGUAUUAAUUUUACAAAAGAUAAA<br>GAAGGUA
hopB 3'end with UTR	gDNA *H. pylori* G27	CSO-0701<br>CSO-0702	107	AAAGCUGGUGGCGCUGAAGUGAAAUACUUCCGCCCUUAUAGCGUGU<br>AUUGGGUCUAUGGCUACGCCUUCUAAAAAAGCUCAAGGCCUUUUAU<br>AGGCUUUGAUUUAAC

Supplementary Methods

Transformation of *C. jejuni* for mutant construction. Transformation of *C. jejuni* was performed by electroporation or natural transformation as described previously[2, 9, 10, 11]. For electroporation, strains grown from frozen stocks until passage one or two on MH agar were harvested into cold electroporation buffer (272 mM sucrose, 15% v/v glycerol) and washed twice with the same buffer. Cells (50 µl) were mixed with 200-400 ng PCR product on ice and electroporated (Biorad MicroPulser) in a 1 mm gap cuvette (PEQLAB) at 2.5 kV. Cells were then transferred with Brucella broth to a non-selective MH plate and recovered overnight at 37 °C microaerobically before plating on the appropriate selective medium.

In some cases, *C. jejuni* double or triple mutants were constructed by natural transformation of the genomic DNA from the appropriate donor strain[10, 12]. Genomic DNA was extracted from the donor strain by phenol-chloroform extraction and ethanol precipitation. Specifically, bacteria were harvested from one-day-old selective MH plates into SET buffer (150 mM NaCl, 15 mM EDTA, 10 mM Tris-HCl; pH 8.0), collected by centrifugation, and resuspended in SET buffer. SDS and proteinase K were then added to final concentrations of 0.5% (w/v) and 100 µg/ml, respectively, and suspensions were incubated at 55 °C for 2h. Protein was then removed by extraction with an equal volume of phenol-chloroform-isoamyl alcohol (25:24:1), separation of phases by centrifugation at 13,000 rpm for 8 min, and re-extraction of the aqueous phase with an equal volume of chloroform with centrifugation at 13,000 rpm to separate phases. DNA was then precipitated from the final aqueous phase with 1/10 vol. 3M sodium acetate, pH 5.3 and 2 vol. absolute ethanol. After overnight incubation at -20 °C, precipitated DNA was collected by centrifugation at 13,000 rpm for 10 min and washed once with 75% cold ethanol. DNA pellets were resuspended in 100 µl water with shaking at 65 °C. For transformations, recipient strains were grown from frozen stocks, patched into small circles on a non-selective MH plate, and grown for 2-3 h at 37 °C under microaerobic conditions. One hundred ng of donor genomic DNA (gDNA) was then added to the patches and plates were incubated for an additional 4-5h. Patched cells were then harvested into 1 ml Brucella broth and 10 or 100 µl was plated on the appropriate selective MH agar. Colonies were re-streaked onto selective plates, and colony PCR was performed to confirm presence of desired mutations from both donor and recipient strain.

Construction of *C. jejuni* deletion strains by overlap PCR. All *C. jejuni* deletion mutant strains listed in Supplementary Table 3 were generated by double-crossover homologous recombination with PCR products of deletion cassettes that were constructed by overlap PCR (for details see Supplementary Table 6) and electroporated into bacteria as described above.

PCR products carried *aphA-3* kanamycin[13], *C. coli cat* chloramphenicol[14], *aac*(3)-IV gentamicin[6], or *aph*(7″) hygromycin[7] resistance cassettes flanked by ~500 bp of homologous sequence up- and downstream of the coding region of the target gene. Non-polar resistance cassettes were amplified from plasmids that carry the resistance markers using primers HPK1/HPK2 (Kan^R), CSO-1678/-1679 (Hyg^R), or CSO-0613/-0614 (Cm^R). The *aphA-3* (Kan^R) ORF was replaced by the *aac*(3)-IV (Gm^R) ORF (amplified using CSO-0575/-0832 and *Nde*I digested) in the plasmid pGG1 (amplified using CSO-0577/-0831 and *Nde*I digested) leaving the HPK1/HPK2 binding sites intact. The resulting plasmid pGD78-1 was used to amplify the *aac*(3)-IV (Gm^R) cassette using the same HPK1/HPK2 primers.

As an example, the construction of the chloramphenicol resistant *C. jejuni* NCTC11168 Δ*csrA*::*cat* deletion mutant is described. About 500 bp upstream of the *csrA* (Cj1103) start codon was amplified from genomic DNA (gDNA) of *C. jejuni* NCTC11168 WT using 'UP' primers (CSO-0394/-0615). Likewise, ~500bp downstream of the *csrA* stop codon was amplified using 'DN' primers (CSO-0616/-0395). The 5' ends of the antisense-UP primer and sense-DN primer contained ~25 bp of sequence homologous to the sense or antisense primer (CSO-0613/-0614), respectively, used to amplify the *cat* resistance cassette. PCR products were purified (Macherey-Nagel NucleoSpin PCR cleanup kit), and UP, DN, and resistance cassette amplicons were then added together in a ratio of 50:50:90 ng to a 100 µl Phusion polymerase PCR reaction with sense-UP and antisense-DN primers (CSO-0394/-0395) at a final concentration of 0.06 µM. Overlap PCR was performed with the following conditions: 1 cycle of [98 °C, 3 min; 61 °C, 1 min; 72 °C, 10 min; 98 °C, 1 min], 40 cycles of [98 °C, 15 s; 57 °C, 20 s; 72 °C, 1 min], followed by a 10 min final extension at 72 °C. Following verification of product size by agarose gel electrophoresis and purification (Macherey-Nagel NucleoSpin PCR cleanup kit), the resulting overlap PCR product was electroporated into the appropriate recipient *C. jejuni* strain. Deletion mutants for *flaA*::Kan^R, *flaB*::Kan^R, *flaAB*::Kan^R, *fliW*::Gm^R, *fliW*::Hyg^R *fliA*::Gm^R, *rpoN*::Gm^R, and *csrA*::Hyg^R were constructed similarly (see Supplementary Table 6).

Sequence and structure conservation of the *flaA* 5'UTR. In order to identify homologous *flaA* 5'UTR regions in different *Campylobacter* species and strains we ran nucleotide blast (blastn[15]) with parameters optimized for more dissimilar sequences (discontiguous megablast) using both the NCBI nucleotide collection (nr/nt) and the NCBI whole genome shotgun (wgs) contigs as databases. As query we used a 130 nt-long sequence encompassing the 100 nt upstream and the first 30 nt of the *C. jejuni* NCTC11168 *flaA* coding region. This includes the *flaA* promoter region, its 5'UTR, and the beginning of the coding sequence. Based on all hits (~200) we extracted the 130 nt from the target sequences. If BLAST hits with an optimal score were truncated they were extended on either side to obtain 130 nt. We excluded all sequences with undefined bases or

without proper species/strain association. Afterwards, sequences for *C. fetus* subsp. *fetus* 82-40 and *C. concisus* 13826, which were not found by BLAST, were added manually to the set. The sequences were aligned using MUSCLE[16] with default parameters and a conserved Sigma28 (FliA) -10 box (CGATAT) was observed in all of them. The alignment was trimmed to the region including only the *flaA* 5'UTR (based on the region according to the 5' UTR of *C. jejuni* NCTC11168) and the first 10 nt of the coding sequence. More dissimilar sequences disrupting the alignment (*C. peloridis* LMG 23910, *C. lari* NCTC11845, *C. lari* NCTC12892, *C. lari* RM16701, *C. lari* CCUG 22395, *C. lari* RM16712, *C. curvus* 525.92, *Campylobacter* sp. FOBRC14 ctg120009214739, *C. curvus* DSM 6644 C514DRAFT scaffold00004.4_C, and *C. concisus* 13826) were removed. Based on the resulting alignment all identical sequences were collapsed keeping only one representative sequence per cluster. Subsequently, a consensus structure was predicted using RNAalifold[17] with RIBOSUM scoring and default values for all other parameters. The resulting structure-annotated sequence alignment is shown in Supplementary Fig. 6 and the full alignment including additional strains is shown in Supplementary Fig. 7.

Supplementary References

1. Gundogdu O, Bentley SD, Holden MT, Parkhill J, Dorrell N, Wren BW. Re-annotation and re-analysis of the Campylobacter jejuni NCTC11168 genome sequence. *BMC Genomics* 2007, **8:** 162.

2. Dugar G, Herbig A, Forstner KU, Heidrich N, Reinhardt R, Nieselt K*, et al.* High-Resolution Transcriptome Maps Reveal Strain-Specific Regulatory Features of Multiple Campylobacter jejuni Isolates. *PLoS Genet* 2013, **9**(5)**:** e1003495.

3. Urban JH, Vogel J. Translational control and target recognition by Escherichia coli small RNAs in vivo. *Nucleic Acids Res* 2007, **35**(3)**:** 1018-1037.

4. Zhang B, Rapolu M, Liang Z, Han Z, Williams PG, Su WW. A dual-intein autoprocessing domain that directs synchronized protein co-expression in both prokaryotes and eukaryotes. *Scientific reports* 2015, **5:** 8541.

5. Sharma CM, Darfeuille F, Plantinga TH, Vogel J. A small RNA regulates multiple ABC transporter mRNAs by targeting C/A-rich elements inside and upstream of ribosome-binding sites. *Genes Dev* 2007, **21**(21)**:** 2804-2817.

6. Bury-Mone S, Skouloubris S, Dauga C, Thiberge JM, Dailidiene D, Berg DE*, et al.* Presence of active aliphatic amidases in Helicobacter species able to colonize the stomach. *Infect Immun* 2003, **71**(10)**:** 5613-5622.

7. Cameron A, Gaynor EC. Hygromycin B and apramycin antibiotic resistance cassettes for use in Campylobacter jejuni. *PLoS One* 2014, **9**(4)**:** e95084.

8. Lutz R, Bujard H. Independent and tight regulation of transcriptional units in Escherichia coli via the LacR/O, the TetR/O and AraC/I1-I2 regulatory elements. *Nucleic Acids Res* 1997, **25**(6)**:** 1203-1210.

9. Hansen CR, Khatiwara A, Ziprin R, Kwon YM. Rapid construction of Campylobacter jejuni deletion mutants. *Lett Appl Microbiol* 2007, **45**(6)**:** 599-603.

10. McLennan MK, Ringoir DD, Frirdich E, Svensson SL, Wells DH, Jarrell H*, et al.* Campylobacter jejuni biofilms up-regulated in the absence of the stringent response utilize a calcofluor white-reactive polysaccharide. *J Bacteriol* 2008, **190**(3)**:** 1097-1107.

11. Miller JF, Dower WJ, Tompkins LS. High-voltage electroporation of bacteria: genetic transformation of Campylobacter jejuni with plasmid DNA. *Proc Natl Acad Sci U S A* 1988, **85**(3)**:** 856-860.

12. Wassenaar TM, Fry BN, van der Zeijst BA. Genetic manipulation of Campylobacter: evaluation of natural transformation and electro-transformation. *Gene* 1993, **132**(1)**:** 131-135.

13. Skouloubris S, Thiberge JM, Labigne A, De Reuse H. The Helicobacter pylori UreI protein is not involved in urease activity but is essential for bacterial survival in vivo. *Infect Immun* 1998, **66**(9)**:** 4517-4521.

14. Boneca IG, Ecobichon C, Chaput C, Mathieu A, Guadagnini S, Prevost MC, *et al.* Development of inducible systems to engineer conditional mutants of essential genes of Helicobacter pylori. *Appl Environ Microbiol* 2008, **74**(7)**:** 2095-2102.

15. Altschul SF, Gish W, Miller W, Myers EW, Lipman DJ. Basic local alignment search tool. *J Mol Biol* 1990, **215**(3)**:** 403-410.

16. Edgar RC. MUSCLE: multiple sequence alignment with high accuracy and high throughput. *Nucleic Acids Res* 2004, **32**(5)**:** 1792-1797.

17. Bernhart SH, Hofacker IL, Will S, Gruber AR, Stadler PF. RNAalifold: improved consensus structure prediction for RNA alignments. *BMC Bioinformatics* 2008, **9:** 474.
